# Tools for measuring gender equality and women’s empowerment (GEWE) indicators in humanitarian settings

**DOI:** 10.1186/s13031-021-00373-6

**Published:** 2021-05-17

**Authors:** Céline M. Goulart, Amber Purewal, Humaira Nakhuda, Anita Ampadu, Amanda Giancola, Jean-Luc Kortenaar, Diego G. Bassani

**Affiliations:** The Hospital for Sick Children, 686 Bay St, Toronto, ON M5G 0A4 Canada

**Keywords:** Girls and women, Humanitarian, Measurement methods, Gender equality, Women’s empowerment

## Abstract

**Background:**

Effective measurement of Gender Equality and Women’s Empowerment (GEWE) is challenging in low and middle-income countries (LMICs), and even more so in humanitarian settings. Conflict, natural disasters, and epidemics may increase gender inequities, but also present an opportunity to address them. This scoping review describes and identifies gaps in the measurement tools, methods, and indicators used to measure GEWE in humanitarian settings, and presents a dashboard that can be used by researchers, organizations and governments to identify GEWE measurement tools.

**Methods:**

Scientific articles published between January 2004 and November 2019 were identified using Embase, Medline, PsycInfo, CINAHL, Scopus, and PAIS index. Relevant non peer-reviewed literature was downloaded from the websites of humanitarian organizations. Publications on women and/or girls impacted by a humanitarian crisis in a LMIC, within 5 years of data collection, were included. Publications were double-screened in the title/abstract and full-text stages. We used a machine learning software during the title/abstract screening to increase the efficiency of the process. Measurement tools, sampling and data collection methods, gap areas (geographical, topical and contextual), and indicators were catalogued for easy access in an interactive Tableau dashboard.

**Results:**

Our search yielded 27,197 publications and 2396 non peer-reviewed literature reports. One hundred and seventy publications were included in the final review. Extracted indicators were categorized into seven domains: economic, health, human development, leadership, psychological, security and justice, and sociocultural. The vast majority of studies were observational, and over 70% utilized a cross-sectional study design. Thirty-eight toolkits and questionnaires were identified in this review, of which 19 (50%) were designed specifically for humanitarian settings. Sociocultural was the largest domain in number of studies and indicators in this review, with gender-based violence indicators reported in 66% of studies. Indicators of economic, human development and leadership were uncommon in the peer-reviewed literature.

**Discussion:**

While there has been some effort to measure GEWE in conflict-affected and other humanitarian settings, measurement has largely focused on violence and security issues. A more comprehensive framework for measuring GEWE in these settings is needed; objective measurement of women’s empowerment and gender equality should be prioritized by organizations providing humanitarian aid.

**Supplementary Information:**

The online version contains supplementary material available at 10.1186/s13031-021-00373-6.

## Background

Achieving gender equality and women’s empowerment are key components of improving the wellbeing of all people [1]. Goal five of the Sustainable Development Goals (SDGs) focuses on achieving gender equality and empowering all women and girls (Goal 5: Achieve gender equality and empower all women and girls). Key targets of this goal include ending discrimination against women and girls, eliminating violence/harmful practices that target women and girls, recognizing the unpaid care/domestic labor performed by women, ensuring equal opportunities for women’s participation and leadership, and securing universal access to sexual and reproductive health and rights (SRHR) for all women and girls [1].

It is estimated that more than 80 million people were forcibly displaced worldwide due to conflict, persecution, and other human rights violations at mid-2020 [2]. These crises disproportionately affect the wellbeing of women and children as a result of pre-existing biological and sociocultural factors [3, 4]. Women may have less access to finances, goods and training to protect themselves during crises [3]. They also bear the majority of the caregiving responsibilities in many of these settings [4]. Women and girls represent just over 50% of the global refugee and IDP populations, yet only 4% of projects in the UN inter-agency appeals targeted women and girls in 2014 [5]. Furthermore, these crises can exacerbate gender inequalities and risks for women and girls [6], making it important to employ gender-appropriate tools when collecting data about women and girls in humanitarian settings. Gender appropriate tools are crucial when seeking to understand gender equality, as they provide sex-disaggregated indicators, along with indicators that are specific to the sociocultural, health and safety needs of women and girls [7].

### Summary of existing literature and framework used

Empowerment is defined as the process of change in one’s ability to exercise choice [8]. Previous research has focused on the impact of gender equality and women’s empowerment (GEWE) on maternal health and family planning (FP), children’s health outcomes, and SRHR. Women’s empowerment is a social determinant of maternal and child health [9]. It is positively associated with women’s contraceptive use in low- and middle-income countries (LMICs) [10] and reductions in under-five mortality [11]. The measurement of GEWE in humanitarian settings has often been approached from a biomedical lens, focusing on SRHR outcomes specifically [12]. However, domains of GEWE, outside of those pertaining to gender-based violence (GBV), have not been routinely or consistently measured in these settings.

Various conceptual frameworks exist for measuring women’s empowerment. Kabeer established three dimensions of empowerment: agency, resources, and achievements [8]. Resources enhance a woman’s ability to exercise choice, while agency refers to her ability to act on those choices. Another conceptual model of empowerment developed by van Eerdewijk et al. also includes agency and resources, but adds institutional structures, to the dimensions [13]. In this model, agency includes decision-making, collective agency, and leadership; resources pertain to women and girls’ bodily integrity, critical consciousness, and assets; and institutional structures refer to the formal laws/policies and norms that impact the ability of women and girls to assert control over resources [13]. Either of the frameworks above may be used to organize measures of GEWE in humanitarian settings.

This scoping review categorizes GEWE indicators into the following domains: economic, health, human development, leadership, psychological, security and justice, and sociocultural. The economic domain captures indicators on employment, financial decision-making, and income generation. The health domain includes indicators on bodily autonomy, health perceptions, and access to services, whereas the human development domain captures access to basic needs, education, and vocational training. Indicators that measure a change/effect in women’s empowerment resulting from an intervention are also categorized under human development. The leadership domain includes indicators on leadership qualities and community participation. The psychological domain captures indicators on women’s self-esteem and social support. The security and justice domain captures laws/policies that affect women, as well as their sense of safety and security in their respective communities. Lastly, the sociocultural domain captures the prevalence of GBV, as well as the cultural norms and attitudes relating to women’s autonomy.

Reliable measurement of gender-relevant indicators in humanitarian settings is difficult, as data is usually unavailable and primary data collection is costly and time-consuming [14]. In order to streamline data collection and other measurement processes in such settings, simpler approaches to valid and reliable gender-relevant indicators need to be identified.

### Objective

A scoping review is a synthesis of current research to map the literature available on a topic of interest, and is therefore useful way to identify the tools and strategies used to measure GEWE in humanitarian settings [15]. It is also a useful way to identify any limitations and gaps in the use of measurement tools, indicators, and data sources [16]. Hence, a scoping review was conducted to systematically map and assess existing indicators, data sources, and methodologies, as well as to identify knowledge gaps in GEWE in humanitarian settings. Results from this review were then used to create an interactive Tableau dashboard.

## Methods

This scoping review was conducted in accordance with the Preferred Reporting Items for Systematic Reviews and Meta-Analyses (PRISMA) statement [17].

### Definitions and inclusion criteria

We used terms associated with our seven domains of gender equality and women’s empowerment to build our search strategy. The term ‘women’ rather than ‘females’ was used, to ensure that persons who identify as women, but were not assigned ‘female’ at birth, were included in this review. We defined a humanitarian crisis as “a serious disruption of the functioning of a community or a society causing widespread human, material, economic or environmental losses which exceed the ability of the affected community or society to cope using its own resources, necessitating a request to national or international level for external assistance. The disaster situation may either be manmade (e.g. armed conflict) or a natural phenomenon (e.g. drought)” [18]. We further stipulated that the humanitarian event had to result in over 1000 human casualties and had to be distinct from latent civil unrest/conflict, or long-standing epidemics, such as HIV. See Additional file 1 for the full search strategy.

For a publication to meet the inclusion criteria, it had to:

- Include women and/or girls that had been impacted by a humanitarian crisis in a low and middle-income country (LMIC). Data must have been collected either during the time of the humanitarian event or no more than 5 years post-event. This included populations who had fled a country with ongoing conflict living in unaffected LMICs. Refugee populations living in high-income countries were excluded.
- Be an interventional or observational study on a GEWE-related topic.
- Be published on or after January 1st, 2004 to November 27th, 2019. Publications before the year 2004 were excluded on the basis of relevance, and for feasibility given the large scope of the review.
- Be in the English language or have an English translation available online.
- Measure quantitative GEWE outcomes.

Qualitative studies or those reporting crude numbers without denominators, and studies without any measurement methods were excluded. See Additional file 2 for detailed inclusion and exclusion criteria.

### Search

On November 27th, 2019, we ran our search strategy on the following databases: Embase, Medline, PsycInfo, CINAHL, Scopus, and PAIS index.

The following websites and databases were searched for relevant non peer-reviewed literature: United Nations Population Fund (UNFPA), United Nations High Commission for Refugees (UNHCR), Oxfam, Care International, UN Women, the World Health Organization (WHO), the United Nations Development Programme (UNDP), International Rescue Committee (IRC), International Committee of the Red Cross (ICRC), World Vision, Médecins Sans Frontières (MSF) Field Research, Active Learning Network for Accountability and Performance (ALNAP), Save the Children International (SCI), Sexual Violence Research Initiatives (SVRI), Inter-Agency Working Group on Reproductive Health in Crises (IAWG), Women’s Refugee Commission, Humanitarian Response, Relief Web, The Gender and Development Network, International Alert, Women Deliver, ProMundo, International Center for Research on Women (ICRW) and Plan International. The first 200 citations from each source were screened. This non peer-reviewed literature search was conducted concurrently with our SRHR scoping review [12], such that SRHR non peer-reviewed literature articles identified as relevant to GEWE in the screening phase could be duplicated in the GEWE non peer-reviewed literature screening process.

### Screening process

In order to accelerate the title and abstract screening phase of the scoping review, we developed a machine learning software to rank the relevance of the abstracts from our database search. Details of our machine learning tool can be found in Additional file 3. Machine learning software was used as it increased the efficiency of the screening process [19].

Additional resources were used to retrieve GEWE publications. First, reference lists from relevant systematic reviews retrieved from our database search were screened. Second, GEWE publications identified in the SRHR search results were transferred to this review. These two procedures contributed an additional 48 publications to the study count.

After the title and abstract screening process, the remaining articles were full text screened by two reviewers for eligibility and assigned an exclusion reason if they did not meet the study criteria.

### Data extraction and analysis

Included studies were extracted in duplicate into a Microsoft Excel spreadsheet. A narrative synthesis [20] was conducted to group the indicators thematically, into seven different domains of equality/empowerment. We sorted indicators into types and subtypes for each domain of GEWE. Extracted and organized data were then cleaned and frequencies were tabulated using STATA: Software for Statistics and Data Science [21]. Demographics, measurement methods and tools, and indicators were counted once per study. These counts were interpreted using the narrative synthesis method, to describe the measurement methods and gaps. The toolkits and surveys employed within the included studies were then organized in a table, found in Additional file 4.

A dashboard was created using Tableau to display the measurement methods, tools, indicators and gaps identified in this study. This dashboard is accessible online, along with the results from our SRHR review [22]. See Additional file 5 to access a link to this dashboard.

## Results

**Fig. 1:**
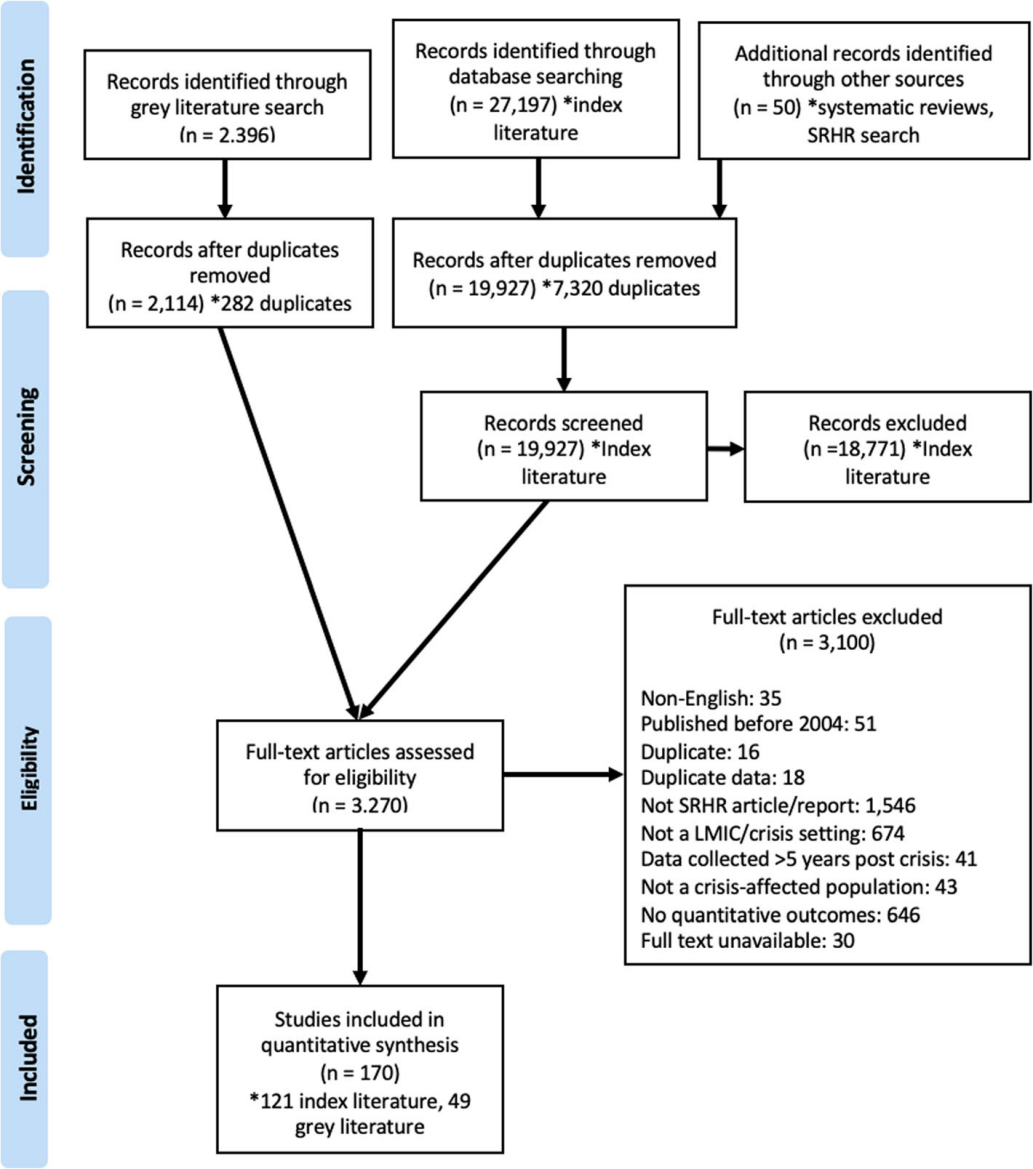
PRISMA chart

Our search yielded 27,197 publications and 2396 non peer-reviewed literature reports. After removing duplicates, conducting the title/abstract and full-text screenings for our inclusion criteria, 170 publications were included in the final review (Fig. 1). See Additional file 6 for the complete list of studies.

### Study characteristics

**Fig. 2:**
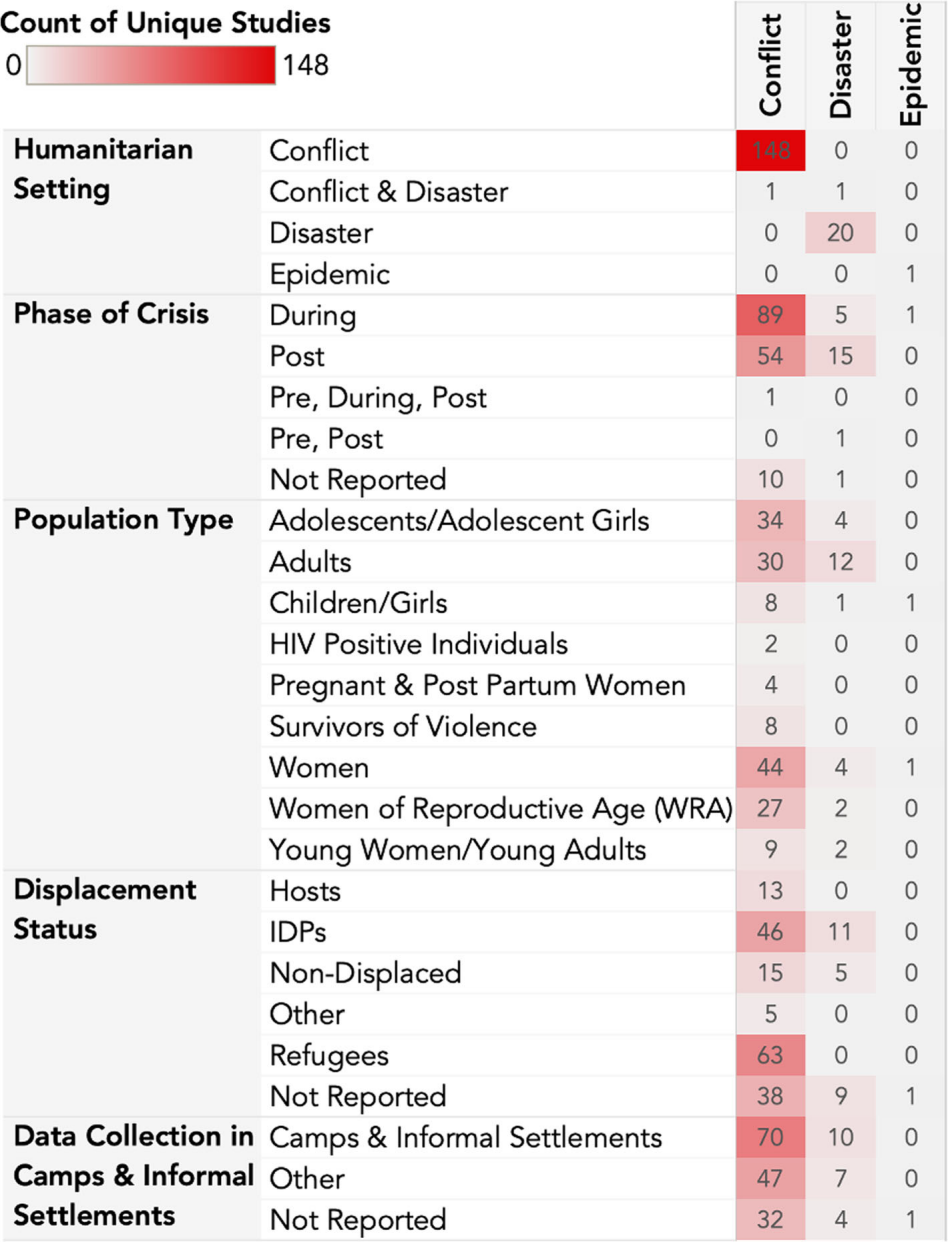
Study Characteristics

**Fig. 3:**
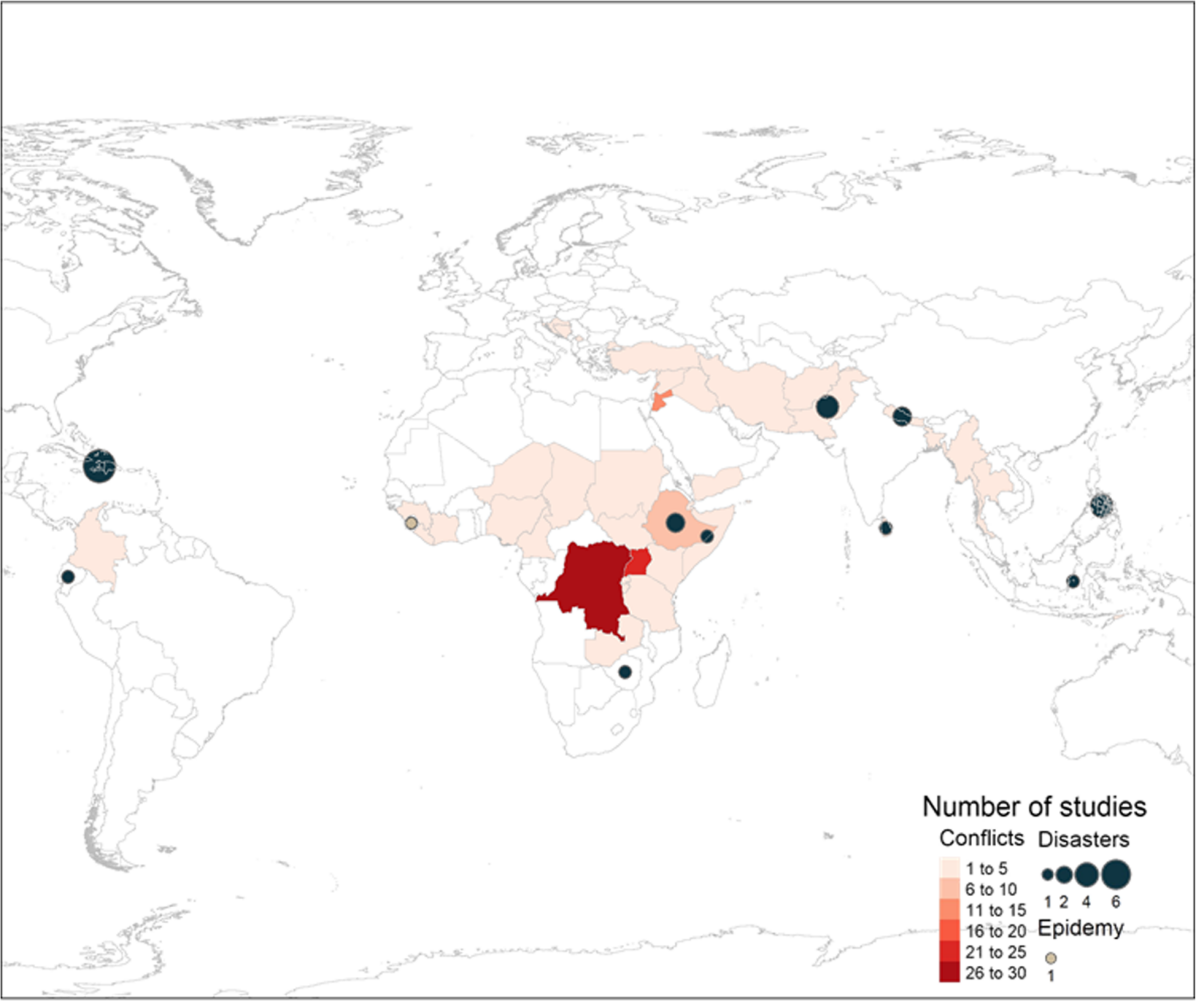
Geographical location and number of studies by type of humanitarian setting

Study characteristics of included papers are listed in Fig. 2. One hundred and forty-eight publications collected data on conflict-affected populations, accounting for 87% of studies in the review. Geographic locations are illustrated in Fig. 3. Forty-five countries were covered by the studies included in this review, with the largest number of studies conducted in the Democratic Republic of the Congo (DRC), Uganda, Jordan, and Ethiopia. Twenty studies (12%) collected data in disaster and post-disaster settings, and only one (0.6%) was in an epidemic setting. The most common disaster setting was Haiti. Of the disaster literature, 8 studies were about earthquakes, 4 about drought, 3 about typhoons, 3 about floods. Additionally, one study was about a tsunami while another was about volcanic disaster. See Additional file 7 for more details. Of the 63 studies that collected data with refugee populations, the majority focused on refugees from Syria, DRC, South Sudan, Sudan and Myanmar (combined total of *n* = 21 (33%)).

**Fig. 4:**
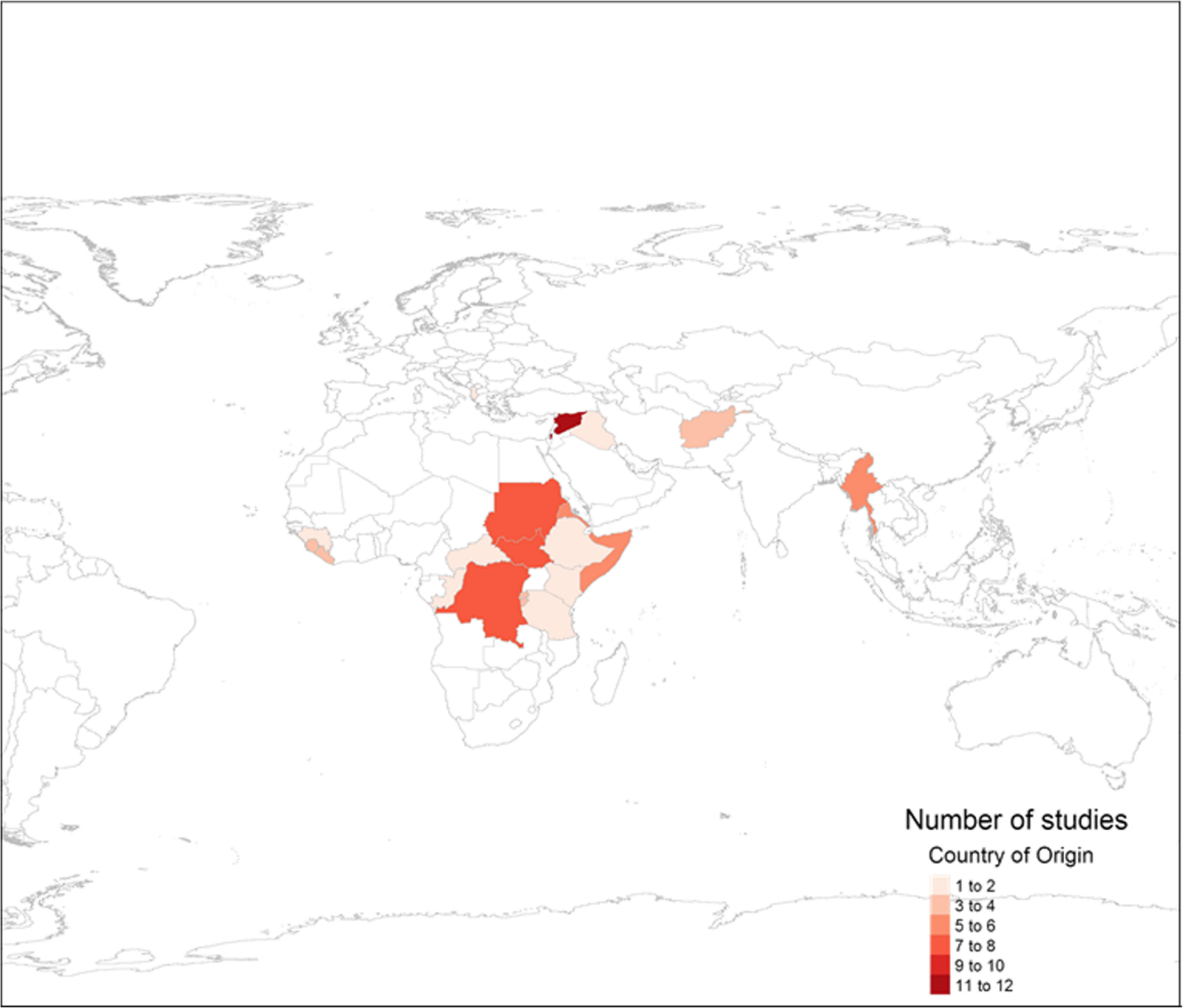
Geographical location and number of studies by country of origin of refugee populations

Forty-six (31%) conflict studies and 11 (55%) disaster studies were conducted among populations of internally displaced persons (IDP). Nearly 50% of studies were with populations residing in camps and informal settlements. Women, adolescents, adults, and women of reproductive age (WRA) made up the majority of the study populations in this review. Children and girls appeared in only ten studies (6%) (Fig. 4).

### Measurement methods used

**Fig. 5:**
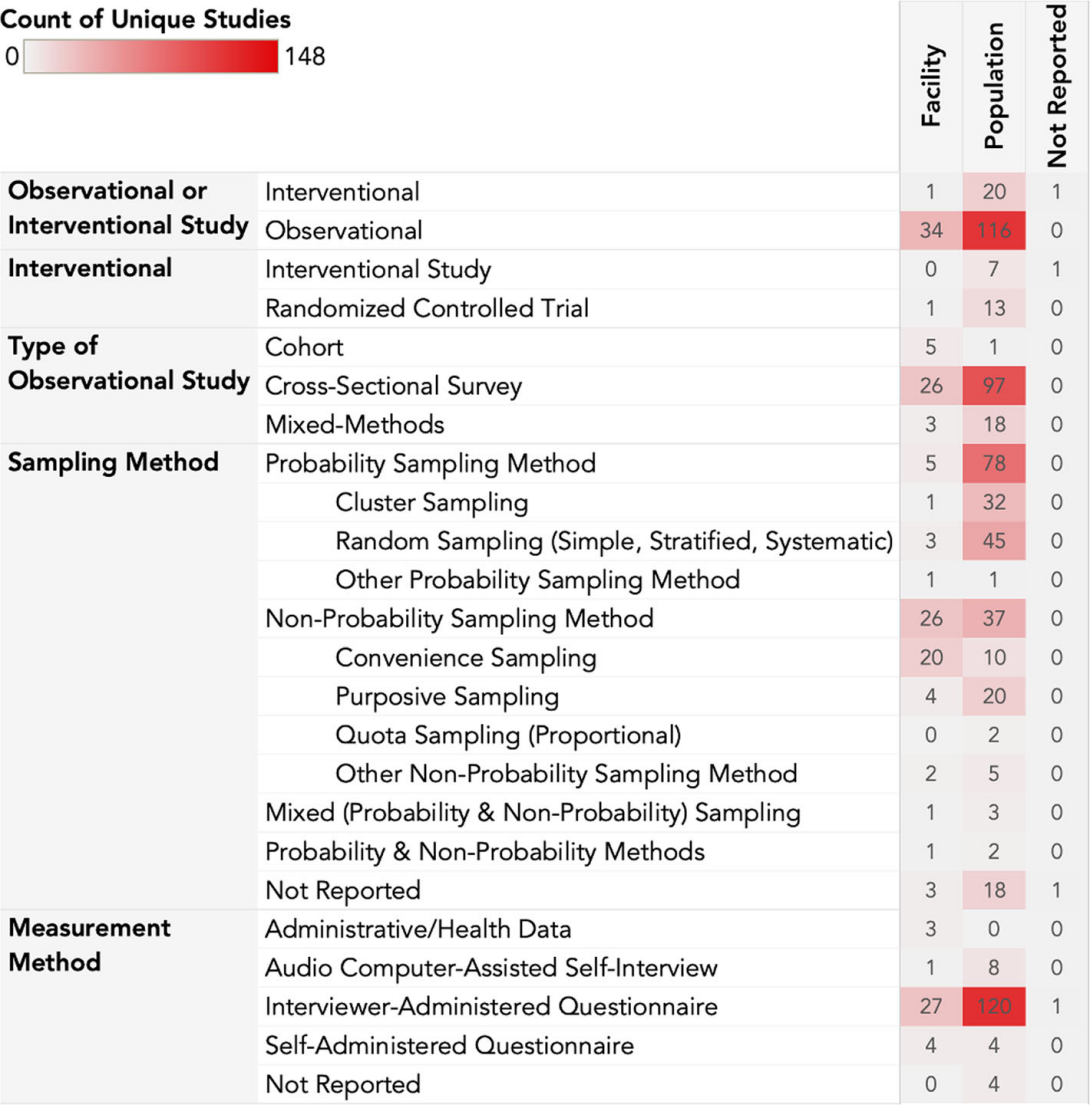
Distribution of the data collection methods

Common measurement methods identified by this review are summarized in Fig. 5. Of 170 studies included in this review, 148 (87.1%) of the studies were observational, and only 22 (12.9%) were interventional. Thirteen (59%) of the interventional studies were randomized controlled trials (RCTs). Over 80% (*n* = 121) of the observational studies were cross-sectional surveys. Thirty-three (19.4%) studies were conducted at the facility-level, while 134 (78.8%) were conducted at the population level. Of all the indicators measured in this study, 36% were sex-disaggregated.

Eighty-three (49%) studies used a probability sampling method. Cluster sampling and random sampling were the most commonly reported probability methods. Sixty-three (37%) studies utilized a non-probability method, with 29 (46%) of these studies using convenience sampling. Two (1%) studies used a combination of probability and non-probability methods, while 22 (13%) studies did not report the sampling method used (Fig. 5). Interviewer-administered questionnaires, self-administered questionnaires, and audio computer-assisted self-interviews (ACASI) were the most commonly used measurement methods (*n* = 146 (86%), *n* = 8 (5%), and *n* = 9 (5%), respectively).

Thirty-eight toolkits were identified in this review, of which 22 (58%) were sex–disaggregated and 19 (50%) were designed specifically for humanitarian settings. The three most commonly used toolkits were the WHO Multi-Country Study on Women’s Health and Domestic Violence Against Women (*n* = 16 (9%)), Demographic and Health Survey (DHS) (*n* = 12 (7%)) and the Centre for Disease Control (CDC) Reproductive Health Assessment Toolkit for Conflict Affected Women (n = 8 (5%)). Refer to Additional file 4 for a full list of toolkits.

### Domains of equality/empowerment

**Fig. 6:**
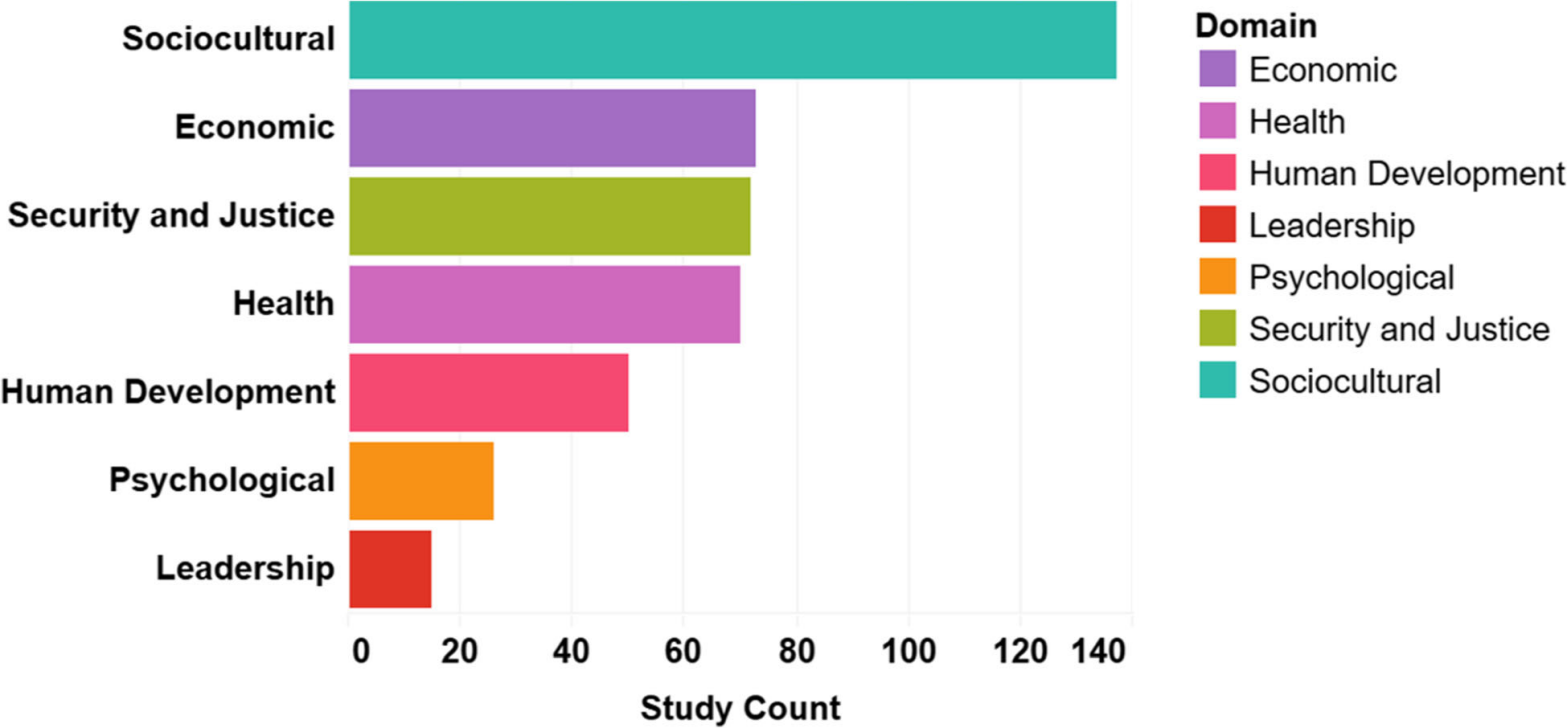
Number of studies by GEWE domain

The sociocultural domain was included in the largest number of studies in this review, appearing in 137 (81%) of 170 studies. The GBV indicator type accounted for 97% of this domain’s indicators (*n* = 133). The security and justice, economic, and health domains were found in 72 (42%), 73 (43%), and 70 (41%) studies, respectively. Fifty (29%) studies included human development, and 25 (15%) included psychological indicators. Leadership was the least frequently identified domain, appearing in only 15 (9%) studies. Figure 6 shows the organization of indicators by domain.

### Gaps in measurement

**Fig. 7:**
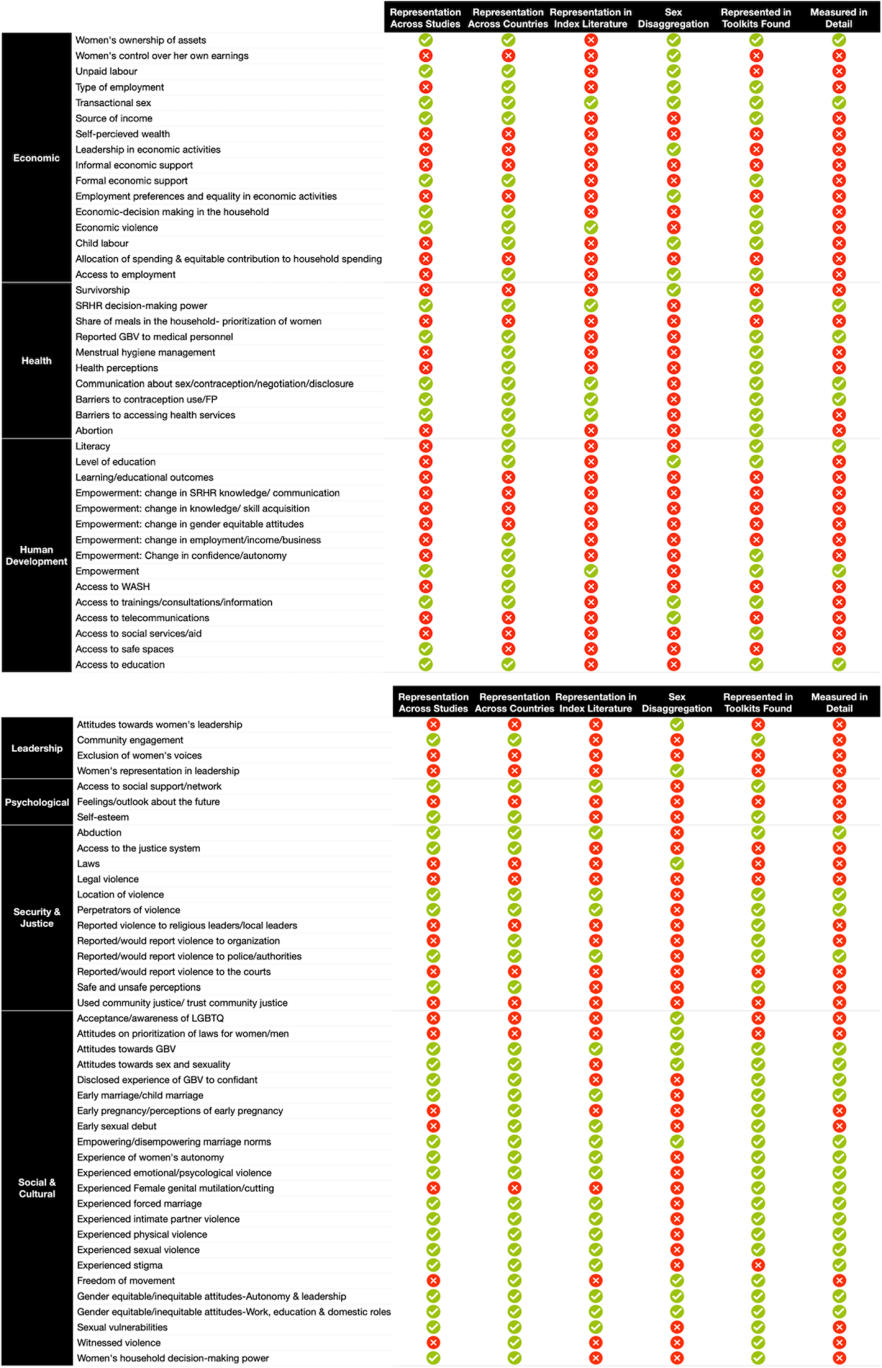
Measurement gap areas

Figure 7 illustrates the various gaps identified by our scoping review. Indicator types/subtypes that were present in ten or more of the 170 papers included in this scoping review *are considered common across studies*. Indicator types/subtypes used in five or more countries *are considered common across countries*. Indicator types/subtypes that were present in five or more studies in the peer-reviewed literature *are considered common in the peer-reviewed literature*. Indicator types/subtypes that are measured among men and women separately in at least 50% of the studies, are considered *sex-disaggregated*. Indicator types/subtypes that are found in three or more toolkits *are considered common across the toolkits*. Finally, an indicator type/subtype is *measured in detail* when it is measured by the same indicator in three or more studies, or three or more different indicators within one study. See Additional file 8 for a full list of indicator types and subtypes.

#### Economic

We found that the economic domain was reported in 78% of the non-peer-reviewed literature but was only found in 29% of studies in the peer-reviewed literature. Although indicators of economic control were captured in humanitarian settings, measures of women’s agency in the economic domain were limited. Indicators measuring women’s allocation of spending were only found in 1% of the studies (*n* = 2), were often not sex-disaggregated, and were not included in toolkit measures. Similarly, indicators about women’s economic decision-making were not commonly measured (*n* = 2, 1%).

Women’s economic decision-making in a leadership and enterprise context (n = 2, 1%) also lacked coverage in the peer-reviewed literature (*n* = 0) and was only measured in the disaster context. Although measurement of the women’s income indicator type was common in humanitarian settings (*n* = 19, 11%), women’s control over their own earnings was not routinely measured (*n* = 3, 2%). The employment indicator type as a whole was found in 18 studies (11%), only 27% of which were peer-reviewed studies. Additionally, measures of informal economic support (IES) were limited in humanitarian contexts and were only captured by two (1%) publications from the non-peer-reviewed literature. Unpaid labor (including men and women’s domestic responsibilities) were found in 18 studies (11%), only 3 of which were peer-reviewed studies.

Transactional sex appeared in 23 publications (14%), including 19 papers in the peer-reviewed literature. Economic violence was measured in 12 studies (7%) across ten countries. Women’s ownership of assets was found in 11 studies (6%), three of which were peer-reviewed studies.

#### Health

Health data lacked sex-disaggregation. SRHR decision-making power (*n* = 20, 12%), barriers to contraception/FP (*n* = 19, 11%) and barriers to accessing health services (*n* = 17, 10%) were less commonly identified. Some topics, including menstrual hygiene management (MHM) (*n* = 6, 3.5%), and abortion (*n* = 6, 4%) were identified much less frequently in this review. A lack of coverage and infrequent use of indicators and data on the share of meals within the household (women’s prioritization) was also evident (*n* = 3, 2%). Survivorship by gender was only measured in 1 study (0.6%). The majority of health indicators were found in the peer-reviewed literature.

#### Human development

Learning/educational outcomes were not captured by our review, with the exception of literacy, which was found in 5 studies (3%). Access to telecommunications appeared infrequently in our scoping review (*n* = 1, 0.6%).

Access to trainings/consultations was found in 17 studies (10%), 5 (3%) of which were peer-reviewed publications. Access to basic needs/essential infrastructure was present in 17 (10%) studies, only three (2%) of which were peer-reviewed literature. Indicators that measure empowerment as an outcome (change/effect) from an intervention appeared in only 6 studies, 2 of which were peer-reviewed. Even when pooling all the empowerment subtypes, it appeared only in 10% of the studies (*n* = 17).

#### Leadership

There was a clear gap in leadership studies in our review, as only 15 studies (9%) mentioned this domain. The majority of the leadership indicators were related to community engagement (*n* = 14, 8%), with only six studies (4%) including indicators about women’s leadership, none of which were peer-reviewed publications.

#### Psychological

The psychological domain was identified in 26 studies included in this review (15%). While there were some studies with psychological indicators related to social networks (*n* = 19, 11%), fewer studies (*n* = 10, 6%) had indicators related to self-esteem. More specifically, the indicator subtype feelings/outlook about the future was only found in two studies. All of these types/subtypes were uncommon in the peer-reviewed literature.

#### Security and justice

Some aspects of security and justice were identified in multiple studies and include abduction (*n* = 20, 12%), locations of violence (*n* = 14, 8%) and perpetrator information (*n* = 49, 29%). However, systems-related topics, including those pertaining to laws that protect women (*n* = 3, 2%), legal violence (*n* = 1, 0.6%), and access to the justice system (*n* = 13, 8%) were infrequently measured, were found predominately in the non-peer-reviewed literature. Since measuring legal violence predicates on measuring access to the legal system, it is logical that the coverage of this indicator is accordingly low.

#### Sociocultural

The sociocultural domain was the largest domain in this review (*n* = 137, 81%). GBV was the most frequent indicator in this domain and in the review overall (*n* = 113, 66%). GBV sub-types such as sexual violence, physical violence, and intimate partner violence (IPV) were measured in 65 (38%), 41 (24%) and 45 (26%) studies respectively. GBV attitudes were present in 33 (19%) studies.

Our review captured important measures of GEWE such as women’s autonomy (*n* = 29, 17%) and gender equitable attitudes (*n* = 25, 15%). Indicator types related to marriage, such as age of first marriage (including child marriage), forced marriage and marriage norms were found in 25, 16, and 19 studies (15, 10, 11%) respectively. Indicators for *acceptance of individuals who identify as lesbian, gay, bisexual, transgender and/or queer (LGBTQ+)* (*n* = 2, 1%) and *awareness of individuals who identify as LGBTQ+ community member*s (*n* = 1, 0.6%) were not often measured or included in the toolkits assessed.

Despite the substantial number of studies using indicators pertaining to the sociocultural domain, there were several gaps in indicator types within this domain. Early pregnancy (*n* = 6, 4%), female genital mutilation/cutting (FGM/C) and other traditional practices (*n* = 11, 6%), and rights and awareness of LGBTQ+ (*n* = 4, 2%) were lacking in measurement.

Though indicators of early pregnancy were included in toolkits, they were not commonly captured in our review, and appeared only in the non-peer-reviewed literature (*n* = 6, 4%). Early pregnancy is defined as a pregnancy in an adolescent girl aged 19 or younger [23]. Adolescent girls constitute 22% (*n* = 38) of the study populations included in this review (see study characteristics), making this a notable gap in measurement in the sociocultural domain. Only one publication reported the indicator *proportion of girls who reported they had ever been pregnant* [24]. Further, perceptions of early pregnancy were only measured in one non peer-reviewed literature study [25].

## Discussion

### Discussion of measurement methods used

Few interventional studies were identified in this review even though these are particularly important for capturing the effect of interventions that focus on empowerment, as a measure of changes in the degree of autonomy and/or self-determination in women through various interventions or programs. In addition, the lack of a control group for participants unexposed to an intervention or humanitarian event limits the interpretation of program/intervention effects [26, 27].

The majority of the studies in this review were cross-sectional surveys. A random sample is a subset of the population in which each member has an equal probability of being chosen. Random sampling is meant to be unbiased, as it is an approximate to what would be obtained if the entire population was sampled [28]. Random sampling is the best method to use for cross-sectional surveys, as it allows the results to be generalizable to the population [29]. In the context of health in humanitarian settings, it allows for a more accurate measure of the indicator across the population. However, probability sampling may not be feasible in every humanitarian setting. Violence, displacement, and insecurity can make sampling difficult [30]. Additionally, poor infrastructure can limit research capacity, compromising the validity of the research findings [30]. These challenges may result in the use of non-probability sampling methods [31]. In our review, studies used non-probability sampling, limiting their ability to make causal claims and to generalize their findings to the broader population [32–35]. For example, studies that only recruit women seeking care in primary health settings may lead to selection bias [36–38].

Interviewer-administered and self-administered questionnaires can be subject to social desirability bias, as participants may be reluctant to disclose personal information, or may want to appeal to the perceived views and opinions of the interviewer [25, 32, 39–41]. Retrospective questionnaires are also prone to recall bias [42].

### Economic

Economic initiatives are often touted as a means to women’s empowerment. Multiple developmental programs and research initiatives have focused on women’s economic empowerment in humanitarian settings [43–45]. The lack of measurement of women’s control of their own earnings is a significant gap in the measurement of economic empowerment, as it represents the lack of women’s agency [8].

Additionally, measures of women’s economic decision-making may be lacking because conflict-settings provided minimal opportunities for women’s leadership overall, thereby limiting opportunities for women’s economic leadership. Overall, women’s *involvement* in economic decision-making was captured by indicators found in this review. However, the degree of involvement varied; *shared economic decision-making* often did not mean *equal* decision-making capacity for men and women. Therefore, future measurements of women’s economic decision-making and women’s allocation of spending must capture this nuance.

Economic support can be through formal or informal means. Formal economic support (FES) refers to the formal financial institutions that offer structured loans with quality assurance mechanisms in place, which often have high economic and opportunity costs for those in LMICs. Informal economic support (IES) includes friends, family, and rotating savings schemes, which are more accessible by those living in humanitarian settings [46]. For the purposes of this review, economic interventions/development programs are grouped with FES. Only 5 % of borrowers in countries with humanitarian crises borrowed from a formal financial institution [46]. Therefore, measurement of IES should not be limited to the non-peer-reviewed literature as it serves as a useful resource for women in crisis-settings when FESs are limited. Loss of income and poverty may contribute to the prevalence of transactional sex [47, 48]. Transactional sex is considered a risky sexual behavior [49] and may also be associated with experiences of sexual violence, which would explain its prevalence within our included studies.

### Health

The health in the gender equality and women’s empowerment literature lacks sex-disaggregated data as many of the indicators are female-specific. The lack of sex-disaggregation in the health domain is not necessarily an issue, as indicators related to abortion, pregnancy, birth and menstruation predominately impact those who identify as women, and do not apply to most men. Health-related indicators are the focus of the SRHR scoping review published elsewhere [12]. Inequalities in household meal-allocation between men and women in humanitarian settings, are not well understood due a gap in measurement of this indicator. This is a concern, as adult women, even those who are pregnant, may be given last priority for meals in times of scarcity.

### Human development

Human development was more often identified in non-peer-reviewed literature reports than in the peer-reviewed literature. Indicators of educational/learning outcomes were not frequently identified in this review. Educational interventions in humanitarian settings may not be evaluated using traditional outcomes such as literacy and numeracy; evaluating gender-disaggregated attendance or participation may be prioritized over academic achievement alone [50, 51]. Further, in humanitarian settings, education might not be delivered in a traditional format nor focused on traditional topics with well-established measurement standards, including numeracy and literacy rates [51]. In humanitarian settings, schooling may be replaced by training, or programming that focuses on SRH knowledge [39] or business/vocational skills [43, 52]. Indicators related to education in humanitarian settings include those by the Inter-agency Network for Education in Emergencies [51]. These indicators were not retrieved in our review.

The lack of sex-disaggregation related to education, empowerment and access to services represents a gap in the literature. Sex-disaggregation is important when evaluating indicators of gender equality and empowerment, especially those related to access to education and services. In order to fully understand what is needed to promote equality and empowerment, an indicator must show how women fare compared to men or to the general population. The identification of existing inequalities through sex-disaggregation helps ensure that future policies and interventions are gender responsive [7].

Telecommunication access is a SDG sex-disaggregated indicator [1]. Empowerment interventions in conflict-settings that focus on the utility of mobile technology, such as in delivering early warning systems, and alerting users to the availability of aid, may help to bridge this gap [53]. A case study from two refugee contexts in East Africa [54] found that women were less likely than men to use a mobile phone or mobile internet, and disproportionately relied on borrowing phones from others. The study also found that women lacked the digital and literacy skills necessary to work various features of mobile phones [54].

In our scoping review, indicators of empowerment as a measure of change/effect resulting from an intervention, are classified as *empowerment indicators*. These were defined as a quantitative difference in attitudes, knowledge, or income, post-intervention and were rarely reported in the reviewed studies. Agency, which is a different conceptualization of empowerment that is not a consequence of an intervention, but rather a trait that defines the individual as an agent of change for their own development, and the development of the community around them [13] is classified in our scoping review under the psychological and leadership domains, respectively.

### Leadership

Leadership was the domain with the lowest number of identified indicators. This lack of leadership measurement could in part be due to the scope of this review, as it did not capture national-level data. For example, the SDG indicator, *proportion of seats held by women in national parliaments and local governments*, was not captured for this reason. Nevertheless, there is a gap in the measurement of community-level leadership in conflict and disaster settings.

Leadership interventions may not be prioritized in humanitarian settings, especially in the immediate aftermath of a humanitarian event, where the health and security needs are prioritized. With the assistance of leadership interventions, women’s leadership can grow in refugee communities [55]. Women and girls can contribute to crisis responses, strengthening social cohesion and preventing conflict between displaced and host communities. Women should be engaged as leaders and policymakers in risk reduction, to help ensure that gender specific needs are met in these settings. Women are also disproportionately impacted by natural disasters and play an important role in community-level resilience [55]. The lived experiences of women are crucial to governance in humanitarian settings, thus indicators of women’s involvement in this kind of leadership are necessary. It is also important to measure both men and women’s attitudes towards women’s leadership [56].

### Psychological

As mentioned above, indicators within the psychological domain were uncommon in this review. Self-esteem is a measure of women’s empowerment, and has been included in women’s empowerment frameworks around the world [57]. Improved self-esteem can increase self-efficacy, self-worth and sense of belonging in a community [57, 58]. Therefore, indicators measuring psychological well-being should be better captured in humanitarian settings.

### Security and justice

Locations of violence and perpetrators of violence appeared across multiple studies, countries and in the peer-reviewed literature, due to the high prevalence of gender-based violence measures.

Access to the justice system, laws and perception of safety were not common in the peer-reviewed literature. This may indicate that qualitative methods have been used to collect information on these topics, or it might reflect the perception that legal governance/the justice system is too far removed from the experiences of women in humanitarian settings. However, for holistic change, it may be necessary to advocate for these robust systems of justice to ensure that women’s rights are being protected [59] and measuring these indicators might bridge this gap.

### Sociocultural

The predominance of GBV indicators in this review may be a reflection of the prevalence of violence in crisis-affected settings. Violence against women and girls (VAWG) can be used as a weapon of war, and a lack of security in camps and informal settlements can allow for an increased perpetration of violence [33, 60–67]. Stress caused by disaster, political instability and loss of income can increase relationship tensions, leading to increased IPV [68–71]. However, this concept is based on the psychobiology of stress theory of IPV, and is not the sole explanation for IPV [72]. Different theories exist to attempt to explain IPV, and there is intersectionality between these theories [73]. The depth of GBV research in both the peer-reviewed and non-peer-reviewed literature illustrate that the severity of GBV in humanitarian settings is recognized by non-governmental organizations (NGOs) and governments.

The prevalence of early/child marriage captured in this review may be explained by the economic and physical insecurity caused by conflict and displacement. Parents may believe it can alleviate the economic burden of providing food or clothing to their female children. Child marriage may also be a misguided attempted to “protect” girls from sexual violence in informal settlements [74].

Women’s autonomy and gender equitable attitudes were measured throughout this review. It is important to measure changes in gender roles at the household and community level after the onset of a humanitarian event, as women become increasingly more vulnerable to violence, lack of access to essential services, loss of income and an increase in domestic/family responsibilities [53, 75, 76]. It is also to measure men’s attitudes alongside women’s, which was why the gender equitable men’s scale was used in multiple studies. It measures attitudes towards gender roles within the home [48, 56, 75–77].

Not all indicator types within the sociocultural domain were commonly identified. One example is early pregnancy, which impacts girls’ school enrolment and attendance [24], thereby curtailing their access to education and opportunities for employment. Therefore, future empowerment projects among adolescent girls in conflict-affected settings would benefit from the use of agreed-upon measures of early pregnancy.

As highlighted in our results, FGM/C was not commonly identified in this review. The elimination of all harmful practices, including FGM/C, is a target outlined by SDG five [1]. Programs and/or interventions for FGM/C and other harmful practices, and VAWG are often executed independently from one another [78]. However, these programs and/or interventions may benefit from coordinated implementation and evaluation [78]. To fully understand violence against women and girls, it is necessary to consistently collect data on comparable indicators of FGM/C specifically, so that it is not masked by more general violence indicators [78]. It is also important to measure the prevalence and attitudes towards FGM/C to address these harmful practices and achieve gender equality across the various domains of women’s lives [1]. Methodologies used to capture VAWG may also be used to develop and implement FGM/C evaluations [78] so that more detailed, standardized, and global evidence of this practice is available.

Notably, LGBTQ+ acceptance and awareness constitute a significant gap in this review. This may be because homosexuality is criminalized in some of these settings, and/or speaking about LGBTQ+ issues is taboo in the cultural context [56, 77]. Future measurement initiatives require culturally sensitive approaches to capture the LGBTQ+ experience in conflict/humanitarian settings.

### Limitations

Indicators were only extracted if they reported quantitative outcomes, thereby excluding toolkits and checklists that were retrieved directly in our non-peer-reviewed literature search and were not used in any of the included publications. We excluded qualitative studies; therefore, it is possible that a number of in-depth studies related to equality and empowerment were excluded. Second, only studies written or translated into the English language were included due to our limited capacity to translate, thus excluding papers written in local languages. Third, this study did not include nationwide data, rendering our results less comparable to existing national indicators of empowerment and equality. We could not compare our indicators to the majority of the indicators that fall under SGD five, including the proportion of parliamentary seats held by women. Fourth, this review was heteronormative, as the majority of studies equated the term women to biological female sex, and not gender identity. There was little to no inclusion of transgender and non-binary people. Fifth, we only captured the interventions that have been evaluated and documented, not the entirety of what is being implemented for GEWE in conflict and other humanitarian settings. The lack of peer-reviewed publications on certain domains of equality and empowerment may reflect the challenges faced by NGOs working in humanitarian crises to publish in peer-reviewed journals and not a dearth of data. Thus, there may be fewer interventional *studies* in humanitarian settings, but not necessarily a lack of available interventions. Sixth, standardized age classification would have been preferable, as it increases the comparability of indicators across settings. We had to create our own age classifications, and many studies fell into multiple categories. Seventh, the distinctions between our subtypes were often subtle, and in some cases contentious, as some indicators like empowerment fall into multiple domains. Finally, for this review, unlike in our SRHR review, we did not compare our findings with a specific framework or standard indicators. There are less validated indicators and methodologies for measuring GEWE as compared to SRHR, rendering comparison challenging.

## Conclusions

While there has been some effort to measure GEWE in humanitarian settings, there are significant gaps. A range of sampling methods were used in this review, yet the vast majority of studies employed an observational design. The lack of interventional studies found in this review demonstrated that humanitarian organizations should prioritize publishing literature on women’s empowerment interventions, whilst using reliable measurement methods. The interactive Tableau dashboard illustrates the gaps, methods and indicators found in this review, and can be updated based on future GEWE research.

Violence and security indicators were commonly identified in this review, while human development, leadership and economic empowerment indicators were lacking. Some of these gaps can be attributed to the lack of a comprehensive framework for measuring GEWE in humanitarian settings. Though challenging to develop, this measurement framework could streamline data collection, and increase the comparability of indicators across diverse settings. With the onset of the COVID-19 pandemic, women and girls are becoming increasingly vulnerable to gendered impacts of conflicts, disasters, and epidemics. Therefore, it becomes even more imperative to have reliable, validated tools and indicators to measure GEWE in humanitarian settings.

## Supplementary Information

**Additional file 1.** Search strategy. This file includes the search strategy for all 6 databases.

**Additional file 2.** Inclusion and exclusion criteria. This file includes the inclusion and exclusion criteria for the review.

**Additional file 3.** Machine learning tool. This file explains the machine learning tool we used to conduct the title/abstract screening phase of the review.

**Additional file 4.** Data collection toolkits and surveys. This file explains the data collection tools used by studies included in this review.

**Additional file 5.** Measurement of Sexual and Reproductive Health and Rights, Gender Equality, and Women’s Empowerment in Humanitarian Settings. This link leads to the interactive Tableau dashboard. https://public.tableau.com/profile/humairanakhuda#!/vizhome/SRHRGEWEScopingReviewStory_Final_Nov16/SRHRGEWEStory.

**Additional file 6.** Included studies. This file contains all of the included studies retrieved in both the peer-reviewed and non-peer-reviewed literature searches.

**Additional file 7.** Studies by disaster type. This file contains a list of all publications by disaster type.

**Additional file 8.** Indicator types and subtypes. This file contains the calculated frequency of each indicator type and subtype organized by domain.

## Data Availability

The datasets generated during and/or analyzed during the current study are available from the corresponding author on reasonable request.

## Abbreviations

ACASI: Audio Computer-Assisted Self-Interviews
ALNAP: Active Learning Network for Accountability and Performance
CanWaCH: Canadian Partnership for Women and Children’s Health
CARE: Care International
DHS: Demographic and Health Survey
FES: Formal Economic Support (FES)
FGM/C: Female Genital Mutilation/Cutting
FP: Family Planning
GBV: Gender-Based Violence
GEWE: Gender Equality and Women’s Empowerment
IAWG: Inter-Agency Working Group on Reproductive Health in Crises
ICRC: International Committee of the Red Cross
ICRW: International Center for Research on Women
IDP: Internally Displaced Persons
IES: Informal Economic Support (IES)
IPV: Intimate Partner Violence
IRC: International Rescue Committee
LGBTQ+: Lesbian, gay, bisexual, transgender and queer or questioning
LMICs: Low- and Middle- Income Countries
MHM: Menstrual Hygiene Management
MSF: Médecins Sans Frontières
NGOs: Non-Governmental Organizations
PRISMA: Preferred Reporting Items for Systematic Reviews
SCI: Save the Children International
SDGs: Sustainable Development Goals
SRHR: Sexual and Reproductive Health and Rights
SVRI: Sexual Violence Research Initiatives
UNDP: United Nations Development Programme
UNFPA: United Nations Population Fund
UNHCR: United Nations High Commission for Refugees
VAWG: Violence Against Women and Girls
WHO: World Health Organization
WRA: Women of Reproductive Age
