# Supplementary material for: Tools for measuring gender equality and women’s empowerment (GEWE) indicators in humanitarian settings
Source: Confl Health. 2021 May 17;15:39. doi: 10.1186/s13031-021-00373-6 (PMC8127307; doi:10.1186/s13031-021-00373-6)
Supplement: Supplementary file 1 — Additional file 1. Search strategy. This file includes the search strategy for all 6 databases. [file 13031_2021_373_MOESM1_ESM.pdf]

Additional file 1: Search Strategy

Database(s): CINAHL

Search Strategy:

Wednesday, November 27, 2019 2:34:40 PM

#	Query	Limiters/Expanders	Last Run Via	Results
S128	S122 AND S123 AND S124 AND S125 AND S126	Limiters - Published Date: 20040101-20191231<br>Expanders - Apply equivalent subjects<br>Search modes - Boolean/Phrase	Interface - EBSCOhost Research Databases<br>Search Screen - Advanced Search<br>Database - CINAHL Plus with Full Text	6,739
S127	S122 AND S123 AND S124 AND S125 AND S126	Expanders - Apply equivalent subjects<br>Search modes - Boolean/Phrase	Interface - EBSCOhost Research Databases<br>Search Screen - Advanced Search<br>Database - CINAHL Plus with Full Text	7,251
S126	S78 OR S79 OR S80 OR S81 OR S82 OR S83 OR S84 OR S85 OR S86 OR S87 OR S88 OR S89 OR S90 OR S91 OR S92 OR S93 OR S94 OR S95 OR S96 OR S97 OR S98 OR S99 OR S100 OR S101 OR S102 OR S103 OR S104 OR S105 OR S106 OR S107 OR S108 OR S109 OR S110 OR S111 OR S112 OR S113 OR S114 OR S115 OR S116 OR S117 OR S118 OR S119 OR S120 OR S121	Expanders - Apply equivalent subjects<br>Search modes - Boolean/Phrase	Interface - EBSCOhost Research Databases<br>Search Screen - Advanced Search<br>Database - CINAHL Plus with Full Text	1,033,396
S125	S58 OR S59 OR S60 OR S61 OR S62 OR S63 OR S64 OR S65 OR S66 OR S67 OR S68 OR S69 OR S70 OR S71 OR S72 OR S73 OR S74 OR S75 OR S76 OR S77	Expanders - Apply equivalent subjects<br>Search modes - Boolean/Phrase	Interface - EBSCOhost Research Databases<br>Search Screen - Advanced Search<br>Database - CINAHL Plus with Full Text	2,750,043
S124	S45 OR S46 OR S47 OR S48 OR S49 OR S50 OR	Expanders - Apply equivalent subjects	Interface - EBSCOhost Research Databases	492,560

	S51 OR S52 OR S53 OR S54 OR S55 OR S56 OR S57	Search modes - Boolean/Phrase	Search Screen - Advanced Search<br>Database - CINAHL Plus with Full Text	
S123	S38 OR S39 OR S40 OR S41 OR S42 OR S43 OR S44	Expanders - Apply equivalent subjects<br>Search modes - Boolean/Phrase	Interface - EBSCOhost Research Databases<br>Search Screen - Advanced Search<br>Database - CINAHL Plus with Full Text	2,060,698
S122	S1 OR S2 OR S3 OR S4 OR S5 OR S6 OR S7 OR S8 OR S9 OR S10 OR S11 OR S12 OR S13 OR S14 OR S15 OR S16 OR S17 OR S18 OR S19 OR S20 OR S21 OR S22 OR S23 OR S24 OR S25 OR S26 OR S27 OR S28 OR S29 OR S30 OR S31 OR S32 OR S33 OR S34 OR S35 OR S36 OR S37	Expanders - Apply equivalent subjects<br>Search modes - Boolean/Phrase	Interface - EBSCOhost Research Databases<br>Search Screen - Advanced Search<br>Database - CINAHL Plus with Full Text	172,903
S121	(MH "Maternal-Child Health") OR (MH "Reproductive Health") OR (MH "Women's Health")	Expanders - Apply equivalent subjects<br>Search modes - Boolean/Phrase	Interface - EBSCOhost Research Databases<br>Search Screen - Advanced Search<br>Database - CINAHL Plus with Full Text	Display
S120	(MH "Health Care Delivery") OR (MH "Health Services Accessibility") OR (MH "Healthcare Disparities") OR (MH "Quality of Health Care")	Expanders - Apply equivalent subjects<br>Search modes - Boolean/Phrase	Interface - EBSCOhost Research Databases<br>Search Screen - Advanced Search<br>Database - CINAHL Plus with Full Text	Display
S119	AB (decision making N3 (power or joint or domestic or intrahousehold or "intra household" or influence)) OR TI (decision making N3 (power or joint or domestic or	Expanders - Apply equivalent subjects<br>Search modes - Boolean/Phrase	Interface - EBSCOhost Research Databases<br>Search Screen - Advanced Search<br>Database - CINAHL Plus with Full Text	Display

	intrahousehold or "intra household" or influence))			
S118	(MH "Socioeconomic Factors") OR (MH "Economic Status") OR (MH "Educational Status") OR (MH "Income") OR (MH "Health Status Disparities") OR (MH "Poverty")	Expanders - Apply equivalent subjects<br>Search modes - Boolean/Phrase	Interface - EBSCOhost Research Databases<br>Search Screen - Advanced Search<br>Database - CINAHL Plus with Full Text	Display
S117	AB ((wom?n or women's or female) N5 ("decision making" or decisionmaking)) OR TI ((wom?n or women's or female) N5 ("decision making" or decision-making))	Expanders - Apply equivalent subjects<br>Search modes - Boolean/Phrase	Interface - EBSCOhost Research Databases<br>Search Screen - Advanced Search<br>Database - CINAHL Plus with Full Text	Display
S116	AB ((gender or wom?n or women's or female or girl) N3 (disempower* or autonomy or participation or status or preference# or power or agency or right# or feminism or authorit* or control* or leadership or choice or voice or "critical consciousness" or working or mobility or freedom or ownership or assets or (contribution N2 family income) or "reproductive rights" or movement or training or "labo#r participation" or "self esteem" or "collective action*" or "self-efficacy" or "self efficacy" or "skill# building" or "self respect" or "self worth" or "solidarity")) OR TI ((gender or wom?n or	Expanders - Apply equivalent subjects<br>Search modes - Boolean/Phrase	Interface - EBSCOhost Research Databases<br>Search Screen - Advanced Search<br>Database - CINAHL Plus with Full Text	Display

	women's or female or girl) N3 (disempower* or autonomy or participation or status or preference# or power or agency or right# or feminism or authorit* or control* or leadership or choice or voice or "critical consciousness" or working or mobility or freedom or ownership or assets or (contribution N2 family income) or "reproductive rights" or movement or training or "labo#r participation" or "self esteem" or "collective action*" or "self-efficacy" or "self efficacy" or "skill# building" or "self respect" or "self worth" or "solidarity"))			
S115	AB ((gender or wom?n or women's or female or girl) adj3 (disempower* or autonomy or participation or status or preference# or power or agency or right# or feminism or authorit* or control* or leadership or choice or voice or "critical consciousness" or working or mobility or freedom or ownership or assets or (contribution N2 family income) or "reproductive rights" or movement or training or "labo#r participation" or "self esteem" or "collective action*" or "self-efficacy" or "self efficacy" or "skill#	Expanders - Apply equivalent subjects<br>Search modes - Boolean/Phrase	Interface - EBSCOhost Research Databases<br>Search Screen - Advanced Search<br>Database - CINAHL Plus with Full Text	Display

	building" or "self respect" or "self worth" or "solidarity"))			
S114	AB ((gender or wom?n or women's or female or girl) adj3 (disempower* or autonomy or participation or status or preference# or power or agency or right# or feminism or authorit* or control* or leadership or choice or voice or "critical consciousness" or working or mobility or freedom or ownership or assets or (contribution N2 family income) or "reproductive rights" or movement or training or "labo#r participation" or "self esteem" or "collective action*" or "self-efficacy" or "self efficacy" or "skill# building" or "self respect" or "self worth" or "solidarity"))	Expanders - Apply equivalent subjects<br>Search modes - Boolean/Phrase	Interface - EBSCOhost Research Databases<br>Search Screen - Advanced Search<br>Database - CINAHL Plus with Full Text	Display
S113	AB ((gender or wom?n or women's or female or girl) adj3 (disempower* or autonomy or participation or status or preference# or power or agency or right# or feminism or authorit* or control* or leadership or choice or voice or "critical consciousness" or working or mobility or freedom or ownership or assets or (contribution N2 family income) or "reproductive rights" or movement or training or	Expanders - Apply equivalent subjects<br>Search modes - Boolean/Phrase	Interface - EBSCOhost Research Databases<br>Search Screen - Advanced Search<br>Database - CINAHL Plus with Full Text	Display

	"labo#r participation" or "self esteem" or "collective action*" or "self-efficacy" or "self efficacy" or "skill# building" or "self respect" or "self worth" or "solidarity"))			
S112	(MH "Decision Making")	Expanders - Apply equivalent subjects<br>Search modes - Boolean/Phrase	Interface - EBSCOhost Research Databases<br>Search Screen - Advanced Search<br>Database - CINAHL Plus with Full Text	Display
S111	AB ((wom?n or women's or girl# or female) N5 ("safe space#" or "friendly space#")) OR TI ((wom?n or women's or girl# or female) N5 ("safe space#" or "friendly space#"))	Expanders - Apply equivalent subjects<br>Search modes - Boolean/Phrase	Interface - EBSCOhost Research Databases<br>Search Screen - Advanced Search<br>Database - CINAHL Plus with Full Text	Display
S110	AB ("safe space#" or "female friendly space#") OR TI ("safe space#" or "female friendly space#")	Expanders - Apply equivalent subjects<br>Search modes - Boolean/Phrase	Interface - EBSCOhost Research Databases<br>Search Screen - Advanced Search<br>Database - CINAHL Plus with Full Text	Display
S109	AB (empower* N3 (social or economic or health or legal or financial or political or psychological)) OR TI (empower* N3 (social or economic or health or legal or financial or political or psychological))	Expanders - Apply equivalent subjects<br>Search modes - Boolean/Phrase	Interface - EBSCOhost Research Databases<br>Search Screen - Advanced Search<br>Database - CINAHL Plus with Full Text	Display
S108	AB ((wom?n or women's or female or girl) N3 (empower*)) OR TI ((wom?n or women's or female or girl) N3 (empower*))	Expanders - Apply equivalent subjects<br>Search modes - Boolean/Phrase	Interface - EBSCOhost Research Databases<br>Search Screen - Advanced Search<br>Database - CINAHL Plus with Full Text	Display
S107	(MH "Autonomy") OR (MH	Expanders - Apply	Interface - EBSCOhost	Display

	"Self-Efficacy") OR (MH "Self Concept")	equivalent subjects Search modes - Boolean/Phrase	Research Databases Search Screen - Advanced Search Database - CINAHL Plus with Full Text	
S106	AB (Attitude# N5 (violence or "wife beating" or "refusing sex")) OR TI (Attitude# N5 (violence or "wife beating" or "refusing sex"))	Expanders - Apply equivalent subjects Search modes - Boolean/Phrase	Interface - EBSCOhost Research Databases Search Screen - Advanced Search Database - CINAHL Plus with Full Text	Display
S105	(MH "Attitude") OR (MH "Attitude to Health") OR (MH "Health Behavior") OR (MH "Help Seeking Behavior") OR (MH "Support Groups")	Expanders - Apply equivalent subjects Search modes - Boolean/Phrase	Interface - EBSCOhost Research Databases Search Screen - Advanced Search Database - CINAHL Plus with Full Text	Display
S104	AB ((gender or wom?n or women's or feminine* or female* or reproductive) N3 (victim* or abus* or "sex* crime" or "rape" or "assault" or "sexual harassment" or "sexual coercion" or "forced sex" or "sexual slavery" or “sex work" or battered or "sexual exploitation" or "human trafficking" or "restricted physical integrity" or patriarch* or "bodily integrity")) OR TI ((gender or wom?n or women's or feminine* or female* or reproductive) N3 (victim* or abus* or "sex* crime" or "rape" or "assault" or "sexual harassment" or "sexual coercion" or "forced sex" or "sexual slavery" or “sex work" or battered or "sexual exploitation" or "human trafficking" or	Expanders - Apply equivalent subjects Search modes - Boolean/Phrase	Interface - EBSCOhost Research Databases Search Screen - Advanced Search Database - CINAHL Plus with Full Text	Display

	"restricted physical integrity" or patriarch* or "bodily integrity"))			
S103	(MH "Battered Women")	Expanders - Apply equivalent subjects<br>Search modes - Boolean/Phrase	Interface - EBSCOhost Research Databases<br>Search Screen - Advanced Search<br>Database - CINAHL Plus with Full Text	Display
S102	AB (gender N3 (relat* or specific or responsive or prescription* or dimension*)) OR TI (gender N3 (relat* or specific or responsive or prescription* or dimension*))	Expanders - Apply equivalent subjects<br>Search modes - Boolean/Phrase	Interface - EBSCOhost Research Databases<br>Search Screen - Advanced Search<br>Database - CINAHL Plus with Full Text	Display
S101	AB (gender N3 traditional) OR TI (gender N3 traditional)	Expanders - Apply equivalent subjects<br>Search modes - Boolean/Phrase	Interface - EBSCOhost Research Databases<br>Search Screen - Advanced Search<br>Database - CINAHL Plus with Full Text	Display
S100	AB (gender N3 (sociali?ation or identity)) OR TI (gender N3 (sociali?ation or identity))	Expanders - Apply equivalent subjects<br>Search modes - Boolean/Phrase	Interface - EBSCOhost Research Databases<br>Search Screen - Advanced Search<br>Database - CINAHL Plus with Full Text	Display
S99	(MH "Socialization")	Expanders - Apply equivalent subjects<br>Search modes - Boolean/Phrase	Interface - EBSCOhost Research Databases<br>Search Screen - Advanced Search<br>Database - CINAHL Plus with Full Text	Display
S98	AB ((gender or wom?n or women's or feminine* or female* or reproductive) N3 (difference# or stereotype# or perspective# or intersect* or sensiti?ation)) OR TI ((gender or wom?n or	Expanders - Apply equivalent subjects<br>Search modes - Boolean/Phrase	Interface - EBSCOhost Research Databases<br>Search Screen - Advanced Search<br>Database - CINAHL Plus with Full Text	Display

	women's or feminine* or female* or reproductive) N3 (difference# or stereotype# or perspective# or intersect* or sensiti?ation))			
S97	AB ((gender or wom?n or women's or feminine* or female* or reproductive) N3 (transformat*)) OR TI ((gender or wom?n or women's or feminine* or female* or reproductive) N3 (transformat*))	Expanders - Apply equivalent subjects<br>Search modes - Boolean/Phrase	Interface - EBSCOhost Research Databases<br>Search Screen - Advanced Search<br>Database - CINAHL Plus with Full Text	Display
S96	AB ((gender or wom?n or women's or feminine* or female* or reproductive) N3 (role# or norm# or dynamic# or resilience)) OR TI ((gender or wom?n or women's or feminine* or female* or reproductive) N3 (role# or norm# or dynamic# or resilience))	Expanders - Apply equivalent subjects<br>Search modes - Boolean/Phrase	Interface - EBSCOhost Research Databases<br>Search Screen - Advanced Search<br>Database - CINAHL Plus with Full Text	Display
S95	(MH "Social Norms") OR (MH "Sex Factors")	Expanders - Apply equivalent subjects<br>Search modes - Boolean/Phrase	Interface - EBSCOhost Research Databases<br>Search Screen - Advanced Search<br>Database - CINAHL Plus with Full Text	Display
S94	AB ((gender or wom?n or women's or feminine* or female* or reproductive) N3 (discriminat* or bias* or blind* or gap# or barrier#)) OR TI ((gender or wom?n or women's or feminine* or female* or reproductive) N3 (discriminat* or bias* or blind* or gap# or barrier#))	Expanders - Apply equivalent subjects<br>Search modes - Boolean/Phrase	Interface - EBSCOhost Research Databases<br>Search Screen - Advanced Search<br>Database - CINAHL Plus with Full Text	Display
S93	AB ((gender or wom?n or	Expanders - Apply	Interface - EBSCOhost	Display

	women's or feminine* or female* or reproductive) N3 (equal* or inequality* or unequal* or justice* or injustice* or equit* or inequit* or disparit* or parit*)) OR TI ((gender or wom?n or women's or feminine* or female* or reproductive) N3 (equal* or inequality* or unequal* or justice* or injustice* or equit* or inequit* or disparit* or parit*))	equivalent subjects<br>Search modes - Boolean/Phrase	Research Databases<br>Search Screen - Advanced Search<br>Database - CINAHL Plus with Full Text	
S92	AB ((gender or wom?n or women's or feminine* or female* or reproductive) N3 (politic* or "civil libert*" or (access N2 public space) or "parliamentary seats")) OR TI ((gender or wom?n or women's or feminine* or female* or reproductive) N3 (politic* or "civil libert*" or (access N2 public space) or "parliamentary seats"))	Expanders - Apply equivalent subjects<br>Search modes - Boolean/Phrase	Interface - EBSCOhost Research Databases<br>Search Screen - Advanced Search<br>Database - CINAHL Plus with Full Text	Display
S91	(MH "Politics") OR (MH "Political Participation")	Expanders - Apply equivalent subjects<br>Search modes - Boolean/Phrase	Interface - EBSCOhost Research Databases<br>Search Screen - Advanced Search<br>Database - CINAHL Plus with Full Text	Display
S90	(MH "Marriage")	Expanders - Apply equivalent subjects<br>Search modes - Boolean/Phrase	Interface - EBSCOhost Research Databases<br>Search Screen - Advanced Search<br>Database - CINAHL Plus with Full Text	Display
S89	AB ((gender or wom?n or women's or feminine* or female* or reproductive) N3 (security or land or inheritance or marriage or	Expanders - Apply equivalent subjects<br>Search modes - Boolean/Phrase	Interface - EBSCOhost Research Databases<br>Search Screen - Advanced Search	Display

	"early marriage" or "parental authority")) OR TI ((gender or wom?n or women's or feminine* or female* or reproductive) N3 (security or land or inheritance or marriage or "early marriage" or "parental authority"))		Database - CINAHL Plus with Full Text	
S88	AB ((gender or wom?n or women's or feminine* or female* or reproductive) N3 ("life expectancy" or "fertility rate" or "birth rate" or "son preference" or (sex ratio N2 birth) or (fertility N3 preference*))) OR TI ((gender or wom?n or women's or feminine* or female* or reproductive) N3 ("life expectancy" or "fertility rate" or "birth rate" or "son preference" or (sex ratio N2 birth) or (fertility N3 preference*)))	Expanders - Apply equivalent subjects<br>Search modes - Boolean/Phrase	Interface - EBSCOhost Research Databases<br>Search Screen - Advanced Search<br>Database - CINAHL Plus with Full Text	Display
S87	(MH "Birth Rate") OR (MH "Life Expectancy")	Expanders - Apply equivalent subjects<br>Search modes - Boolean/Phrase	Interface - EBSCOhost Research Databases<br>Search Screen - Advanced Search<br>Database - CINAHL Plus with Full Text	Display
S86	AB ((gender or wom?n or women's or feminine* or female* or reproductive) N3 (wage# or employ* or "labo#r force" or "pay gap" or underemploy* or unemploy* or microfinance or microcredit or "occupational segregation" or economic or poverty or "basic	Expanders - Apply equivalent subjects<br>Search modes - Boolean/Phrase	Interface - EBSCOhost Research Databases<br>Search Screen - Advanced Search<br>Database - CINAHL Plus with Full Text	Display

	needs" or microfinance or microcredit or financial or (cash N2 transfer#) or voucher# or (saving# N2 group#) or (self help N2 group#) or (self-help N2 group#) or income)) OR TI ((gender or wom?n or women's or feminine* or female* or reproductive) N3 (wage# or employ* or "labo#r force" or "pay gap" or underemploy* or unemploy* or microfinance or microcredit or "occupational segregation" or economic or poverty or "basic needs" or microfinance or microcredit or financial or (cash N2 transfer#) or voucher# or (saving# N2 group#) or (self help N2 group#) or (self-help N2 group#) or income))			
S85	(MH "Unemployment") OR (MH "Occupations and Professions") OR (MH "Employment")	Expanders - Apply equivalent subjects<br>Search modes - Boolean/Phrase	Interface - EBSCOhost Research Databases<br>Search Screen - Advanced Search<br>Database - CINAHL Plus with Full Text	Display
S84	AB ((gender or wom?n or women's or feminine* or female* or reproductive) N3 (educat* or "school enrollment" or "school attendance" or literacy or (access N2 information))) OR TI ((gender or wom?n or women's or feminine* or female* or reproductive) N3 (educat* or "school enrollment" or "school attendance" or	Expanders - Apply equivalent subjects<br>Search modes - Boolean/Phrase	Interface - EBSCOhost Research Databases<br>Search Screen - Advanced Search<br>Database - CINAHL Plus with Full Text	Display

	literacy or (access N2 information)))			
S83	(MH "Education") OR (MH "Access to Information") OR (MH "Literacy")	Expanders - Apply equivalent subjects<br>Search modes - Boolean/Phrase	Interface - EBSCOhost Research Databases<br>Search Screen - Advanced Search<br>Database - CINAHL Plus with Full Text	Display
S82	AB ((gender or wom?n or women's or feminine* or female* or reproductive) N3 (violence)) female* or reproductive) N3 (violence)) OR TI ((gender or wom?n or women's or feminine* or	Expanders - Apply equivalent subjects<br>Search modes - Boolean/Phrase	Interface - EBSCOhost Research Databases<br>Search Screen - Advanced Search<br>Database - CINAHL Plus with Full Text	Display
S81	(MH "Domestic Violence") OR (MH "Gender-Based Violence") OR (MH "Intimate Partner Violence") OR (MH "Human Trafficking") OR (MH "Sexual Abuse") OR (MH "Rape") OR (MH "Child Abuse, Sexual")	Expanders - Apply equivalent subjects<br>Search modes - Boolean/Phrase	Interface - EBSCOhost Research Databases<br>Search Screen - Advanced Search<br>Database - CINAHL Plus with Full Text	Display
S80	(MH "Interpersonal Relations") OR (MH "Empowerment") OR (MH "Power")	Expanders - Apply equivalent subjects<br>Search modes - Boolean/Phrase	Interface - EBSCOhost Research Databases<br>Search Screen - Advanced Search<br>Database - CINAHL Plus with Full Text	Display
S79	(MH "Women's Rights") OR (MH "Human Rights") OR (MH "Feminism")	Expanders - Apply equivalent subjects<br>Search modes - Boolean/Phrase	Interface - EBSCOhost Research Databases<br>Search Screen - Advanced Search<br>Database - CINAHL Plus with Full Text	Display
S78	(MH "Gender Identity") OR (MH "Gender Bias") OR (MH "Gender Role")	Expanders - Apply equivalent subjects<br>Search modes - Boolean/Phrase	Interface - EBSCOhost Research Databases<br>Search Screen - Advanced Search<br>Database - CINAHL Plus with Full Text	Display

S77	AB ((Africa gender N2 development index) or "gender status index" or "African women's progress scoreboard" or "millennium development goals" or "sustainable development goals" or "gender mainstreaming scorecard" or "gender equality action") OR TI ((Africa gender N2 development index) or "gender status index" or "African women's progress scoreboard" or "millennium development goals" or "sustainable development goals" or "gender mainstreaming scorecard" or "gender equality action")	Expanders - Apply equivalent subjects<br>Search modes - Boolean/Phrase	Interface - EBSCOhost Research Databases<br>Search Screen - Advanced Search<br>Database - CINAHL Plus with Full Text	Display
S76	AB ((social institutions N2 gender index) or (Survey N2 women's empowerment index) or SWPER or (women's empowerment N2 agriculture index) or WEAI or (demographic N2 health surveys)) OR TI ((social institutions N2 gender index) or (Survey N2 women's empowerment index) or SWPER or (women's empowerment N2 agriculture index) or WEAI or (demographic N2 health surveys))	Expanders - Apply equivalent subjects<br>Search modes - Boolean/Phrase	Interface - EBSCOhost Research Databases<br>Search Screen - Advanced Search<br>Database - CINAHL Plus with Full Text	Display
S75	AB ("gender empowerment measure" or "gender related development index" or "gender development	Expanders - Apply equivalent subjects<br>Search modes - Boolean/Phrase	Interface - EBSCOhost Research Databases<br>Search Screen - Advanced Search	Display

	index" or "gender gap index" or global gender gap index" or global gender gap report" or "gender intensity measure" or "gender inequality index" or "gender equity index" or "gender parity index" or "human development index") OR TI ("gender empowerment measure" or "gender related development index" or "gender development index" or "gender gap index" or global gender gap index" or global gender gap report" or "gender intensity measure" or "gender inequality index" or "gender equity index" or "gender parity index" or "human development index")		Database - CINAHL Plus with Full Text	
S74	AB (gender N3 (analys* or analy?ing or mainstreaming or marker or "risk assessment") OR TI (gender N3 (analys* or analy?ing or mainstreaming or marker or "risk assessment")	Expanders - Apply equivalent subjects<br>Search modes - Boolean/Phrase	Interface - EBSCOhost Research Databases<br>Search Screen - Advanced Search<br>Database - CINAHL Plus with Full Text	Display
S73	(MH "Outcome Assessment")	Expanders - Apply equivalent subjects<br>Search modes - Boolean/Phrase	Interface - EBSCOhost Research Databases<br>Search Screen - Advanced Search<br>Database - CINAHL Plus with Full Text	Display
S72	(MH "Health Services Research")	Expanders - Apply equivalent subjects<br>Search modes - Boolean/Phrase	Interface - EBSCOhost Research Databases<br>Search Screen - Advanced Search	Display

			Database - CINAHL Plus with Full Text	
S71	AB (outcome# N3 (health or measur* or assess* or (score or scoring) or index or indices or scale# or monitor#)) OR TI ((outcome# N3 (health or measur* or assess* or (score or scoring) or index or indices or scale# or monitor#))	Expanders - Apply equivalent subjects<br>Search modes - Boolean/Phrase	Interface - EBSCOhost Research Databases<br>Search Screen - Advanced Search<br>Database - CINAHL Plus with Full Text	Display
S70	AB (measure* or metric* or method*) OR TI (measure* or metric* or method*)	Expanders - Apply equivalent subjects<br>Search modes - Boolean/Phrase	Interface - EBSCOhost Research Databases<br>Search Screen - Advanced Search<br>Database - CINAHL Plus with Full Text	Display
S69	AB ("minimum initial service package" or MISP) OR TI ("minimum initial service package" or MISP)	Expanders - Apply equivalent subjects<br>Search modes - Boolean/Phrase	Interface - EBSCOhost Research Databases<br>Search Screen - Advanced Search<br>Database - CINAHL Plus with Full Text	Display
S68	AB (index or indices) OR TI (index or indices)	Expanders - Apply equivalent subjects<br>Search modes - Boolean/Phrase	Interface - EBSCOhost Research Databases<br>Search Screen - Advanced Search<br>Database - CINAHL Plus with Full Text	Display
S67	AB (scale# or survey# or questionnaire# or measured or measurement or metric#) OR TI (scale# or survey# or questionnaire# or measured or measurement or metric#)	Expanders - Apply equivalent subjects<br>Search modes - Boolean/Phrase	Interface - EBSCOhost Research Databases<br>Search Screen - Advanced Search<br>Database - CINAHL Plus with Full Text	Display
S66	AB ("data collection tool*" or "data source*" or "data collection") OR TI ("data collection tool*" or "data	Expanders - Apply equivalent subjects<br>Search modes - Boolean/Phrase	Interface - EBSCOhost Research Databases<br>Search Screen - Advanced Search	Display

	source*" or "data collection")		Database - CINAHL Plus with Full Text	
S65	(MH "Questionnaires+") OR (MH "Research Instruments+") OR (MH "Instrument by Type+") OR (MH "Research Measurement+") or (MH "surveys")	Expanders - Apply equivalent subjects Search modes - Boolean/Phrase	Interface - EBSCOhost Research Databases Search Screen - Advanced Search Database - CINAHL Plus with Full Text	Display
S64	(MH "Data Collection+") OR (MH "Data Collection Methods+") OR (MH "Study Design+")	Expanders - Apply equivalent subjects Search modes - Boolean/Phrase	Interface - EBSCOhost Research Databases Search Screen - Advanced Search Database - CINAHL Plus with Full Text	Display
S63	AB ((rapid or needs) N2 (assessment# or evaluation#)) OR TI ((rapid or needs) N2 (assessment# or evaluation#))	Expanders - Apply equivalent subjects Search modes - Boolean/Phrase	Interface - EBSCOhost Research Databases Search Screen - Advanced Search Database - CINAHL Plus with Full Text	Display
S62	(MH "Needs Assessment") OR (MH "Clinical Assessment Tools+") OR (MH "Clinical Indicators") OR (MH "Outcome Assessment") OR (MH "Process Assessment (Health Care)") OR (MH "Risk Assessment")	Expanders - Apply equivalent subjects Search modes - Boolean/Phrase	Interface - EBSCOhost Research Databases Search Screen - Advanced Search Database - CINAHL Plus with Full Text	Display
S61	AB ("rapid counting" or "aerial surveillance" or "flow monitoring" or "enumeration" or (reproductive health N3 assessment toolkit)) OR TI ("rapid counting" or "aerial surveillance" or "flow monitoring" or "enumeration" or (reproductive health N3 assessment toolkit))	Expanders - Apply equivalent subjects Search modes - Boolean/Phrase	Interface - EBSCOhost Research Databases Search Screen - Advanced Search Database - CINAHL Plus with Full Text	Display

S60	AB (monitor* or surveillance or screening) OR TI (monitor* or surveillance or screening)	Expanders - Apply equivalent subjects<br>Search modes - Boolean/Phrase	Interface - EBSCOhost Research Databases<br>Search Screen - Advanced Search<br>Database - CINAHL Plus with Full Text	Display
S59	AB (indicator# or "health indicator#" or evaluation or tool# or methodolog* or standards) OR TI (indicator# or "health indicator#" or evaluation or tool# or methodolog* or standards)	Expanders - Apply equivalent subjects<br>Search modes - Boolean/Phrase	Interface - EBSCOhost Research Databases<br>Search Screen - Advanced Search<br>Database - CINAHL Plus with Full Text	Display
S58	MH (Health status indicators)	Expanders - Apply equivalent subjects<br>Search modes - Boolean/Phrase	Interface - EBSCOhost Research Databases<br>Search Screen - Advanced Search<br>Database - CINAHL Plus with Full Text	Display
S57	AB (transitional countr*) OR TI (transitional countr*)	Expanders - Apply equivalent subjects<br>Search modes - Boolean/Phrase	Interface - EBSCOhost Research Databases<br>Search Screen - Advanced Search<br>Database - CINAHL Plus with Full Text	Display
S56	AB (lmic or lmics or third world or lami countr*) OR TI (lmic or lmics or third world or lami countr*)	Expanders - Apply equivalent subjects<br>Search modes - Boolean/Phrase	Interface - EBSCOhost Research Databases<br>Search Screen - Advanced Search<br>Database - CINAHL Plus with Full Text	Display
S55	AB (low N3 middle N3 (countr* or nation*)) OR TI (low N3 middle N3 (countr* or nation*))	Expanders - Apply equivalent subjects<br>Search modes - Boolean/Phrase	Interface - EBSCOhost Research Databases<br>Search Screen - Advanced Search<br>Database - CINAHL Plus with Full Text	Display
S54	AB (low* N3 (gdp or gnp or gross domestic or gross national)) OR TI (low* N3 (gdp or gnp or	Expanders - Apply equivalent subjects<br>Search modes - Boolean/Phrase	Interface - EBSCOhost Research Databases<br>Search Screen - Advanced Search	Display

	gross domestic or gross national))		Database - CINAHL Plus with Full Text	
S53	AB ((developing or less* developed or under developed or underdeveloped or middle income or low* income) N3 (economy or economies)) OR TI ((developing or less* developed or under developed or underdeveloped or middle income or low* income) N3 (economy or economies))	Expanders - Apply equivalent subjects<br>Search modes - Boolean/Phrase	Interface - EBSCOhost Research Databases<br>Search Screen - Advanced Search<br>Database - CINAHL Plus with Full Text	Display
S52	AB ((developing or "less* developed" or "under developed" or underdeveloped or "middle income" or "low* income" or underserved or "under served" or deprived or poor*) N2 (countr* or nation? or population? or world)) OR TI ((developing or "less* developed" or "under developed" or underdeveloped or "middle income" or "low* income" or underserved or "under served" or deprived or poor*) N2 (countr* or nation? or population? or world))	Expanders - Apply equivalent subjects<br>Search modes - Boolean/Phrase	Interface - EBSCOhost Research Databases<br>Search Screen - Advanced Search<br>Database - CINAHL Plus with Full Text	Display
S51	(MH "Africa+") OR (MH "Africa South of the Sahara+") OR (MH "Africa, Northern+") OR (MH "Africa, Western+") OR (MH "Africa, Southern+") OR (MH "Africa, Eastern+") OR	Expanders - Apply equivalent subjects<br>Search modes - Boolean/Phrase	Interface - EBSCOhost Research Databases<br>Search Screen - Advanced Search<br>Database - CINAHL Plus with Full Text	Display

	(MH "Africa, Central+") OR (MH "Central America+") OR (MH "South America+") OR (MH "West Indies+") OR (MH "Asia+") OR (MH "Middle East+")			
S50	AB (Afghanistan or Albania or Algeria or American Samoa or Angola or Armenia or Armenian or Azerbaijan or Bangladesh or Benin or Byelarus or Byelorussian or Belarus or Belorussian or Belorussia or Belize or Bhutan or Bolivia or Bosnia or Herzegovina or Hercegovina or Botswana or Brasil or Brazil or Bulgaria or Burkina Faso or Burkina Fasso or Upper Volta or Burundi or Urundi or Cambodia or Khmer Republic or Kampuchea or Cameroon or Cameroons or Cameron or Camerons or Cameroun or Cape Verde or Cabo Verde or Central African Republic or Chad or China or Colombia or Comoros or Comoro Islands or Comores or Mayotte or Congo or Zaire or Costa Rica or Cote d'Ivoire or Ivory Coast or Cuba or Djibouti or French Somaliland or Dominica or Dominican Republic or East Timor or East Timur or Timor Leste or Ecuador or Egypt or United Arab Republic or El Salvador or Equatorial Guinea or Eritrea or Ethiopia or Fiji	Expanders - Apply equivalent subjects<br>Search modes - Boolean/Phrase	Interface - EBSCOhost Research Databases<br>Search Screen - Advanced Search<br>Database - CINAHL Plus with Full Text	Display

or Gabon or Gabonese
Republic or Gambia or
Gaza or Georgia or
Georgia Republic or
Georgian Republic or
Ghana or Gold Coast or
Grenada or Guatemala or
Guinea or Bissau or
Guiana or Guyana or Haiti
or Honduras or India or
Maldives or Indonesia or
Iran or Iraq or Jamaica or
Jordan or Kazakhstan or
Kazakh or Kenya or
Kiribati or Korea or
Democratic People's
Republic of Korea or
Kosovo or Kyrgyzstan or
Kirghizia or Kyrgyz
Republic or Kirghiz or
Kirgizstan or Lao PDR or
Laos or Lebanon or
Lesotho or Basutoland or
Liberia or Libya or
Macedonia or
Madagascar or Malagasy
Republic or Malaysia or
Malaya or Malay or Sabah
or Sarawak or Malawi or
Nyasaland or Mali or
Marshall Islands or
Mauritania or Mauritius or
Agalega Islands or Mexico
or Micronesia or Middle
East or Moldova or
Moldovia or Moldovian or
Mongolia or Montenegro
or Morocco or Ifni or
Mozambique or Myanmar
or Myanma or Burma or
Namibia or Nauru or
Nepal or Nicaragua or
Niger or Nigeria or
Pakistan or Papua New
Guinea or Palestine or
Paraguay or Peru or

Philippines or Philipines or
Phillipines or Phillippines
or Romania or Rumania
or Roumania or Russia or
Russian or Rwanda or
Ruanda or Saint Lucia or
St Lucia or Saint Vincent
or St Vincent or
Grenadines or Samoa or
Samoan Islands or
Navigator Island or
Navigator Islands or Sao
Tome or Senegal or
Serbia or Montenegro or
Sierra Leone or Sri Lanka
or Ceylon or Solomon
Islands or Somalia or
South Africa or Sudan or
South Sudan or Suriname
or Surinam or Swaziland
or Eswatini or Syria or
Syrian Arab Republic or
Tajikistan or Tadzhikistan
or Tadjikistan or Tadzhik
or Tanzania or Thailand or
Togo or Togolese
Republic or Tonga or
Tunisia or Turkey or
Turkmenistan or Turkmen
or Tuvalu or Uganda or
Ukraine or USSR or
Soviet Union or Union of
Soviet Socialist Republics
or Uzbekistan or Uzbek or
Vanuatu or New Hebrides
or Venezuela or Vietnam
or Viet Nam or West Bank
or Yemen or Yugoslavia or
Zambia or Zimbabwe or
Rhodesia) OR TI
(Afghanistan or Albania or
Algeria or American
Samoa or Angola or
Armenia or Armenian or
Azerbaijan or Bangladesh
or Benin or Byelarus or

Byelorussian or Belarus or
Belorussian or Belorussia
or Belize or Bhutan or
Bolivia or Bosnia or
Herzegovina or
Hercegovina or Botswana
or Brasil or Brazil or
Bulgaria or Burkina Faso
or Burkina Fasso or Upper
Volta or Burundi or Urundi
or Cambodia or Khmer
Republic or Kampuchea
or Cameroon or
Cameroons or Cameron
or Camerons or
Cameroun or Cape Verde
or Cabo Verde or Central
African Republic or Chad
or China or Colombia or
Comoros or Comoro
Islands or Comores or
Mayotte or Congo or Zaire
or Costa Rica or Cote
d'Ivoire or Ivory Coast or
Cuba or Djibouti or French
Somaliland or Dominica or
Dominican Republic or
East Timor or East Timur
or Timor Leste or Ecuador
or Egypt or United Arab
Republic or El Salvador or
Equatorial Guinea or
Eritrea or Ethiopia or Fiji
or Gabon or Gabonese
Republic or Gambia or
Gaza or Georgia or
Georgia Republic or
Georgian Republic or
Ghana or Gold Coast or
Grenada or Guatemala or
Guinea or Bissau or
Guiana or Guyana or Haiti
or Honduras or India or
Maldives or Indonesia or
Iran or Iraq or Jamaica or
Jordan or Kazakhstan or

Kazakh or Kenya or
Kiribati or Korea or
Democratic People's
Republic of Korea or
Kosovo or Kyrgyzstan or
Kirghizia or Kyrgyz
Republic or Kirghiz or
Kirgizstan or Lao PDR or
Laos or Lebanon or
Lesotho or Basutoland or
Liberia or Libya or
Macedonia or
Madagascar or Malagasy
Republic or Malaysia or
Malaya or Malay or Sabah
or Sarawak or Malawi or
Nyasaland or Mali or
Marshall Islands or
Mauritania or Mauritius or
Agalega Islands or Mexico
or Micronesia or Middle
East or Moldova or
Moldovia or Moldovian or
Mongolia or Montenegro
or Morocco or Ifni or
Mozambique or Myanmar
or Myanma or Burma or
Namibia or Nauru or
Nepal or Nicaragua or
Niger or Nigeria or
Pakistan or Papua New
Guinea or Palestine or
Paraguay or Peru or
Philippines or Philipines or
Phillipines or Phillippines
or Romania or Rumania
or Roumania or Russia or
Russian or Rwanda or
Ruanda or Saint Lucia or
St Lucia or Saint Vincent
or St Vincent or
Grenadines or Samoa or
Samoan Islands or
Navigator Island or
Navigator Islands or Sao
Tome or Senegal or

	Serbia or Montenegro or Sierra Leone or Sri Lanka or Ceylon or Solomon Islands or Somalia or South Africa or Sudan or South Sudan or Suriname or Surinam or Swaziland or Eswatini or Syria or Syrian Arab Republic or Tajikistan or Tadzhikistan or Tadjikistan or Tadzhik or Tanzania or Thailand or Togo or Togolese Republic or Tonga or Tunisia or Turkey or Turkmenistan or Turkmen or Tuvalu or Uganda or Ukraine or USSR or Soviet Union or Union of Soviet Socialist Republics or Uzbekistan or Uzbek or Vanuatu or New Hebrides or Venezuela or Vietnam or Viet Nam or West Bank or Yemen or Yugoslavia or Zambia or Zimbabwe or Rhodesia)			
S49	MH (Afghanistan or Albania or Algeria or American Samoa or Angola or Armenia or Armenian or Azerbaijan or Bangladesh or Benin or Byelarus or Byelorussian or Belarus or Belorussian or Belorussia or Belize or Bhutan or Bolivia or Bosnia or Herzegovina or Hercegovina or Botswana or Brasil or Brazil or Bulgaria or Burkina Faso or Burkina Fasso or Upper Volta or Burundi or Urundi or Cambodia or Khmer Republic or Kampuchea or Cameroon or	Expanders - Apply equivalent subjects<br>Search modes - Boolean/Phrase	Interface - EBSCOhost Research Databases<br>Search Screen - Advanced Search<br>Database - CINAHL Plus with Full Text	Display

Cameroons or Cameron
or Camerons or
Cameroun or Cape Verde
or Cabo Verde or Central
African Republic or Chad
or China or Colombia or
Comoros or Comoro
Islands or Comores or
Mayotte or Congo or Zaire
or Costa Rica or Cote
d'Ivoire or Ivory Coast or
Cuba or Djibouti or French
Somaliland or Dominica or
Dominican Republic or
East Timor or East Timur
or Timor Leste or Ecuador
or Egypt or United Arab
Republic or El Salvador or
Equatorial Guinea or
Eritrea or Ethiopia or Fiji
or Gabon or Gabonese
Republic or Gambia or
Gaza or Georgia or
Georgia Republic or
Georgian Republic or
Ghana or Gold Coast or
Grenada or Guatemala or
Guinea or Bissau or
Guiana or Guyana or Haiti
or Honduras or India or
Maldives or Indonesia or
Iran or Iraq or Jamaica or
Jordan or Kazakhstan or
Kazakh or Kenya or
Kiribati or Korea or
Democratic People's
Republic of Korea or
Kosovo or Kyrgyzstan or
Kirghizia or Kyrgyz
Republic or Kirghiz or
Kirgizstan or Lao PDR or
Laos or Lebanon or
Lesotho or Basutoland or
Liberia or Libya or
Macedonia or
Madagascar or Malagasy

Republic or Malaysia or
Malaya or Malay or Sabah
or Sarawak or Malawi or
Nyasaland or Mali or
Marshall Islands or
Mauritania or Mauritius or
Agalega Islands or Mexico
or Micronesia or Middle
East or Moldova or
Moldovia or Moldovian or
Mongolia or Montenegro
or Morocco or Ifni or
Mozambique or Myanmar
or Myanma or Burma or
Namibia or Nauru or
Nepal or Nicaragua or
Niger or Nigeria or
Pakistan or Papua New
Guinea or Palestine or
Paraguay or Peru or
Philippines or Philipines or
Phillipines or Phillippines
or Romania or Rumania
or Roumania or Russia or
Russian or Rwanda or
Ruanda or Saint Lucia or
St Lucia or Saint Vincent
or St Vincent or
Grenadines or Samoa or
Samoan Islands or
Navigator Island or
Navigator Islands or Sao
Tome or Senegal or
Serbia or Montenegro or
Sierra Leone or Sri Lanka
or Ceylon or Solomon
Islands or Somalia or
South Africa or Sudan or
South Sudan or Suriname
or Surinam or Swaziland
or Eswatini or Syria or
Syrian Arab Republic or
Tajikistan or Tadzhikistan
or Tadjikistan or Tadzhik
or Tanzania or Thailand or
Togo or Togolese

	Republic or Tonga or Tunisia or Turkey or Turkmenistan or Turkmen or Tuvalu or Uganda or Ukraine or USSR or Soviet Union or Union of Soviet Socialist Republics or Uzbekistan or Uzbek or Vanuatu or New Hebrides or Venezuela or Vietnam or Viet Nam or West Bank or Yemen or Yugoslavia or Zambia or Zimbabwe or Rhodesia)			
S48	AB (Africa or Asia or West Indies or South America or Latin America or Central America) OR TI (Africa or Asia or West Indies or South America or Latin America or Central America)	Expanders - Apply equivalent subjects<br>Search modes - Boolean/Phrase	Interface - EBSCOhost Research Databases<br>Search Screen - Advanced Search<br>Database - CINAHL Plus with Full Text	Display
S47	(MH "Africa") OR (MH "South America") OR (MH "Latin America") OR (MH "West Indies") OR (MH "Central America") OR (MH "Asia")	Expanders - Apply equivalent subjects<br>Search modes - Boolean/Phrase	Interface - EBSCOhost Research Databases<br>Search Screen - Advanced Search<br>Database - CINAHL Plus with Full Text	Display
S46	AB (Developing Countries) OR TI (Developing Countries)	Expanders - Apply equivalent subjects<br>Search modes - Boolean/Phrase	Interface - EBSCOhost Research Databases<br>Search Screen - Advanced Search<br>Database - CINAHL Plus with Full Text	Display
S45	(MH "Low and Middle Income Countries") OR (MH "Developing Countries")	Expanders - Apply equivalent subjects<br>Search modes - Boolean/Phrase	Interface - EBSCOhost Research Databases<br>Search Screen - Advanced Search<br>Database - CINAHL Plus with Full Text	Display
S44	AB (women N2 reproductive N2 age) OR	Expanders - Apply equivalent subjects	Interface - EBSCOhost Research Databases<br>Search Screen - Advanced	Display

	TI (women N2 reproductive N2 age)	Search modes - Boolean/Phrase	Search<br>Database - CINAHL Plus with Full Text	
S43	(MH "Female")	Expanders - Apply equivalent subjects<br>Search modes - Boolean/Phrase	Interface - EBSCOhost Research Databases<br>Search Screen - Advanced Search<br>Database - CINAHL Plus with Full Text	Display
S42	AB (mother# or female# or wom?n) OR TI (mother# or female# or wom?n)	Expanders - Apply equivalent subjects<br>Search modes - Boolean/Phrase	Interface - EBSCOhost Research Databases<br>Search Screen - Advanced Search<br>Database - CINAHL Plus with Full Text	Display
S41	(MH "Women")	Expanders - Apply equivalent subjects<br>Search modes - Boolean/Phrase	Interface - EBSCOhost Research Databases<br>Search Screen - Advanced Search<br>Database - CINAHL Plus with Full Text	Display
S40	(MH "Expectant Mothers") OR (MH "Expectant Parents") OR (MH "Mothers") OR (MH "Adolescent Mothers")	Expanders - Apply equivalent subjects<br>Search modes - Boolean/Phrase	Interface - EBSCOhost Research Databases<br>Search Screen - Advanced Search<br>Database - CINAHL Plus with Full Text	Display
S39	AB (adolescent# or teen* or youth# or "young adult#" or girl# or "young wom?n" or "young girl#") OR TI (adolescent# or teen* or youth# or "young adult#" or girl# or "young wom?n" or "young girl#")	Expanders - Apply equivalent subjects<br>Search modes - Boolean/Phrase	Interface - EBSCOhost Research Databases<br>Search Screen - Advanced Search<br>Database - CINAHL Plus with Full Text	Display
S38	(MH "Young Adult") OR (MH "Adolescence")	Expanders - Apply equivalent subjects<br>Search modes - Boolean/Phrase	Interface - EBSCOhost Research Databases<br>Search Screen - Advanced Search<br>Database - CINAHL Plus with Full Text	Display
S37	AB (settlement# N3	Expanders - Apply	Interface - EBSCOhost	Display

	(temporary or informal)) OR TI (settlement# N3 (temporary or informal))	equivalent subjects Search modes - Boolean/Phrase	Research Databases Search Screen - Advanced Search Database - CINAHL Plus with Full Text	
S36	AB ((camp or camps) N3 (refugee or transit or displace* or temporary or informal)) OR TI ((camp or camps) N3 (refugee or transit or displace* or temporary or informal))	Expanders - Apply equivalent subjects Search modes - Boolean/Phrase	Interface - EBSCOhost Research Databases Search Screen - Advanced Search Database - CINAHL Plus with Full Text	Display
S35	(MH "Refugee Camps")	Expanders - Apply equivalent subjects Search modes - Boolean/Phrase	Interface - EBSCOhost Research Databases Search Screen - Advanced Search Database - CINAHL Plus with Full Text	Display
S34	AB ("displaced child*" or "displaced famil*" or "displaced individual#" or "displaced internally" or "displaced men" or "displaced people#" or "displaced person#" or "displaced population#" or "displaced wom?n" or "displaced adolescent#" or "forced displacement" or "forcibl* displace*" or "internal* displace*" or "population displace*" or "forced migration" or "migrant#") OR TI ("displaced child*" or "displaced famil*" or "displaced individual#" or "displaced internally" or "displaced men" or "displaced people#" or "displaced person#" or "displaced population#" or "displaced wom?n" or "displaced adolescent#"	Expanders - Apply equivalent subjects Search modes - Boolean/Phrase	Interface - EBSCOhost Research Databases Search Screen - Advanced Search Database - CINAHL Plus with Full Text	Display

	or "forced displacement" or "forcibl* displace*" or "internal* displace*" or "population displace*" or "forced migration" or "migrant#")			
S33	AB (humanitarianism or altruism) OR TI (humanitarianism or altruism)	Expanders - Apply equivalent subjects<br>Search modes - Boolean/Phrase	Interface - EBSCOhost Research Databases<br>Search Screen - Advanced Search<br>Database - CINAHL Plus with Full Text	Display
S32	(MH "Altruism")	Expanders - Apply equivalent subjects<br>Search modes - Boolean/Phrase	Interface - EBSCOhost Research Databases<br>Search Screen - Advanced Search<br>Database - CINAHL Plus with Full Text	Display
S31	AB (humanitarian N5 (aid or response or relief or crisis or crises or emergency or emergencies or disaster or disasters)) OR TI (humanitarian N3 (aid or response or relief or crisis or crises or emergency or emergencies or disaster or disasters))	Expanders - Apply equivalent subjects<br>Search modes - Boolean/Phrase	Interface - EBSCOhost Research Databases<br>Search Screen - Advanced Search<br>Database - CINAHL Plus with Full Text	Display
S30	AB (humanitarian assist*) OR TI (humanitarian assist*)	Expanders - Apply equivalent subjects<br>Search modes - Boolean/Phrase	Interface - EBSCOhost Research Databases<br>Search Screen - Advanced Search<br>Database - CINAHL Plus with Full Text	Display
S29	AB ((staff* or worker#) N3 (relief or aid or rescue)) OR TI ((staff* or worker#) N3 (relief or aid or rescue))	Expanders - Apply equivalent subjects<br>Search modes - Boolean/Phrase	Interface - EBSCOhost Research Databases<br>Search Screen - Advanced Search<br>Database - CINAHL Plus with Full Text	Display
S28	AB ((rescue or relief or aid) N3 (work or plan or	Expanders - Apply equivalent subjects	Interface - EBSCOhost Research Databases	Display

	plans or activity or activities or agency or agencies)) OR TI ((rescue or relief or aid) N3 (work or plan or plans or activity or activities or agency or agencies))	Search modes - Boolean/Phrase	Search Screen - Advanced Search<br>Database - CINAHL Plus with Full Text	
S27	(MH "Humanitarian Aid") OR (MH "Rescue Work")	Expanders - Apply equivalent subjects<br>Search modes - Boolean/Phrase	Interface - EBSCOhost Research Databases<br>Search Screen - Advanced Search<br>Database - CINAHL Plus with Full Text	Display
S26	AB (evacuee# or refugee# or squatter# or transient# or "asylum seeker#") OR TI (evacuee# or refugee# or squatter# or transient# or "asylum seeker#")	Expanders - Apply equivalent subjects<br>Search modes - Boolean/Phrase	Interface - EBSCOhost Research Databases<br>Search Screen - Advanced Search<br>Database - CINAHL Plus with Full Text	Display
S25	(MH "Refugees")	Expanders - Apply equivalent subjects<br>Search modes - Boolean/Phrase	Interface - EBSCOhost Research Databases<br>Search Screen - Advanced Search<br>Database - CINAHL Plus with Full Text	Display
S24	AB (avalanche# or cyclone# or drought# or earthquake# or flood* or hurricane# or landslide# or "land slide#" or mudslide# or "mud slide#" or storm# or tornado* or tsunami# or typhoon# or volcanic or rubble) OR TI (avalanche# or cyclone# or drought# or earthquake# or flood* or hurricane# or landslide# or "land slide#" or mudslide# or "mud slide#" or storm# or tornado* or tsunami# or typhoon# or volcanic or rubble)	Expanders - Apply equivalent subjects<br>Search modes - Boolean/Phrase	Interface - EBSCOhost Research Databases<br>Search Screen - Advanced Search<br>Database - CINAHL Plus with Full Text	Display

S23	AB (famine# or starvation#) OR TI (famine# or starvation#)	Expanders - Apply equivalent subjects<br>Search modes - Boolean/Phrase	Interface - EBSCOhost Research Databases<br>Search Screen - Advanced Search<br>Database - CINAHL Plus with Full Text	Display
S22	(MH "Starvation")	Expanders - Apply equivalent subjects<br>Search modes - Boolean/Phrase	Interface - EBSCOhost Research Databases<br>Search Screen - Advanced Search<br>Database - CINAHL Plus with Full Text	Display
S21	AB ((emergency or emergencies) N5 (environ* or human or manmade or "man made" or nature or natural or weather or complex)) OR TI ((emergency or emergencies) N5 (environ* or human or manmade or "man made" or nature or natural or weather or complex))	Expanders - Apply equivalent subjects<br>Search modes - Boolean/Phrase	Interface - EBSCOhost Research Databases<br>Search Screen - Advanced Search<br>Database - CINAHL Plus with Full Text	Display
S20	(MH "Emergency Medical Services")	Expanders - Apply equivalent subjects<br>Search modes - Boolean/Phrase	Interface - EBSCOhost Research Databases<br>Search Screen - Advanced Search<br>Database - CINAHL Plus with Full Text	Display
S19	AB ("disaster medicine" or "disaster outbreak*" or epidemic* or "disease outbreak*") OR TI ("disaster medicine" or "disaster outbreak*" or epidemic* or "disease outbreak*")	Expanders - Apply equivalent subjects<br>Search modes - Boolean/Phrase	Interface - EBSCOhost Research Databases<br>Search Screen - Advanced Search<br>Database - CINAHL Plus with Full Text	Display
S18	(MH "Disease Outbreaks")	Expanders - Apply equivalent subjects<br>Search modes - Boolean/Phrase	Interface - EBSCOhost Research Databases<br>Search Screen - Advanced Search	Display

			Database - CINAHL Plus with Full Text	
S17	AB ("militant group#" or "militant organi?ation#" or militia or combatant or rebel*) OR TI ("militant group#" or "militant organi?ation#" or militia or combatant or rebel*)	Expanders - Apply equivalent subjects<br>Search modes - Boolean/Phrase	Interface - EBSCOhost Research Databases<br>Search Screen - Advanced Search<br>Database - CINAHL Plus with Full Text	Display
S16	AB (war N3 related) OR TI (war N3 related)	Expanders - Apply equivalent subjects<br>Search modes - Boolean/Phrase	Interface - EBSCOhost Research Databases<br>Search Screen - Advanced Search<br>Database - CINAHL Plus with Full Text	Display
S15	AB ("Post conflict" or "postconflict" or "post war" or peacebuilding or peacekeeping) OR TI ("Post conflict" or "postconflict" or "post war" or peacebuilding or peacekeeping)	Expanders - Apply equivalent subjects<br>Search modes - Boolean/Phrase	Interface - EBSCOhost Research Databases<br>Search Screen - Advanced Search<br>Database - CINAHL Plus with Full Text	Display
S14	AB (fragile N2 (state* or countr* or nation* or situation* or setting*)) OR TI (fragile N2 (state* or countr* or nation* or situation* or setting*))	Expanders - Apply equivalent subjects<br>Search modes - Boolean/Phrase	Interface - EBSCOhost Research Databases<br>Search Screen - Advanced Search<br>Database - CINAHL Plus with Full Text	Display
S13	AB ((armed or zone or political or civil or setting*) N3 (conflict# or attack# or war# or "no fly")) OR TI ((armed or zone or political or civil or setting*) N3 (conflict# or attack# or war# or "no fly"))	Expanders - Apply equivalent subjects<br>Search modes - Boolean/Phrase	Interface - EBSCOhost Research Databases<br>Search Screen - Advanced Search<br>Database - CINAHL Plus with Full Text	Display
S12	AB (“afghan campaign” or “gulf war” or “iraq war” or “war time” or “wartime” or “war torn” or “war affected” or “insurgency” or “intra conflict”) OR TI	Expanders - Apply equivalent subjects<br>Search modes - Boolean/Phrase	Interface - EBSCOhost Research Databases<br>Search Screen - Advanced Search<br>Database - CINAHL Plus with Full Text	Display

	("afghan campaign" or "gulf war" or "iraq war" or "war time" or "wartime" or "war torn" or "war affected" or "insurgency" or "intra conflict")			
S11	AB ("warfare and armed conflict*" or warfare or "armed conflict*" or "war crime*" or "ethnic cleansing*" or "gas poisoning" or genocide or "war exposure") OR TI ("warfare and armed conflict*" or warfare or "armed conflict*" or "war crime*" or "ethnic cleansing*" or "gas poisoning" or genocide or "war exposure")	Expanders - Apply equivalent subjects<br>Search modes - Boolean/Phrase	Interface - EBSCOhost Research Databases<br>Search Screen - Advanced Search<br>Database - CINAHL Plus with Full Text	Display
S10	(MH "Violence") OR (MH "Exposure to Violence")	Expanders - Apply equivalent subjects<br>Search modes - Boolean/Phrase	Interface - EBSCOhost Research Databases<br>Search Screen - Advanced Search<br>Database - CINAHL Plus with Full Text	Display
S9	(MM "War+") OR (MM "War Crimes+")	Expanders - Apply equivalent subjects<br>Search modes - Boolean/Phrase	Interface - EBSCOhost Research Databases<br>Search Screen - Advanced Search<br>Database - CINAHL Plus with Full Text	Display
S8	AB ((crisis or crises or conflict) N3 (affected)) OR TI ((crisis or crises or conflict) N3 (affected))	Expanders - Apply equivalent subjects<br>Search modes - Boolean/Phrase	Interface - EBSCOhost Research Databases<br>Search Screen - Advanced Search<br>Database - CINAHL Plus with Full Text	Display
S7	AB ((crisis or crises) N5 (environment* or human* or manmade or "man made" or nature or natural or weather or setting*))	Expanders - Apply equivalent subjects<br>Search modes - Boolean/Phrase	Interface - EBSCOhost Research Databases<br>Search Screen - Advanced Search	Display

	OR TI ((crisis or crises) N5 (environment* or human* or manmade or "man made" or nature or natural or weather or setting*))		Database - CINAHL Plus with Full Text	
S6	AB ("mass casualty" or "mass casualties" or "mass fatalities" or "mass fatality") OR TI ("mass casualty" or "mass casualties" or "mass fatalities" or "mass fatality")	Expanders - Apply equivalent subjects<br>Search modes - Boolean/Phrase	Interface - EBSCOhost Research Databases<br>Search Screen - Advanced Search<br>Database - CINAHL Plus with Full Text	Display
S5	AB ((disaster# or catastrophe#) N5 (environ* or human* or manmade or "man made" or nature or natural or weather)) OR TI ((disaster# or catastrophe#) N5 (environ* or human* or manmade or "man made" or nature or natural or weather))	Expanders - Apply equivalent subjects<br>Search modes - Boolean/Phrase	Interface - EBSCOhost Research Databases<br>Search Screen - Advanced Search<br>Database - CINAHL Plus with Full Text	Display
S4	TX ((disaster or emergenc*) N2 victim*)	Expanders - Apply equivalent subjects<br>Search modes - Boolean/Phrase	Interface - EBSCOhost Research Databases<br>Search Screen - Advanced Search<br>Database - CINAHL Plus with Full Text	Display
S3	AB ((disaster or emergenc*) N2 victim*) OR TI ((disaster or emergenc*) N2 victim*)	Expanders - Apply equivalent subjects<br>Search modes - Boolean/Phrase	Interface - EBSCOhost Research Databases<br>Search Screen - Advanced Search<br>Database - CINAHL Plus with Full Text	Display
S2	(MH "Crime Victims") OR (MH "Victims")	Expanders - Apply equivalent subjects<br>Search modes - Boolean/Phrase	Interface - EBSCOhost Research Databases<br>Search Screen - Advanced Search	Display

			Database - CINAHL Plus with Full Text	
S1	(MH "Mass Casualty Incidents") OR (MM "Disasters+") OR (MH "Natural Disasters") OR (MM "Disaster Planning+")	Expanders - Apply equivalent subjects<br>Search modes - Boolean/Phrase	Interface - EBSCOhost Research Databases<br>Search Screen - Advanced Search<br>Database - CINAHL Plus with Full Text	Display

Database(s): Embase Classic and Embase 1947 to 2019 August 08

Search Strategy:

#	Searches	Results
1	disaster/ or mass disaster/ or natural disaster/ or emergency/	86210
2	Disaster planning/	12084
3	exp disaster victim/	393
4	((disaster or emergenc*) adj2 victim*).tw,kw.	890
5	((disaster? or catastrophe?) adj5 (environ* or human or manmade or "man made" or nature or natural or weather)).tw,kw.	6336
6	("mass casualty" or "mass casualties" or "mass fatalities" or "mass fatality").tw,kw.	2859
7	((crisis or crises) adj5 (environ* or human or manmade or "man made" or nature or natural or weather or setting*)).tw,kw.	1804
8	((crisis or crises or conflict) adj3 affected).tw,kw.	762
9	"warfare and armed conflicts"/ or armed conflict/ or warfare/ or ethnic cleansing/ or genocide/ or ethnic conflict/ or violence/	79963
10	exp warfare/ or military phenomena/	20954
11	war/ or war crime/ or war exposure/	37912
12	((warfare adj2 armed conflict?) or warfare or "war crime*" or "ethnic cleansing*" or "gas poisoning" or genocide or "war exposure").tw,kw.	7188
13	("afghan campaign" or "armed conflict" or "armed conflicts" or "gulf war" or "iraq war" or "war time" or "wartime" or "war torn" or "war affected" or "insurgency" or "intra conflict").tw,kw.	6603
14	((armed or zone or political or civil or setting*) adj3 (conflict or conflicts or attack or attacks or war or wars or "no fly")).tw,kw.	5184
15	(Fragile adj2 (state* or countr* or nation* or situation* or setting*)).tw,kw.	302
16	("Post conflict" or "postconflict" or "post war" or "post conflict setting" or "peacebuilding" or peacekeeping).tw,kw.	3338
17	(war adj2 related).tw,kw.	1042
18	("militant group?" or "militant organi#ation?" or militia or combatant? or rebel?).tw,kw.	1276
19	disaster medicine/ or emergency medicine/	40370
20	Epidemic/	113866

21	("disaster medicine" or "disaster outbreak*" or "epidemic*" or "disease outbreak?").tw,kw.	128788
22	emergency health service/	94227
23	((emergency or emergencies) adj5 (environ* or human or manmade or "man made" or nature or natural or weather or complex)).tw,kw.	3447
24	Starvation/	23138
25	(famine? or starvation?).tw,kw.	36761
26	drought/ or flooding/ or tsunami/ or hurricane/ or tornado/ or avalanche/ or earthquake/ or landslide/ or tsunami/ or volcanic ash/ or volcano/	31429
27	(avalanche? or cyclone? or drought? or earthquake? or flood* or hurricane? or landslide? or "land slide?" or mudslide? or "mud slide?" or storm? or tornado or tornadoes or tsunami? or typhoon? or "volcanic ash" or "volcanic eruption?" or "volcanic gases" or rubble).tw,kw.	69204
28	refugees/	11747
29	(evacuee? or refugee? or squatter? or transient? or "asylum seeker?").tw,kw.	371431
30	relief work/ or rescue work/	2216
31	((rescue or relief or aid) adj3 (work or plan? or activity or activities or agency or agencies)).tw,kw.	4517
32	((staff? or worker?) adj3 (relief or aid or rescue)).tw,kw.	1169
33	humanitarian assist*.tw,kw.	378
34	(humanitarian adj3 (aid or response or relief or crisis or crises or emergency or emergencies or disaster?)).tw,kw.	1574
35	Altruism/	7135
36	(humanitarianism or altruism).tw,kw.	3966
37	("displaced child*" or "displaced famil*" or "displaced individual?" or "displaced internally" or "displaced men" or "displaced people?" or "displaced person?" or "displaced population?" or "displaced women" or "displaced adolescent?" or "forced displacement?" or "forcibl* displace*" or "internal* displace*" or "population displace*" or "forced migration" or "migrant*").tw,kw.	20309
38	protected village.tw,kw.	5
39	((camp or camps) adj3 (refugee or transit or displace* or temporary or informal)).tw,kw.	1484
40	(Settlement adj3 (temporary or informal)).tw,kw.	252

41	adolescent/ or young adult/ or girl/	1834853
42	(adolescent? or teen* or youth? or "young adult?" or girl? or "young wom#n" or "young girl?").tw,kw.	684746
43	pregnant woman/ or exp mother/	227998
44	(mother? or female? or wom#n).tw,kw.	2985680
45	female/	9040461
46	(women adj2 reproductive adj2 age).tw,kw.	11683
47	Developing Country.sh.	93772
48	(Africa or Asia or Caribbean or West Indies or South America or Latin America or Central America).hw,kw,ti,ab,cp.	349217
49	(Afghanistan or Albania or Algeria or American Samoa or Angola or Armenia or Armenian or Azerbaijan or Bangladesh or Benin or Byelarus or Byelorussian or Belarus or Belorussian or Belorussia or Belize or Bhutan or Bolivia or Bosnia or Herzegovina or Hercegovina or Botswana or Brasil or Brazil or Bulgaria or Burkina Faso or Burkina Fasso or Upper Volta or Burundi or Urundi or Cambodia or Khmer Republic or Kampuchea or Cameroon or Cameroons or Cameron or Camerons or Cameroun or Cape Verde or Cabo Verde or Central African Republic or Chad or China or Colombia or Comoros or Comoro Islands or Comores or Mayotte or Congo or Zaire or Costa Rica or Cote d'Ivoire or Ivory Coast or Cuba or Djibouti or French Somaliland or Dominica or Dominican Republic or East Timor or East Timur or Timor Leste or Ecuador or Egypt or United Arab Republic or El Salvador or Equatorial Guinea or Eritrea or Ethiopia or Fiji or Gabon or Gabonese Republic or Gambia or Gaza or Georgia or Georgia Republic or Georgian Republic or Ghana or Gold Coast or Grenada or Guatemala or Guinea or Bissau or Guiana or Guyana or Haiti or Honduras or India or Maldives or Indonesia or Iran or Iraq or Jamaica or Jordan or Kazakhstan or Kazakh or Kenya or Kiribati or Korea or Democratic People's Republic of Korea or Kosovo or Kyrgyzstan or Kirghizia or Kyrgyz Republic or Kirghiz or Kirgizstan or Lao PDR or Laos or Lebanon or Lesotho or Basutoland or Liberia or Libya or Macedonia or Madagascar or Malagasy Republic or Malaysia or Malaya or Malay or Sabah or Sarawak or Malawi or Nyasaland or Mali or Marshall Islands or Mauritania or Mauritius or Agalega Islands or Mexico or Micronesia or Middle East or Moldova or Moldovia or Moldovian or Mongolia or Montenegro or Morocco or Ifni or Mozambique or Myanmar or Myanma or Burma or Namibia or Nauru or Nepal or Nicaragua or Niger or Nigeria or Pakistan or Papua New Guinea or Palestine or Paraguay or Peru or Philippines or	3343947

	Philipines or Phillipines or Phillippines or Romania or Rumania or Roumania or Russia or Russian or Rwanda or Ruanda or Saint Lucia or St Lucia or Saint Vincent or St Vincent or Grenadines or Samoa or Samoan Islands or Navigator Island or Navigator Islands or Sao Tome or Senegal or Serbia or Montenegro or Sierra Leone or Sri Lanka or Ceylon or Solomon Islands or Somalia or South Africa or Sudan or South Sudan or Suriname or Surinam or Swaziland or Eswatini or Syria or Syrian Arab Republic or Tajikistan or Tadzhikistan or Tadjikistan or Tadzhik or Tanzania or Thailand or Togo or Togolese Republic or Tonga or Tunisia or Turkey or Turkmenistan or Turkmen or Tuvalu or Uganda or Ukraine or USSR or Soviet Union or Union of Soviet Socialist Republics or Uzbekistan or Uzbek or Vanuatu or New Hebrides or Venezuela or Vietnam or Viet Nam or West Bank or Yemen or Yugoslavia or Zambia or Zimbabwe or Rhodesia).hw,kw,ti,ab,cp.	
50	((developing or less* developed or under developed or underdeveloped or middle income or low* income or underserved or under served or deprived or poor*) adj2 (countr* or nation? or population? or world)).tw,kw.	128475
51	((developing or less* developed or under developed or underdeveloped or middle income or low* income) adj2 (economy or economies)).tw,kw.	777
52	(low* adj2 (gdp or gnp or gross domestic or gross national)).tw,kw.	441
53	(low adj3 middle adj3 (countr* or nation*)).tw,kw.	16458
54	(lmic or lmics or third world or lami countr*).tw,kw.	8313
55	transitional countr*.tw,kw.	230
56	low income country/ or middle income country/	7612
57	or/47-56	3577601
58	Health status indicator/	2732
59	(indicator? or "health indicator?" or evaluation or tool* or methodolog* or standards).tw,kw.	3199214
60	(monitor* or surveillance or screening).tw,kw.	1909357
61	("rapid counting" or "aerial surveillance" or "flow monitoring" or "enumeration" or (reproductive health adj3 assessment toolkit)).tw,kw.	14615
62	Needs assessment/ or risk assessment/	545871
63	((rapid or needs) adj3 (assessment? or evaluation?)).tw,kw.	29136
64	exp questionnaire/ or data collection/ or "surveys and questionnaires"/ or "health survey"/ or contraceptive prevalence survey/ or health care survey/	1012875

65	("data collection tool*" or "data source*" or "data collection").tw,kw.	141670
66	(scale? or survey? or questionnaire? or measured or measurement or metric?).tw,kw.	4396116
67	(index or indices).tw,kw.	1198085
68	("minimum initial service package" or MISP).tw,kw.	84
69	Health services research/	32808
70	outcome assessment/	492273
71	(outcome? adj3 (health or measur* or assessment? or (score or scoring) or index or indices or scale? or monitor*)).tw,kw.	447335
72	(gender adj3 (analys* or analyz* or mainstreaming or marker or "risk assessment")).tw,kw.	16083
73	("gender empowerment measure" or "gender related development index" or "gender development index" or "gender gap index" or "global gender gap index" or "global gender gap report" or "gender intensity measure" or "gender inequality index" or "gender equity index" or "gender parity index" or "human development index").tw,kw.	1142
74	((social institutions adj2 gender index) or (survey adj2 women's empowerment index) or SWPER or (women's empowerment adj2 agriculture indec) or WEAI or (demographic adj2 health surveys)).tw,kw.	1650
75	((Africa gender adj2 development index) or "gender status index" or "African women's progress scoreboard" or "millennium development goals" or "sustainable development goals" or "gender mainstreaming scorecard" or "gender equality action").tw,kw.	3703
76	Gender/ or interpersonal relations/ or human rights/	362526
77	empowerment/ or women's rights/ or feminism/	17502
78	((gender or wom#n or women's or feminine* or female* or reproductive) adj5 violence).tw,kw.	9301
79	domestic violence/ or gender based violence/ or human trafficking/ or exp sexual violence/	44255
80	education/ or access to information/	438727
81	((gender or wom#n or women's or feminine* or female* or reproductive) adj3 (educat* or "school enrollment" or "school attendance" or literacy or (access adj2 information))).tw,kw.	29736
82	employment/ or occupation/ or unemployment/	125870

83	((gender or women or women's or feminine or female* or reproductive) adj3 (wage? or employ* or "labo?r force" or work* or "pay gap" or underemploy* or unemploy* or microfinance or microcredit or "occupational segregation" or economic or poverty or "basic needs" or financial or (cash adj2 transfer*) or voucher? or (saving? adj2 group?) or (self help adj2 group) or income)).tw,kw.	41520
84	life expectancy/ or birth rate/	62910
85	((gender or women or women's or feminine* or female* or reproductive) adj3 ("life expectancy" or "fertility rate" or "birth rate" or "son preference" or (sex ratio adj2 birth) or (devaluation adj2 daughters) or (fertility adj3 preference*))).tw,kw.	1575
86	((gender or women or women's or feminine* or female* or reproductive) adj3 (security or land or inheritance or marriage or "early marriage" or "parental authority")).tw,kw.	1857
87	marriage/ or forced marriage/ or ownership/	440355
88	politics/	62601
89	((gender or women or women's or feminine* or female* or reproductive) adj3 (politic* or "civil libert*" or (access adj2 public space) or "parliamentary seats")).tw,kw.	620
90	women's rights/ or human rights/ or gender/ or gender bias/ or human relation/	379135
91	((gender or women or women's or feminine* or female* or reproductive) adj3 (equal* or inequalit* or unequal* or justice* or injustice* or equit* or inequit* or disparit* or parit*)).tw,kw.	16354
92	((gender or women or women's or feminine* or female* or reproductive) adj3 (discriminat* or bias* or blind* or gap? or barrier?)).tw,kw.	14354
93	sex role/	4953
94	((gender or women or women's or feminine* or female* or reproductive) adj3 (role? or norm? or dynamic?)).tw,kw.	18442
95	((gender or women or women's or feminine* or female* or reproductive) adj3 transformat*).tw,kw.	471
96	sex difference/ or sex factor/	382610
97	((gender or wom#n or women's or feminine* or female* or reproductive) adj3 (difference? or stereotype? or perspective? or intersect* or sensiti#ation)).tw,kw.	82090
98	(gender adj3 traditional).tw,kw.	905
99	gender identity/ or socialization/ or social norm/	28889

100	(gender adj3 (sociali#ation or identity)).tw,kw.	4728
101	(gender adj3 (relat* or specific or responsive or prescription* or dimension*)).tw,kw.	37110
102	exp battered woman/	3229
103	((gender or wom#n or women's or feminine* or female* or reproductive) adj3 (victim* or abus* or "sex* crime" or "rape" or "assault" or "sexual harassment" or "sexual coercion" or "forced sex" or "sexual slavery” or “sex work" or battered or "sexual exploitation" or "human trafficking" or "restricted physical integrity" or patriarch* or "bodily integrity")).tw,kw.	11485
104	exp attitude/	733204
105	reproductive behavior/ or contraceptive behavior/	5248
106	(Attitude? adj5 (violence or "wife beating" or "refusing sex")).tw,kw.	828
107	self concept/ or personal autonomy/ or self esteem/	119985
108	((gender or wom#n or women's or female or girl or reproductive) adj3 empower*).tw,kw.	2191
109	(empower* adj3 (social or economic or health or legal or financial or political or psychological)).tw,kw.	2532
110	("safe space?" or "female friendly space?").tw,kw.	473
111	((wom#n or women's or girl? or female) adj5 ("safe space?" or "friendly space?")).tw,kw.	19
112	reproductive rights/ or decision making/	219842
113	((gender or wom#n or women's or female or girl) adj3 (disempower* or autonomy or participation or status or preference* or power or agency or right* or feminism or authorit* or control* or leadership or choice or voice or "critical consciousness" or working or mobility or freedom or ownership or assets or (contribution adj2 family income) or "reproductive rights" or movement or training or "labo?r participation" or "self esteem" or "collective action*" or "self-efficacy" or "self efficacy" or "skill* building" or "self respect" or "self worth" or "solidarity")).tw,kw.	120886
114	((wom#n or women's or female) adj5 ("decision making" or decision-making)).tw,kw.	2411
115	household/ or household income/ or socioeconomics/ or social status/ or educational status/ or poverty/ or family size/ or self help/	366112
116	(decision making adj3 (power or joint or domestic or intrahousehold or "intra household" or influence)).tw,kw.	3912
117	help seeking behavior/ or interpersonal communication/	175903

118	Health care delivery/ or health equity/ or health care quality/ or health care access/	417642
119	or/1-40	1003148
120	or/41-46	10097758
121	or/58-75	9630068
122	or/76-118	3272002
123	57 and 119 and 120 and 121 and 122	12323
124	Limit 123 to (English language and yr=”2004-2019”)	

Database(s): Ovid MEDLINE: Epub Ahead of Print, In-Process & Other Non-Indexed Citations, Ovid MEDLINE® Daily and Ovid MEDLINE® 1946-Present

Search Strategy:

#	Searches	Results	Annotations
1	disasters/ or emergencies/ or mass casualty incidents/ or natural disasters/	59118	
2	disaster victims/	163	
3	((disaster or emergenc*) adj2 victim*).tw,kf.	689	
4	((disaster or disasters or catastrophe or catastrophes) adj5 (environ* or human or manmade or "man made" or nature or natural or weather)).tw,kf.	5378	
5	("mass casualty" or "mass casualties" or "mass fatalities" or "mass fatality").tw,kf.	2320	
6	((crisis or crises) adj5 (environ* or human or manmade or "man made" or nature or natural or weather or setting*)).tw,kf.	1374	
7	((crisis or crises or conflict) adj3 affected).tw,kf.	768	
8	"warfare and armed conflicts"/ or armed conflict/ or warfare/ or war crimes/ or ethnic cleansing/ or genocide/ or war exposure/	24460	
9	((warfare adj2 armed conflict?) or warfare or "armed conflict?" or "war crime*" or "ethnic cleansing*" or "gas poisoning" or genocide or (war* adj3 exposure)).tw,kf.	9521	
10	afghan campaign 2001-/ or gulf war/ or iraq war, 2003-2011/	4077	
11	("afghan campaign" or "gulf war" or "iraq war" or "war time" or "wartime" or "war torn" or "war affected" or "insurgency" or "intra conflict").tw,kf.	4700	
12	((armed or zone or political or civil or setting*) adj3 (conflict? or attack? or war? or "no fly")).tw,kf.	5545	
13	(Fragile adj2 (state* or countr* or nation* or situation* or setting*)).tw,kf.	260	
14	("Post conflict" or "postconflict" or "post war" or "post conflict setting" or "peacebuilding" or peacekeeping).tw,kf.	2574	
15	(war adj3 related).tw,kf.	958	

16	("militant group?" or "militant organization?" or "militant organisation?" or militia or combatant? or rebel?).tw,kf.	1071	
17	Disaster Medicine/	799	
18	disease outbreaks/	76677	
19	Epidemics/	9333	
20	("disaster medicine" or "disaster outbreak*" or "epidemic*" or "disease outbreak?").tw,kf.	103972	
21	Emergency Medical Services/	40957	
22	((emergency or emergencies) adj5 (environ* or human or manmade or "man made" or nature or natural or weather or complex)).tw,kf.	2529	
23	Starvation/	9740	
24	(famine? or starvation?).tw,kf.	30620	
25	cyclonic storms/ or droughts/ or floods/ or tornadoes/ or tidal waves/	11154	
26	avalanches/ or earthquakes/ or landslides/ or tidal waves/ or tsunamis/ or volcanic eruptions/	5565	
27	(avalanche? or cyclone? or drought? or earthquake? or flood* or hurricane? or landslide? or "land slide?" or mudslide? or "mud slide?" or storm? or tornado* or tsunami? or typhoon? or "volcanic ash" or "volcanic eruption?" or "volcanic gases" or rubble).tw,kf.	61067	
28	refugees/ or exp refugee camps/	9496	
29	(evacuee? or refugee? or squatter? or transients or "asylum seeker?").tw,kf.	24875	
30	relief work/ or rescue work/	5763	
31	((rescue or relief or aid) adj3 (work or plan? or activit* or agency or agencies)).tw,kf.	3431	
32	((staff? or worker?) adj3 (relief or aid or rescue)).tw,kf.	923	
33	humanitarian assist*.tw,kf.	341	
34	(humanitarian adj3 (aid or response or relief or crisis or crises or emergency or emergencies or disaster or disasters)).tw,kf.	1429	
35	Altruism/	6488	
36	(humanitarianism or altruism).tw,kf.	3263	

37	("displaced child*" or "displaced famil*" or "displaced individual?" or "displaced internally" or "displaced men" or "displaced people?" or "displaced person?" or "displaced population?" or "displaced wom#n" or "displaced adolescent?" or "forced displacement" or "forcibl* displace*" or "internal* displace*" or "population displace*" or "forced migration" or "migrant*").tw,kf.	19850	
38	(protected adj3 village*).tw,kf.	15	
39	((camp or camps) adj3 (refugee or transit or displace* or temporary or informal)).tw,kf.	1333	
40	(Settlement? adj3 (temporary or informal)).tw,kf.	522	
41	Developing Countries.sh,kf.	83789	
42	(Africa or Asia or Caribbean or West Indies or South America or Latin America or Central America).hw,kf,ti,ab,cp.	261441	
43	(Afghanistan or Albania or Algeria or American Samoa or Angola or Armenia or Armenian or Azerbaijan or Bangladesh or Benin or Byelarus or Byelorussian or Belarus or Belorussian or Belorussia or Belize or Bhutan or Bolivia or Bosnia or Herzegovina or Hercegovina or Botswana or Brasil or Brazil or Bulgaria or Burkina Faso or Burkina Fasso or Upper Volta or Burundi or Urundi or Cambodia or Khmer Republic or Kampuchea or Cameroon or Cameroons or Cameron or Camerons or Cameroun or Cape Verde or Cabo Verde or Central African Republic or Chad or China or Colombia or Comoros or Comoro Islands or Comores or Mayotte or Congo or Zaire or Costa Rica or Cote d'Ivoire or Ivory Coast or Cuba or Djibouti or French Somaliland or Dominica or Dominican Republic or East Timor or East Timur or Timor Leste or Ecuador or Egypt or United Arab Republic or El Salvador or Equatorial Guinea or Eritrea or Ethiopia or Fiji or Gabon or Gabonese Republic or Gambia or Gaza or Georgia or Georgia Republic or Georgian Republic or Ghana or Gold Coast or Grenada or Guatemala or Guinea or Bissau or Guiana or Guyana or Haiti or Honduras or India or Maldives or Indonesia or Iran or Iraq or Jamaica or Jordan or Kazakhstan or Kazakh or Kenya or Kiribati or Korea or Democratic People's Republic of Korea or Kosovo or Kyrgyzstan or Kirghizia or Kyrgyz Republic or Kirghiz or Kirgizstan or Lao PDR or Laos or Lebanon or	2701363	

	Lesotho or Basutoland or Liberia or Libya or Macedonia or Madagascar or Malagasy Republic or Malaysia or Malaya or Malay or Sabah or Sarawak or Malawi or Nyasaland or Mali or Marshall Islands or Mauritania or Mauritius or Agalega Islands or Mexico or Micronesia or Middle East or Moldova or Moldovia or Moldovian or Mongolia or Montenegro or Morocco or Ifni or Mozambique or Myanmar or Myanma or Burma or Namibia or Nauru or Nepal or Nicaragua or Niger or Nigeria or Pakistan or Papua New Guinea or Palestine or Paraguay or Peru or Philippines or Philipines or Phillipines or Phillippines or Romania or Rumania or Roumania or Russia or Russian or Rwanda or Ruanda or Saint Lucia or St Lucia or Saint Vincent or St Vincent or Grenadines or Samoa or Samoan Islands or Navigator Island or Navigator Islands or Sao Tome or Senegal or Serbia or Montenegro or Sierra Leone or Sri Lanka or Ceylon or Solomon Islands or Somalia or South Africa or Sudan or South Sudan or Suriname or Surinam or Swaziland or Eswatini or Syria or Syrian Arab Republic or Tajikistan or Tadzhikistan or Tadjikistan or Tadzhik or Tanzania or Thailand or Togo or Togolese Republic or Tonga or Tunisia or Turkey or Turkmenistan or Turkmen or Tuvalu or Uganda or Ukraine or USSR or Soviet Union or Union of Soviet Socialist Republics or Uzbekistan or Uzbek or Vanuatu or New Hebrides or Venezuela or Vietnam or Viet Nam or West Bank or Yemen or Yugoslavia or Zambia or Zimbabwe or Rhodesia).hw,kf,ti,ab,cp.		
44	((developing or less* developed or under developed or underdeveloped or middle income or low* income or underserved or under served or deprived or poor*) adj2 (countr* or nation? or population? or world)).tw,kf.	126236	
45	((developing or less* developed or under developed or underdeveloped or middle income or low* income) adj2 (economy or economies)).tw,kf.	625	
46	(low* adj2 (gdp or gnp or gross domestic or gross national)).tw,kf.	311	
47	(low adj3 middle adj3 (countr* or nation*)).tw,kf.	14155	
48	transitional countr*.tw,kf.	156	

49	41 or 42 or 43 or 44 or 45 or 46 or 47 or 48	2859628	Context 1.2 - Low and middle income countries
50	adolescent/ or young adult/	2299722	
51	(adolescent? or teen* or youth? or "young adult?" or girl? or "young wom#n" or "young girl?").tw,kf.	496414	
52	exp Pregnant Women/ or exp Women/ or mothers/	74136	
53	(mother? or female? or wom#n).tw,kf.	2006302	
54	(women adj2 reproductive adj2 age).tw,kf.	8204	
55	Health status indicators/	22952	
56	(indicator? or "health indicator?" or evaluation or tool? or methodolog* or standards).tw,kf.	2336952	
57	(monitor* or surveillance or screening).tw,kf.	1355515	
58	("rapid counting" or "aerial surveillance" or "flow monitoring" or "enumeration" or (reproductive health adj3 assessment toolkit)).tw,kf.	11090	
59	((rapid or needs) adj3 (assessment? or evaluation?)).tw,kf.	19851	
60	("data collection tool?" or "data source?" or "data collection").tw,kf.	110301	
61	data collection/ or "surveys and questionnaires"/ or contraceptive prevalence surveys/ or health care surveys/ or health surveys/	583028	
62	(scale? or survey? or questionnaire? or measured or measurement or metric?).tw,kf.	3255135	
63	(index or indices).tw,kf.	827432	
64	("minimum initial service package" or MISP).tw,kf.	62	
65	(outcome? adj3 (health or measur* or assessment? or (score or scoring) or index or indices or scale? or monitor*)).tw,kf.	338812	
66	"Outcome Assessment (Health Care)"/	68579	
67	health services research/ or needs assessment/ or risk assessment/	307024	
68	(gender adj3 (analys#s or analy#e or analy#ing or mainstreaming or marker or "risk assessment")).tw,kf.	7798	

69	("gender empowerment measure" or "gender related development index" or "gender development index" or "gender gap index" or "global gender gap index" or "global gender gap report" or "gender intensity measure" or "gender inequality index" or "gender equity index" or "gender parity index" or "human development index").tw,kf.	911	
70	((Africa gender adj2 development index) or "gender status index" or "African women's progress scoreboard" or "millennium development goals" or "sustainable development goals" or "gender mainstreaming scorecard" or "gender equality action").tw,kf.	3197	
71	((social institutions adj2 gender index) or (survey adj2 women's empowerment index) or SWPER or (women's empowerment adj2 agriculture index) or WEAI or (demographic adj2 health surveys)).tw,kf.	1746	
72	((gender or wom#n or women's or feminine* or female* or reproductive) adj5 violence).tw,kf.	8333	
73	((gender or wom#n or women's or feminine* or female* or reproductive) adj3 (educat* or "school enrollment" or "school attendance" or literacy or (access adj2 information))).tw,kf.	22319	
74	((gender or wom#n or women's or feminine* or female* or reproductive) adj3 (wage? or employ* or "labo?r force" or "pay gap" or underemploy* or unemploy* or work or microfinance or microcredit or "occupational segregation" or economic or poverty or "basic needs" or financ* or assets? or (cash adj2 transfer*) or "voucher?" or (saving? adj2 group?) or (self help adj2 group?) or income)).tw,kf.	22844	
75	((gender or wom#n or women's or feminine* or female* or reproductive) adj3 ("life expectancy" or "fertility rate" or "birth rate" or "son preference" or (sex ratio adj2 birth) or (devaluation adj2 daughters) or (fertility adj3 preference?))).tw,kf.	1440	
76	((gender or wom#n or women's or feminine* or female* or reproductive) adj3 (security or land or inheritance or marriage or "early marriage" or "parental authority")).tw,kf.	1844	

77	((gender or wom#n or women's or feminine* or female* or reproductive) adj3 (politic* or "civil libert*" or (access adj2 public space) or "parliamentary seats")).tw,kf.	718	
78	Gender/ or interpersonal relations/ or human rights/ or sexism/	100250	
79	exp "Power (Psychology)"/	12387	
80	("gender equality" or "gender inequality").tw,kf.	1711	
81	((gender or wom#n or women's or feminine* or female* or reproductive) adj3 (equal* or inequalit* or justice* or injustice* or equit* or inequit* or disparit* or parit*)).tw,kf.	12470	
82	((gender or wom#n or women's or feminine* or female* or reproductive) adj3 (discriminat* or bias* or blind* or gap? or barrier?)).tw,kf.	12488	
83	Gender Identity/ or sex factors/ or interpersonal relations/	334184	
84	"gender identity".tw,kf.	2793	
85	women's rights/ or feminism/ or reproductive rights/ or socioeconomic factors/ or attitudes/	200194	
86	((gender or wom#n or women's or feminine* or female* or reproductive) adj3 (role? or norm? or dynamic? or attitude? or resilience)).tw,kf.	19784	
87	((gender or wom#n or women's or feminine* or female* or reproductive) adj3 transformat*).tw,kf.	396	
88	((gender or wom#n or women's or feminine* or female* or reproductive) adj3 (difference? or stereotype? or perspective? or intersect* or sensiti#ation)).tw,kf.	57245	
89	(gender adj3 traditional).tw,kf.	778	
90	gender sociali#ation.tw,kf.	137	
91	(gender adj3 (relat? or specific or responsive or prescription* or dimension)).tw,kf.	13321	
92	((gender or wom#n or women's or female or girl) adj3 (disempower* or autonomy or participation or status or preference* or power or agency or right? or feminism or authori* or control* or leadership or choice or voice or working or mobility or ownership or assets or freedom or movement or awareness or	89109	

	(contribution adj2 family income) or "reproductive rights" or training or "labo?r participation" or "self esteem" or "collective action*" or "skill? building" or "self efficacy" or "self-efficacy" or "self worth" or "self respect")).tw,kf.		
93	exp Decision Making/ or choice behavior/	190438	
94	((women or women's or female) adj3 ("decision making" or "decision-making")).tw,kf.	1291	
95	(attitude* adj5 (violence or "wife beating" or "refusing sex")).tw,kf.	752	
96	("safe space?" or "female friendly space?").tw,kf.	330	
97	((gender or wom#n or women's or female or girl or reproductive) adj3 empower*).tw,kf.	2106	
98	gender based violence/ or child abuse, sexual/ or rape/ or intimate partner violence/ or spouse abuse/ or sexual harassment/ or sex work/ or battered women/ or human trafficking/	32163	
99	((gender or wom#n or women's or feminine* or female* or reproductive) adj3 (victim* or abus* or "sex* crime" or "rape" or "assault" or "sexual harassment" or "sexual coercion" or "forced sex" or "sexual slavery" or "sex work" or battered or "sexual exploitation" or "human trafficking" or integrity)).tw,kf.	9983	
100	Life expectancy/ or birth rate/	25508	
101	employment/ or unemployment/	49812	
102	education/ or socioeconomic factors/ or economic status/ or educational status/ or family characteristics/ or medical indigency/ or poverty/	251993	
103	personal autonomy/ or self concept/ or self-help groups/	79134	
104	reproductive behavior/ or contraception behavior/ or help seeking behavior/	9312	
105	(decision making adj3 (power or joint or domestic or intrahousehold or "intra household" or influence)).tw,kf.	2997	
106	socialization/ or social norms/ or politics/ or marriage/ or access to information/ or ownership/	91959	
107	exp Women, Working/	5243	

108	(empower* adj3 (social or economic or health or legal or financial or political or psychological)).tw,kf.	2179	
109	quality-adjusted life years/ or Health Knowledge, Attitudes, Practice/ or attitude to health/	190283	
110	health services accessibility/ or "quality of health care"/ or health equity/	135831	
111	or/1-40	443442	
112	or/55-71	6873867	
113	or/50-54	4083707	
114	or/72-110	1383141	
115	49 and 111 and 112 and 113 and 114	5931	

<u>Database(s): PAIS Index</u>

Search Strategy:

Set#: S1

Searched for: AB,IF,SU,TI,MESH("disaster*" OR "emergenc*" OR "mass casualty incident*" OR "conflict* " OR "refugee* " OR "migrant* " OR " forced displace* " OR "natural disaster*" OR "war related" OR "disaster victim" OR "starvation" OR "outbreak*" OR "epidemic" OR "famine" OR "drought" OR "flood*" OR "complex emergenc*" OR "post conflict" OR "post war" OR "armed conflict" OR "conflict related" OR "relief work" OR "rescue work" OR "displace*" OR "asylum seeker" OR "forced migration" OR "internal displace*" OR "warfare" OR "war" OR "cyclon*" OR "hurricane" OR "earthquake" OR "typhoon" OR "crisis" OR "humanitarian" OR "humanitarian aid")

Databases: PAIS Index

Results: 227444

Set#: S2

Searched for: AB,IF,SU,TI,MESH("developing countries" OR "Africa" OR "Asia" OR "Caribbean" OR "West Indies" OR "South America" OR "Latin America" OR "Central America" OR "low* income countr*" OR "middle income countr*" OR "Low N/2 middle income" OR "Afghanistan" OR "Albania" OR "Algeria" OR "American Samoa" OR "Angola" OR "Armenia" OR "Armenian" OR Azerbaijan OR Bangladesh OR Benin OR Byelarus OR Byelorussian OR Belarus OR Belorussian OR Belorussia OR Belize OR Bhutan OR Bolivia OR Bosnia OR Herzegovina OR Hercegovina OR Botswana OR Brasil OR Brazil OR Bulgaria OR "Burkina Faso" OR "Burkina Fasso" OR "Upper Volta" OR Burundi OR Urundi OR Cambodia OR "Khmer Republic" OR Kampuchea OR Cameroon OR Cameroons OR Cameron OR Camerons OR Cameroun OR "Cape Verde" OR "Cabo Verde" OR "Central African Republic" OR Chad OR China OR Colombia OR Comoros OR Comoro Islands OR Comores OR Mayotte OR Congo OR Zaire OR "Costa Rica" OR "Cote d'Ivoire" OR "Ivory Coast" OR Cuba OR Djibouti OR "French Somaliland" OR Dominica OR "Dominican Republic" OR "East Timor" OR "East Timur" OR "Timor Leste" OR Ecuador OR Egypt OR "United Arab Republic" OR "El Salvador" OR "Equatorial Guinea" OR Eritrea OR Ethiopia OR Fiji OR Gabon OR "Gabonese Republic" OR Gambia OR Gaza OR Georgia OR "Georgia Republic" OR "Georgian Republic" OR Ghana OR "Gold Coast" OR Grenada OR Guatemala OR Guinea OR Bissau OR Guiana OR Guyana OR Haiti OR Honduras OR India OR Maldives OR Indonesia OR Iran OR Iraq OR Jamaica OR Jordan OR Kazakhstan OR Kazakh OR Kenya OR Kiribati OR Korea OR Kosovo OR Kyrgyzstan OR Kirghizia OR "Kyrgyz Republic" OR Kirghiz OR Kirgizstan OR "Lao PDR" OR Laos OR Lebanon OR Lesotho OR Basutoland OR Liberia OR Libya OR Macedonia OR Madagascar OR "Malagasy Republic" OR Malaysia OR Malaya OR Malay OR Sabah OR Sarawak OR Malawi OR Nyasaland OR Mali OR "Marshall Islands" OR Mauritania OR Mauritius OR "Agalega Islands" OR Mexico OR Micronesia OR "Middle East" OR Moldova OR Moldovia OR Moldovian OR Mongolia OR Montenegro OR Morocco OR Ifni OR Mozambique OR Myanmar OR Myanma OR Burma OR Namibia OR Nauru OR Nepal OR Nicaragua OR Niger OR Nigeria OR Pakistan OR "Papua New Guinea" OR Palestine OR Paraguay OR Peru OR Philippines OR Philipines OR Phillipines OR Phillippines OR Romania OR Rumania OR Roumania OR Russia OR Russian OR Rwanda OR Ruanda OR "Saint Lucia" OR "St Lucia" OR "Saint Vincent" OR "St Vincent" OR Grenadines OR Samoa OR "Samoan Islands" OR "Navigator Island" OR "Navigator Islands" OR "Sao Tome" OR Senegal OR Serbia OR Montenegro OR "Sierra Leone" OR "Sri Lanka" OR Ceylon OR "Solomon Islands" OR Somalia OR "South Africa" OR Sudan OR "South Sudan" OR Suriname OR Surinam OR Swaziland OR Eswatini OR Syria OR "Syrian Arab Republic" OR Tajikistan OR Tadzhikistan OR Tadjikistan OR Tadzhik OR Tanzania OR Thailand OR Togo OR "Togolese Republic" OR Tonga OR Tunisia OR Turkey OR Turkmenistan OR Turkmen OR Tuvalu OR Uganda OR Ukraine OR USSR OR "Soviet Union" OR Uzbekistan OR Uzbek OR Vanuatu OR "New

Hebrides" OR Venezuela OR Vietnam OR "Viet Nam" OR "West Bank" OR Yemen OR Yugoslavia OR Zambia OR Zimbabwe OR Rhodesia)
Databases: PAIS Index
Results: 489942

Set#: S3
Searched for: AB,IF,TI,SU,MESH(adolescent OR girl OR wom*n OR "young adult" OR mother OR female OR teen*)
Databases: PAIS Index
Results: 70924

Set#: S5
Searched for: AB,IF,SU,TI,MESH("Health status indicators" OR "indicators" OR "health indicator" OR "evaluation" OR "measure*" OR "tool*" OR "assessment" OR "methodolog*" OR "standard*" OR "data collection" OR "surveys and questionnaires" OR "health care surveys" OR "health surveys" OR "scale*" OR "survey*" OR "questionnaire*" OR "psychometric*" OR "monitoring" OR "needs assessment" OR research OR "data source" OR index OR indices OR "health services research" OR metric OR "millenium development goals" OR "sustainable development goals" OR scorecard OR scoreboard)
Databases: PAIS Index
Results: 403171

Set#: S6
Searched for: Su,AB,TI,IF,MESH(gender OR empower* OR equality OR inequality OR equity OR "women's empowerment" OR "women's rights" OR "human rights" OR "sex role" OR mainstreaming) OR SU,AB,TI,IF,MESH(violence OR education* OR school OR literacy OR wage OR employ* OR labo?r OR "pay gap" OR underemploy* OR unemploy* OR microfinance OR microcredit OR financial OR "cash transfer" OR "voucher" OR saving OR "self help" OR "life expectancy" OR "fertility rate" OR "birth rate" OR parenting OR "son preference" OR "decision making" OR security OR land OR inheritance OR marriage OR "parental authority" OR politic* OR economic OR power OR choice OR voice OR "civil liberties" OR "civil liberty" OR participation OR "safe space" OR "female friendly space" OR freedom OR mobility OR reproductive OR maternal OR health OR leadership OR training OR "skill building" OR "health care delivery" OR access OR quality OR household OR socioeconomic OR "self concept" OR "self efficacy" OR "self esteem" OR autonomy OR socialisation OR "social norm" OR transformat*)
Databases: PAIS Index
Results: 1204464

Set#: S7
Searched for: S1 AND S2 AND S3 AND S5 AND S6
Databases: PAIS Index
These databases are searched for part of your query.
Results: 1213

Database(s): PsycINFO 1806 to August Week 1 2019

Search Strategy:

#	Searches	Results
1	natural disasters/ or disasters/ or emergency management/ or emergency preparedness/ or emergency services/	16929
2	((disaster or emergenc*) adj2 victim*).tw,id.	419
3	((disaster? or catastrophe?) adj5 (environ* or human or manmade or "man made" or nature or natural or weather)).tw,id.	4193
4	("mass casualty" or "mass casualties" or "mass fatalities" or "mass fatality").tw,id.	176
5	((crisis or crises) adj5 (environ* or human or manmade or "man made" or nature or natural or weather or setting*)).tw,id.	1397
6	((crisis or crises or conflict) adj3 affected).tw,id.	587
7	war/ or conflict/ or violence/ or crises/	65708
8	genocide/ or mass murder/	1161
9	("warfare and armed conflict*" or warfare or "armed conflict*" or "war crime*" or "ethnic cleansing*" or "gas poisoning" or genocide or "war exposure").tw,id.	4981
10	("afghan campaign" or "gulf war" or "iraq war" or "war time" or "wartime" or "war torn" or "war affected" or "insurgency" or "intra conflict").tw,id.	3967
11	((armed or zone or political or civil or setting*) adj3 (conflict or conflicts or attack or attacks or war or wars or "no fly")).tw,id.	5360
12	(Fragile adj2 (state* or countr* or nation* or situation* or setting*)).tw,id.	137
13	("Post conflict" or "postconflict" or "post war" or "post conflict setting" or "peacebuilding" or peacekeeping).tw,id.	3238
14	(war adj2 related).tw,id.	898
15	("militant group?" or "militant organization" or "militant organizations" or "militant organisation" or "militant organisations" or militia or combatant or rebel*).tw,id.	3309
16	epidemics/ or pandemics/	3219
17	("disaster medicine" or "disaster outbreak*" or "disease outbreak?" or "epidemic*").tw,id.	12347
18	((emergency or emergencies) adj5 (environ* or human or manmade or "man made" or nature or natural or weather or complex)).tw,id.	596
19	starvation/	382

20	(famine or famines or starvation or starvations).tw,id.	2169
21	(avalanche? or cyclone? or drought? or earthquake? or flood* or hurricane? or landslide? or "land slide?" or mudslide? or "mud slide?" or storm? or tornado* or tsunami? or typhoon? or "volcanic ash" or "volcanic eruption?" or "volcanic gases" or rubble).tw,id.	10783
22	refugees/ or asylum seeking/	5711
23	(evacuee? or refugee? or squatter? or transient? or "asylum seeker?").tw,id.	31817
24	rescue workers/	239
25	((rescue or relief or aid) adj3 (work or plan? or activity or activities or agency or agencies)).tw,id.	1111
26	((staff? or worker?) adj3 (relief or aid or rescue)).tw,id.	694
27	prosocial behavior/ or altruism/	10785
28	humanitarian assist*.tw,id.	128
29	(humanitarian adj3 (aid or response or relief or crisis or crises or emergency or emergencies or disaster?)).tw,id.	647
30	(humanitarianism or altruism).tw,id.	6161
31	("displaced child*" or "displaced famil*" or "displaced individual?" or "displaced internally" or "displaced men" or "displaced people?" or "displaced person?" or "displaced population?" or "displaced wom#n" or "displaced adolescent?" or "forced displacement?" or "forcibl* displace*" or "internal* displace*" or "population displace*" or "forced migration" or "migrant*").tw,id.	10656
32	((camp or camps) adj3 (refugee or transit or displace* or temporary or informal)).tw,id.	632
33	protected village?.tw,id.	1
34	(Settlement? adj3 (temporary or informal)).tw,id.	173
35	developing countries/	5288
36	Developing Countries.sh,id.	5584
37	(Africa or Asia or Caribbean or West Indies or South America or Latin America or Central America).hw,id,ti,ab,cp.	38309
38	(Afghanistan or Albania or Algeria or American Samoa or Angola or Armenia or Armenian or Azerbaijan or Bangladesh or Benin or Byelarus or Byelorussian or Belarus or Belorussian or Belorussia or Belize or Bhutan or Bolivia or Bosnia or Herzegovina or Hercegovina or Botswana or Brasil or Brazil or Bulgaria or Burkina Faso or Burkina	201658

	Fasso or Upper Volta or Burundi or Urundi or Cambodia or Khmer Republic or Kampuchea or Cameroon or Cameroons or Cameron or Camerons or Cameroun or Cape Verde or Cabo Verde or Central African Republic or Chad or China or Colombia or Comoros or Comoro Islands or Comores or Mayotte or Congo or Zaire or Costa Rica or Cote d'Ivoire or Ivory Coast or Cuba or Djibouti or French Somaliland or Dominica or Dominican Republic or East Timor or East Timur or Timor Leste or Ecuador or Egypt or United Arab Republic or El Salvador or Equatorial Guinea or Eritrea or Ethiopia or Fiji or Gabon or Gabonese Republic or Gambia or Gaza or Georgia or Georgia Republic or Georgian Republic or Ghana or Gold Coast or Grenada or Guatemala or Guinea or Bissau or Guiana or Guyana or Haiti or Honduras or India or Maldives or Indonesia or Iran or Iraq or Jamaica or Jordan or Kazakhstan or Kazakh or Kenya or Kiribati or Korea or Democratic People's Republic of Korea or Kosovo or Kyrgyzstan or Kirghizia or Kyrgyz Republic or Kirghiz or Kirgizstan or Lao PDR or Laos or Lebanon or Lesotho or Basutoland or Liberia or Libya or Macedonia or Madagascar or Malagasy Republic or Malaysia or Malaya or Malay or Sabah or Sarawak or Malawi or Nyasaland or Mali or Marshall Islands or Mauritania or Mauritius or Agalega Islands or Mexico or Micronesia or Middle East or Moldova or Moldovia or Moldovian or Mongolia or Montenegro or Morocco or Ifni or Mozambique or Myanmar or Myanma or Burma or Namibia or Nauru or Nepal or Nicaragua or Niger or Nigeria or Pakistan or Papua New Guinea or Palestine or Paraguay or Peru or Philippines or Philipines or Phillipines or Phillippines or Romania or Rumania or Roumania or Russia or Russian or Rwanda or Ruanda or Saint Lucia or St Lucia or Saint Vincent or St Vincent or Grenadines or Samoa or Samoan Islands or Navigator Island or Navigator Islands or Sao Tome or Senegal or Serbia or Montenegro or Sierra Leone or Sri Lanka or Ceylon or Solomon Islands or Somalia or South Africa or Sudan or South Sudan or Suriname or Surinam or Swaziland or Eswatini or Syria or Syrian Arab Republic or Tajikistan or Tadzhikistan or Tadjikistan or Tadzhik or Tanzania or Thailand or Togo or Togolese Republic or Tonga or Tunisia or Turkey or Turkmenistan or Turkmen or Tuvalu or Uganda or Ukraine or USSR or Soviet Union or Union of Soviet Socialist Republics or Uzbekistan or Uzbek or Vanuatu or New Hebrides or Venezuela or Vietnam or Viet Nam or West Bank or Yemen or Yugoslavia or Zambia or Zimbabwe or Rhodesia).hw,id,ti,ab,cp.	
39	((developing or less* developed or under developed or underdeveloped or middle income or low* income or underserved or under served or deprived or poor*) adj2 (countr* or nation? or population? or world)).tw,id.	18959

40	((developing or less* developed or under developed or underdeveloped or middle income or low* income) adj2 (economy or economies)).tw,id.	449
41	(low* adj2 (gdp or gnp or gross domestic or gross national)).tw,id.	53
42	(lmic or lmics or third world or lami countr*).tw,id.	1762
43	transitional countr*.tw,id.	64
44	35 or 36 or 37 or 38 or 39 or 40 or 41 or 42 or 43	229034
45	(adolescent? or teen* or youth? or "young adult?" or girl? or "young wom#n" or "young girl?").tw,id.	362221
46	exp mothers/	39435
47	exp human females/	142078
48	"mother?".tw,id.	120265
49	wom#n.tw,id.	287131
50	(women adj2 reproductive adj2 age).tw,id.	729
51	("health status indicator?" or indicator? or "health indicator?" or evaluation or tool? or methodolog* or standards).tw,id.	582164
52	(monitor* or surveillance or screening).tw,id.	156498
53	("rapid counting" or "aerial surveillance" or "flow monitoring" or "enumeration" or (reproductive health adj3 assessment toolkit)).tw,id.	1151
54	evaluation/ or needs assessment/ or program evaluation/ or measurement/ or risk assessment/	96305
55	((rapid or needs) adj3 (assessment or evaluation)).tw,id.	6882
56	data collection/ or methodology/ or surveys/	47567
57	("data collection tool*" or "data source*" or "data collection").tw,id.	38211
58	(scale? or survey? or questionnaire? or measured or measurement or metric?).tw,id.	970693
59	questionnaires/	18074
60	(index or indices).tw,id.	170943
61	"index (testing)"/	193
62	("minimum initial service package" or MISP).tw,id.	3
63	psychometrics/	58802
64	(outcome* adj3 (health or measur* or assess* or (score or scoring) or index or indices or scale* or monitor*)).tw,id.	85871

65	(gender adj3 (analys* or analy#ing or mainstreaming or marker or "risk assessment")).tw,id.	4734
66	("gender empowerment measure" or "gender related development index" or "gender development index" or "gender gap index" or "global gender gap index" or "global gender gap report" or "gender intensity measure" or "gender inequality index" or "gender equity index" or "gender parity index" or "human development index").tw,id.	333
67	((social institutions adj2 gender index) or (survey adj2 women's empowerment index) or SWPER or (women's empowerment adj2 agriculture indec) or WEAI or (demographic adj2 health surveys)).tw,id.	339
68	((Africa gender adj2 development index) or "gender status index" or "African women's progress scoreboard" or "millennium development goals" or "sustainable development goals" or "gender mainstreaming scorecard" or "gender equality action").tw,id.	654
69	monitoring/	8071
70	Gender Equality/ or Gender Identity/ or Gender Gap/	9054
71	interpersonal relationships/ or human rights/	23423
72	empowerment/ or feminism/ or sexism/	20440
73	((gender or wom#n or women's or feminine* or female* or reproductive) adj5 violence).tw,id.	11361
74	Sexual Abuse/ or rape/ or partner abuse/ or intimate partner violence/ or domestic violence/ or sexual harassment/ or battered females/ or human trafficking/ or physical abuse/	49156
75	((gender or wom#n or women's or feminine* or female* or reproductive) adj3 (victim* or abus* or "sex* crime" or "rape" or "assault" or "sexual harassment" or "sexual coercion" or "forced sex" or "sexual slavery" or "sex work" or battered or "sexual exploitation" or "human trafficking" or "restricted physical integrity" or patriarch* or "bodily integrity")).tw,id.	17059
76	education/	33156
77	((gender or wom#n or women's or feminine* or female* or reproductive) adj3 (educat* or "school enrollment" or "school attendance" or literacy or (access adj2 information))).tw,id.	16184
78	employment status/ or occupations/ or unemployment/	26525
79	((gender or women or women's or feminine or female* or reproductive) adj3 (wage? or employ* or "labo?r force" or work* or "pay gap" or underemploy* or unemploy* or	30647

	microfinance or microcredit or "occupational segregation" or economic or poverty or "basic needs" or financial or (cash adj2 transfer*) or voucher? or (saving? adj2 group?) or (self help adj2 group) or income)).tw,id.	
80	life expectancy/ or birth rate/	3473
81	((gender or women or women's or feminine* or female* or reproductive) adj3 ("life expectancy" or "fertility rate" or "birth rate" or "son preference" or (sex ratio adj2 birth) or (fertility adj3 preference*))).tw,id.	290
82	marriage/ or ownership/	11677
83	((gender or women or women's or feminine* or female* or reproductive) adj3 (security or land or inheritance or marriage or "early marriage" or "parental authority")).tw,id.	1832
84	politics/ or political participation/	17906
85	((gender or wom#n or women's or feminine* or female* or reproductive) adj3 (politic* or "civil libert*" or (access adj2 public space) or "parliamentary seats")).tw,id.	2457
86	((gender or wom#n or women's or feminine* or female* or reproductive) adj3 (equal* or inequalit* or unequal* or justice* or injustice* or equit* or inequit* or disparit* or parit*)).tw,id.	10898
87	((gender or women or women's or feminine* or female* or reproductive) adj3 (discriminat* or bias* or blind* or gap? or barrier?)).tw,id.	10523
88	sex roles/ or social norms/	24627
89	((gender or wom#n or women's or feminine* or female* or reproductive) adj3 (role? or norm? or dynamic?)).tw,id.	25528
90	((gender or wom#n or women's or feminine* or female* or reproductive) adj3 transformat*).tw,id.	632
91	human sex differences/	109352
92	((gender or wom#n or women's or feminine* or female* or reproductive) adj3 (difference? or stereotype? or perspective? or intersect* or sensiti#ation)).tw,id.	63011
93	(gender adj3 traditional).tw,id.	2276
94	socialization/	10831
95	(gender adj3 (sociali#ation or identity)).tw,id.	9404
96	(gender adj3 (relat* or specific or responsive or prescription* or dimension*)).tw,id.	23390
97	attitudes/ or sex role attitudes/	35996
98	(Attitude? adj5 (violence or "wife beating" or "refusing sex")).tw,id.	1448

99	reproductive health/	2992
100	self-concept/ or self-confidence/ or self-esteem/ or self-regard/	68688
101	autonomy/ or choice behavior/	25138
102	((gender or wom#n or women's or female or girl or reproductive) adj3 empower*).tw,id.	2018
103	(empower* adj3 (social or economic or health or legal or financial or political or psychological)).tw,id.	2610
104	("safe space?" or "female friendly space?").tw,id.	624
105	((wom#n or women's or girl? or female) adj5 ("safe space?" or "friendly space?")).tw,id.	27
106	decision making/	70591
107	((gender or wom#n or women's or female or girl) adj3 (disempower* or autonomy or participation or status or preference* or power or agency or right* or feminism or authorit* or control* or leadership or choice or voice or "critical consciousness" or working or mobility or freedom or ownership or assets or (contribution adj2 family income) or "reproductive rights" or movement or training or "labo?r participation" or "self esteem" or "collective action*" or "self-efficacy" or "self efficacy" or "skill* building" or "self respect" or "self worth" or "solidarity")).tw,id.	53400
108	((wom#n or women's or female) adj5 ("decision making" or decision-making)).tw,id.	1507
109	exp socioeconomic status/ or economic security/ or "income (economic)"/ or poverty/	63777
110	exp Family Size/	1143
111	(decision making adj3 (power or joint or domestic or intrahousehold or "intra household" or influence)).tw,id.	2901
112	help seeking behavior/ or leadership/ or interpersonal communication/	51303
113	health care delivery/ or health care access/ or "quality of care"/ or health disparities/	38951
114	(health adj3 access).tw,id.	7492
115	health knowledge/ or health attitudes/ or health behavior/	39944
116	or/1-34	167638
117	or/45-50	717553
118	or/70-115	735970
119	or/51-69	1642936
120	44 and 116 and 117 and 118 and 119	1483

Database(s): Scopus

Search Strategy: yielded 2,504 results

((TITLE-ABS-KEY ("protected village" OR "refugee camp") OR TITLE-ABS-KEY (camp) W/3 (refugee OR transit OR displace* OR temporary OR informal)) OR (TITLE-ABS-KEY (settlement W/3 (temporary OR informal)) OR TITLE-ABS-KEY ({disasters} OR {emergencies} OR {mass casualty incidents} OR {natural disasters}) OR (TITLE-ABS-KEY (disasters OR emergencies OR "mass casualty incidents" OR "natural disasters")) OR (TITLE-ABS-KEY ("disaster victims")) OR (TITLE-ABS-KEY ((disaster OR emergenc*) W/2 victim*)) OR (TITLE-ABS-KEY ((disaster* OR catastrophe*) W/5 (environment* OR human OR manmade OR "man made" OR nature OR natural OR weather)))) OR ((TITLE-ABS-KEY ({mass casualty} OR {mass casualties} OR {mass fatalities} OR {mass fatality}))) OR (TITLE-ABS-KEY (crisis OR crises OR conflict W/3 affected)) OR ((TITLE-ABS-KEY ("warfare and armed conflicts" OR {armed conflict*} OR warfare OR {ethnic cleansing} OR genocide OR holocaust OR "war exposure")) OR (TITLE-ABS-KEY ({afghan campaign} OR {gulf war} OR {iraq war} OR "war time" OR "wartime" OR "war torn" OR "war affected" OR {insurgency} OR {intra conflict}))) OR ((TITLE-ABS-KEY (fragile W/2 (state* OR countr* OR nation* OR situation* OR setting*))) OR (TITLE-ABS-KEY ("Post conflict" OR "postconflict" OR "post war" OR "post conflict setting" OR {peacebuilding} OR peacekeeping)) OR (TITLE-ABS-KEY (war W/2 related)) OR (TITLE-ABS-KEY ("militant group" OR "militant organization" OR "militant organisation" OR militia OR combatant OR rebel*)) OR (TITLE-ABS-KEY ({disaster medicine} OR "disaster outbreak" OR {epidemic} OR "disease outbreak")) OR (TITLE-ABS-KEY ("Emergency Medical Services"))) OR ((((TITLE-ABS-KEY (famine OR starvation)) OR (TITLE-ABS-KEY (avalanche OR cyclone OR drought OR earthquake OR flood* OR hurricane OR landslide OR {land slide} OR {land slides} OR mudslide OR {mud slide} OR {mud slides} OR storm OR tornado* OR tsunami OR typhoon OR volcanic OR {volcanic eruption} OR {volcanic ash} OR {volcanic gases} OR rubble)) OR (TITLE-ABS-KEY (evacuee OR refugee OR squatter OR transients OR {asylum seeker})) OR (TITLE-ABS-KEY ((rescue OR relief OR aid) W/3 (plan OR activity OR activities OR agency OR agencies))) OR (TITLE-ABS-KEY ("aid plan" OR "aid work" OR "relief plan" OR "relief work" OR "rescue plan" OR "rescue work")) OR (TITLE-ABS-KEY ((staff OR worker) W/3 (relief OR aid OR rescue))) OR (TITLE-ABS-KEY ("humanitarian assistance")) OR (TITLE-ABS-KEY (humanitarian W/3 (aid OR response OR relief OR crisis OR crises OR emergency OR emergencies OR disaster OR disasters))) OR (TITLE-ABS-KEY (humanitarianism OR altruism)) OR (TITLE-ABS-KEY ({displaced children} OR {displaced child} OR {displaced families} OR {displaced family} OR {displaced individuals} OR {displaced internally} OR {displaced men} OR {displaced people} OR {displaced peoples} OR {displaced person} OR {displaced persons} OR {displaced population} OR {displaced populations} OR {displaced women} OR {displaced adolescent} OR {displaced adolescents} OR {forced displacement} OR {forced

displacements} OR {forcibl* displace*} OR {internal displaced} OR {internal displacement} OR {internally displaced} OR {population displaced} OR {population displacement} OR {forced migration} OR {migrant*}))))) OR ((TITLE-ABS-KEY (crisis W/5 (environ* OR human OR manmade OR "man made" OR nature OR natural OR weather OR setting*)))) OR ((TITLE-ABS-KEY ((armed OR zone OR political OR civil OR setting*) W/3 (conflict OR conflicts OR attack OR attacks OR war OR wars OR "no fly")))) OR ((TITLE-ABS-KEY (emergency W/5 (environment* OR human OR manmade OR "man made" OR nature OR natural OR weather OR complex))))) AND ((((TITLE-ABS-KEY ("Developing Countries")) OR (TITLE-ABS-KEY (africa OR asia OR caribbean OR {West Indies} OR {South America} OR {Latin America} OR {Central America})) OR (TITLE-ABS-KEY (fiji OR gabon OR {Gabonese Republic} OR gambia OR gaza OR georgia OR {Georgia Republic} OR {Georgian Republic} OR ghana OR {Gold Coast} OR grenada OR guatemala OR guinea OR bissau OR guiana OR guyana OR haiti OR hondu ras OR india OR maldives OR indonesia OR iran OR iraq OR jamaica OR jordan OR kazakhstan O R kazakh OR kenya OR kiribati OR korea OR {Democratic People's Republic of Korea} OR kosovo OR kyrgyzstan OR kirghizia OR {Kyrgyz Republic} OR kirghiz OR kirgizstan)) OR (TITLE-ABS-KEY ({Lao PDR} OR laos OR lebanon OR lesotho OR basutoland OR liberia OR libya OR macedonia OR mad agascar OR {Malagasy Republic} OR malaysia OR malaya OR malay OR sabah OR sarawak OR malawi OR nyasaland OR mali OR {Marshall Islands} OR mauritania OR mauritius OR {Agalega Islands} OR mexico OR micronesia OR {Middle East} OR moldova OR moldovia OR moldovian OR mongolia OR montenegro OR morocco OR ifni OR mozambique OR myanmar OR myanma OR burma OR namibia OR nauru OR nepal OR nicara gua OR niger OR nigeria OR pakistan OR {Papua New Guinea} OR palestine OR paraguay OR peru OR philippines OR philipines OR phillipines OR phillip pines OR romania OR rumania OR roumania OR russia OR russian OR rwanda OR ruanda OR {Sai nt Lucia} OR {St Lucia})) OR (TITLE-ABS-KEY ({Saint Vincent} OR {St Vincent} OR grenadines OR samoa OR {Samoan Islands} OR {Navigator Island} OR {Navigator Islands} OR {Sao Tome} OR senegal OR serbia OR montenegro OR {sierra leone} OR {sri lanka} OR ceylon OR {solomon islands} OR somalia OR {south Africa} OR sudan OR {South Sudan} OR suriname OR surinam OR swaziland OR eswatini OR syria OR {Syrian Arab Republic} OR tajikistan OR tadzhikistan OR tadjikistan OR tadzhik OR tanzania OR thailand OR tog o OR {Togolese Republic} OR tonga OR tunisia OR turkey OR turkmenistan OR turkmen OR tuvalu OR uganda OR ukraine OR ussr OR {Soviet Union} OR {Union of Soviet Socialist Republics} OR uzbekistan OR uzbek OR vanuatu OR {New Hebrides} OR venezuela OR vietnam OR {Viet Nam} OR {West Bank} OR yemen OR yugoslavia OR zambia OR zimbabwe OR rhodesia)) OR (TITLE-ABS-KEY (afghanistan OR albania OR algeria OR {American Samoa} OR angola OR armenia OR armenian OR azerbaijan OR bangladesh OR benin OR byelarus OR byelorussian OR belarus OR belorussian OR belorussia OR belize OR bhutan OR bolivia OR b

osnia OR herzegovina OR hercegovina OR botswana OR brasil OR brazil OR bulgaria OR {Burkina Faso} OR {Burkina Fasso} OR {Upper Volta} OR burundi OR urundi OR cambodia OR {Khmer Republic} OR kampuchea OR cameroon OR cameroons OR cameron OR camerons OR cameroun OR {Cape Verde} OR {Cabo Verde} OR {Central African Republic} OR chad OR china OR colombia OR comoros OR {Comoro Islands} OR comores OR mayotte OR congo OR zaire OR {Costa Rica} OR {Cote d'Ivoire} OR {Ivory Coast} OR cuba OR djibouti OR {French Somaliland} OR dominica OR {Dominican Republic} OR {East Timor} OR {East Timur} OR {Timor Leste} OR ecuador OR egypt OR {United Arab Republic} OR {El Salvador} OR {Equatorial Guinea} OR eritrea OR ethiopia)))) OR (((TITLE-ABS-KEY (lmic OR lmics OR {third world} OR {lami countr*})) OR (TITLE-ABS-KEY ("transitional countr*")))) OR ((TITLE-ABS-KEY ((developing OR "less* developed" OR "under developed" OR underdeveloped OR "middle income" OR "low* income" OR "underserved" OR "under served" OR deprived OR poor) W/2 ("countr*" OR "nation?" OR "population?" OR world)))) OR ((TITLE-ABS-KEY ((developing OR "less* developed" OR "under developed" OR underdeveloped OR "middle income" OR "low* income") W/2 economy))) OR ((TITLE-ABS-KEY (low* W/2 (gdp OR gnp OR "gross domestic" OR "gross national"))) OR (TITLE-ABS-KEY (low W/3 middle W/3 ("countr*" OR "nation*"))))) AND (((TITLE-ABS-KEY (gender OR "interpersonal relations" OR "human rights" OR "women's rights" OR "empowerment" OR "women's empowerment" OR feminism OR "gender identity" OR "gender bias" OR "sex role" OR "social norm" OR "reproductive rights")) OR (TITLE-ABS-KEY ((gender OR wom?n OR women's OR feminine OR female* OR reproductive) W/5 (violence OR education OR school OR literacy OR access OR employ* OR occupation OR unemploy* OR wage OR "labo*r force" OR work OR "pay gap" OR underemploy* OR microfinance OR microcredit OR "occupational segregation" OR economic OR poverty OR "basic needs" OR financial OR (cash W/2 transfer) OR voucher* OR (saving* W/2 group) OR (self AND help W/2 group) OR income))) OR (TITLE-ABS-KEY ((gender OR wom?n OR women's OR feminine OR female* OR reproductive) W/5 ("life expectancy" OR "birth rate" OR "fertility preference" OR "sex ratio at birth" OR "son preference" OR security OR land OR inheritance OR marriage OR "early marriage" OR "parental authority" OR "forced marriage" OR ownership OR politic* OR "civil liberty" OR "civil liberties" OR "parliamentary seats")))) OR ((TITLE-ABS-KEY ((gender OR wom?n OR women's OR feminine OR female* OR reproductive) W/5 (equal* OR inequalit* OR unequal* OR justice* OR injustice* OR equit* OR inequit* OR disparit* OR parit* OR discriminat* OR bias OR blind OR gap OR barrier OR role OR norm OR dynamic* OR transformat* OR difference OR stereotyp* OR perspective OR intersect* OR sensiti?ation OR traditional OR sociali?ation OR identity OR relat* OR specific OR responsive OR prescription OR dimension))) OR (TITLE-ABS-KEY ((gender OR wom?n OR women's OR feminine OR female* OR reproductive) W/5 (victim* OR abus* OR "sex* crime" OR "rape" OR "assault" OR "sexual harassment" OR "sexual

coercion" OR "forced sex" OR "sexual slavery" OR "sex work" OR battered OR "sexual exploitation" OR "human trafficking" OR "restricted physical integrity" OR patriarch* OR "bodily integrity" OR battered)))) OR ((TITLE-ABS-KEY ((gender OR wom?n OR women's OR feminine OR female* OR reproductive) W/5 (disempower* OR autonomy OR participation OR status OR preference OR power OR agency OR right* OR authority OR control* OR leadership OR choice OR voice OR "critical consciousness" OR working OR mobility OR freedom OR movement OR ownership OR assets OR "reproductive rights" OR training OR "labo*r participation" OR "self esteem" OR "self concept" OR "collective action" OR "self efficacy" OR "skill* building" OR "self respect" OR "self worth" OR solidarity))) OR (TITLE-ABS-KEY ((attitude*) W/5 (violence OR "wife beating" OR "refusing sex"))) OR (TITLE-ABS-KEY ((empower*) W/3 (social OR economic OR health OR legal OR financial OR political OR psychological))) OR (TITLE-ABS-KEY ((gender OR wom?n OR women's OR feminine OR female* OR reproductive) W/5 ("decision making" OR household OR "household income" OR poverty OR "safe space*" OR "female friendly space*" OR socioeconomic OR "social status" OR "education* status" OR "family size")))) OR ((TITLE-ABS-KEY ("decision making" W/3 (power OR joint OR domestic OR intrahousehold OR "intra household" OR influence))) OR (TITLE-ABS-KEY ((gender OR wom?n OR women's OR feminine OR female* OR reproductive) W/5 (health OR maternal OR "help seeking behaviour" OR "health care delivery" OR "health care access" OR "health care quality" OR "health equity"))))) AND ((((TITLE-ABS-KEY ("Health status indicators")) OR (TITLE-ABS-KEY (indicator* OR "health indicator" OR evaluation OR tool* OR assessment* OR methodolog* OR standards)) OR (TITLE-ABS-KEY (monitor* OR surveillance OR screening)) OR (TITLE-ABS-KEY ({rapid counting} OR {aerial surveillance} OR {flow monitoring} OR {enumeration} OR {reproductive health assessment toolkit})) OR (TITLE-ABS-KEY ((rapid OR needs OR risk OR outcome) W/2 (assessment OR evaluation))) OR (TITLE-ABS-KEY ("data collection tool*" OR "data source*" OR "data collection" OR "needs assessment" OR "questionnaire" OR "health survey" OR "health care survey")) OR (TITLE-ABS-KEY (scale* OR survey*)) OR (TITLE-ABS-KEY (index OR indices)) OR (TITLE-ABS-KEY ({minimum initial service package} OR misp)) OR (TITLE-ABS-KEY (measured OR measurement OR metric*)) OR (TITLE-ABS-KEY (outcome* W/3 (health OR measur* OR assess* OR scor* OR index OR indices OR scale* OR monitor*))) OR (TITLE-ABS-KEY (research))) OR (TITLE-ABS-KEY (gender W/3 (analys* OR analy?ing OR mainstreaming OR marker OR "risk assessment"))) OR (TITLE-ABS-KEY ("gender empowerment measure" OR "gender related development index" OR "gender development index" OR "gender gap index" OR "global gender gap index" OR "global gender gap report" OR "gender intensity measure" OR "gender inequality index" OR "gender equity index" OR "gender parity index" OR "human development index")) OR (TITLE-ABS-KEY ("social institutions gender index" OR "survey based women's empowerment index" OR "women's empowerment in agriculture index" OR "demographic and health surveys" OR "african gender and development index" OR "gender status index" OR "african women's

progress scoreboard" OR "gender mainstreaming scorecard" OR "gender equality action plan" OR "millenium development goals" OR "sustainable development goals")))) AND (((TITLE-ABS-KEY (adolescent* OR teen* OR youth OR "young adult" OR girl* OR "young wom?n" OR "young girl*")) OR (TITLE-ABS-KEY ("Pregnant Wom?n" OR wom?n)) OR (TITLE-ABS-KEY (mother)) OR (TITLE-ABS-KEY (women W/2 reproductive W/2 age)))) AND NOT INDEX (medline) AND (LIMIT-TO (PUBYEAR , 2020) OR LIMIT-TO (PUBYEAR , 2019) OR LIMIT-TO (PUBYEAR , 2018) OR LIMIT-TO (PUBYEAR , 2017) OR LIMIT-TO (PUBYEAR , 2016) OR LIMIT-TO (PUBYEAR , 2015) OR LIMIT-TO (PUBYEAR , 2014) OR LIMIT-TO (PUBYEAR , 2013) OR LIMIT-TO (PUBYEAR , 2012) OR LIMIT-TO (PUBYEAR , 2011) OR LIMIT-TO (PUBYEAR , 2010) OR LIMIT-TO (PUBYEAR , 2009) OR LIMIT-TO (PUBYEAR , 2008) OR LIMIT-TO (PUBYEAR , 2007) OR LIMIT-TO (PUBYEAR , 2006) OR LIMIT-TO (PUBYEAR , 2005) OR LIMIT-TO (PUBYEAR , 2004))
