# Supplementary material for: Tools for measuring gender equality and women’s empowerment (GEWE) indicators in humanitarian settings
Source: Confl Health. 2021 May 17;15:39. doi: 10.1186/s13031-021-00373-6 (PMC8127307; doi:10.1186/s13031-021-00373-6)
Supplement: Supplementary file 2 — Additional file 2. Inclusion and exclusion criteria. This file includes the inclusion and exclusion criteria for the review. [file 13031_2021_373_MOESM2_ESM.pdf]

Additional file 2: Inclusion and Exclusion Criteria

	**Inclusion Criteria**	**Exclusion Criteria**
**Population of Interest**	Adolescent girls (10 – 19 years), women of reproductive age (WRA) (15-49 years), older women (49+ years)	Males (unless the outcomes reported are on women), very young female children (<10 years), military personnel or groups other than beneficiaries (i.e. health workers, NGO workers)
**Intervention**	Interventions to improve gender equality and/or women's empowerment	Interventions unrelated to gender equality or women's empowerment
	SRHR interventions with indicators that may be used to measure gender equality or women's empowerment (e.g., GBV)	
	Maternal health interventions with indicators that may be used to measure gender equality or women's empowerment	
**Comparison**	Not necessary, but comparison groups (ex: men and boys) will be noted where applicable	
**Outcomes of Interest**	Quantitative indicators (with reported outcomes), and their corresponding measurement tools, data sources, and data collection methodologies	• Quantitative indicators that do not report outcomes, or provide a methodology for how the data were collected<br>• Qualitative outcomes<br>• Quantitative outcomes based on mathematical modelling of descriptive statistics (i.e. regression coefficients, odds ratios, relative risk etc.)<br>• Health systems data with no indicator denominator
**Situation/ Setting**	Low- and middle-income countries (LMICs) that experienced or have experienced (within 5 years of data collection) one or more of the following humanitarian crises: conflict, epidemic (i.e. Ebola or Zika), or natural disaster (affecting a population greater than 1000)	• High-income countries<br>• High-income countries hosting refugee/displaced populations
		LMICs where data was collected more than 5 years after a humanitarian crisis(es) ended
	LMICs hosting displaced population(s) affected by one or more of the humanitarian crises of interest (text must specifically mention a refugee/displaced population living in a low/middle income country if the host country is not experiencing a conflict/natural disaster)	• LMICs that are not affected by one or more of the humanitarian crises of interest, nor mention hosting a refugee or displaced population from other LMICs<br>• LMICs that have only experienced HIV as an epidemic
**Type of Evidence**	Reports, indexed literature, reviews, conference or other abstracts with details about the methodology	Blogs, web pages, dissertations, pamphlets/fact sheets with no methodology reported, financial reports/programming budgets
**Time**	Any study published from 2004 — present	Any study published before 2004
**Language**	English	Other languages with no translation available
