# Supplementary material for: Tools for measuring gender equality and women’s empowerment (GEWE) indicators in humanitarian settings
Source: Confl Health. 2021 May 17;15:39. doi: 10.1186/s13031-021-00373-6 (PMC8127307; doi:10.1186/s13031-021-00373-6)
Supplement: Supplementary file 3 — Additional file 3. Machine learning tool. This file explains the machine learning tool we used to conduct the title/abstract screening phase of the review. [file 13031_2021_373_MOESM3_ESM.pdf]

## Additional file 3: Machine Learning Tool

We developed a neural network classifier using the deep learning library fastai in python (1). The algorithm classified titles and abstracts from the index literature search according to a training dataset, and the short and long-term dependencies of the words they contain. From the 19,879 total titles and abstracts identified during the search strategy, two sets of 1000 articles were randomly selected, using the base random number generator in R, to make up the training and validation datasets. The inclusion rate determined by these 2,000 randomly selected articles, was 4.8% (95%CI: 3.8% to 5.7%). Therefore, the corresponding range of total included titles and abstracts based on this rate was estimated to be between 756 and 1,133.

The algorithm was trained on a batch of 1,000 screened titles and abstracts and was used to predict the probability of inclusion for the leftover abstracts. The highest ranked abstracts were screened in duplicate first and, at the end of each screening session, were added to the training dataset and the algorithm was retrained. This process was repeated every day. Abstract screening ranged from 309 to 700 abstracts a day, with a median of 500. The inclusion rate per batch ranged from 33.98% on the second batch to 1.00% on the last batch.

Counting the 14 daily batches of ranked abstracts and the randomly selected training and validation datasets, we included 1,106 of the 8,948 titles and abstracts we reviewed (for an inclusion rate of 12.36%). According to our validation dataset, at a sensitivity of 95%, we reached a specificity of 75.56%, an accuracy of 76.4% and a precision of 14.29%. This corresponds to a Work Saved Over Random Sampling at 95% sensitivity of 58.4%. (Figure 1). Employing machine learning software was used as it increased the efficiency of the screening process.

**Figure 1: Predicted probability of inclusion of abstracts in the validation dataset (n=1000)**

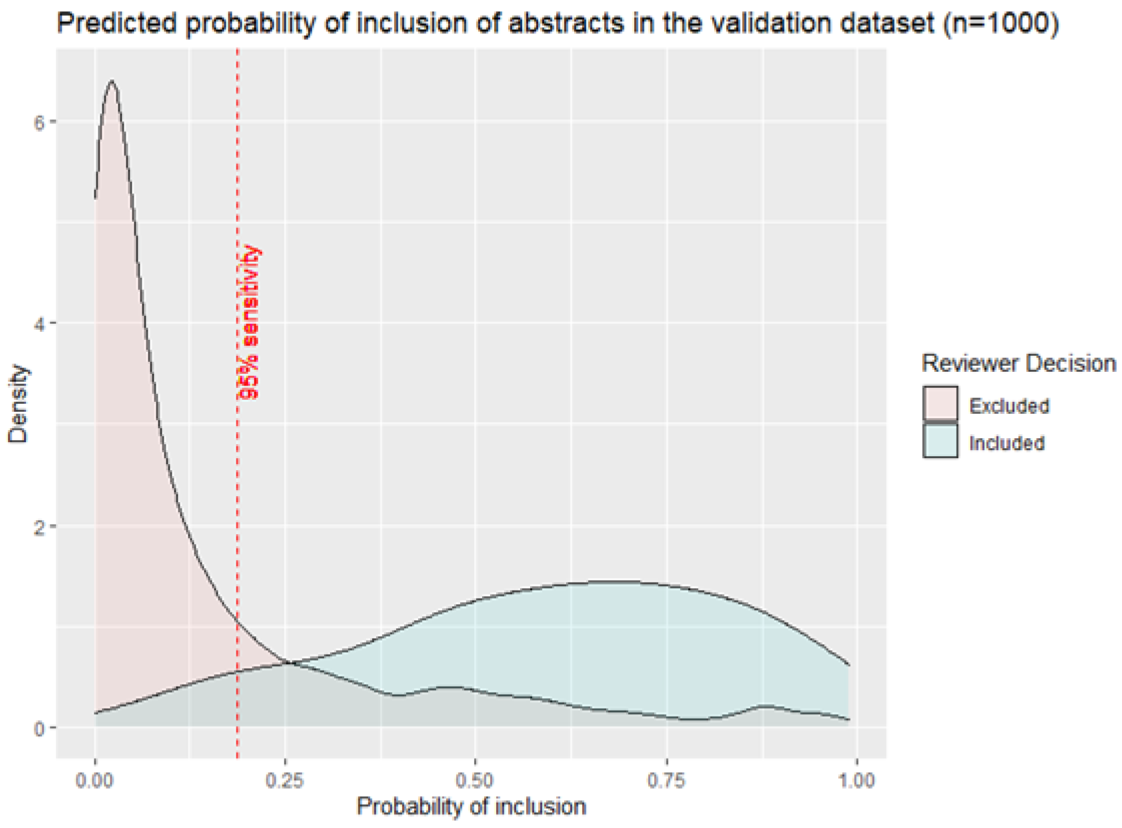


1. Howard J, Gugger S. Fastai: A Layered API for Deep Learning. Information. 2020;11(2):108.
