# Supplementary material for: Tools for measuring gender equality and women’s empowerment (GEWE) indicators in humanitarian settings
Source: Confl Health. 2021 May 17;15:39. doi: 10.1186/s13031-021-00373-6 (PMC8127307; doi:10.1186/s13031-021-00373-6)
Supplement: Supplementary file 4 — Additional file 4. Data collection toolkits and surveys. This file explains the data collection tools used by studies included in this review. [file 13031_2021_373_MOESM4_ESM.pdf]

Additional file 4: Data Collection Toolkits and Surveys

Measurement Tool (Standard Name)	Organization/ Author	Sex Disaggregated (Y/N/Mixed)	Toolkit Topic/Domain	Population Type	Humanitarian Specific (Y/N)	# of Studies
Adolescent Sexual & Reproductive Health Assessment Toolkit for Humanitarian Settings	Ivanova	N	Economic, Health, Social/Cultural	Adolescent girls	Y	1
Assessment Screen to Identify Survivors Toolkit for Gender-Based Violence (ASIST-GBV)	Vu	N	Economic, Health, Social/Cultural	Survivors of violence	Y	1
CDC Reproductive Health Assessment Toolkit for Conflict-Affected Women	Bell, Falb, Hynes, Ivanova, Okanlawon, Seyife, Sipsma	Mixed	Economic, Health, Human Development, Security and Justice, Social Cultural	Adolescent girls, Adolescents, Pregnant & postpartum Women, WRA, Young adults	Y	8
Child War Trauma Questionnaire (CWTQ)	Klasen	Y	Security and Justice, Social/Cultural	Adolescents	Y	1
Communities Care Toolkit*	Read-Hamilton	N	Social/Cultural	Adults	Y	1
Diriswanaag Livelihood Final Evaluation Questionnaire	Care International	Mixed	Economic, Human Development, Security and Justice, Social/Cultural	Adults	Y	1
Gender Needs Assessment (GNA) Survey*	UNIFEM	N	Economic, Health, Human development, Leadership, Security and Justice, Social/Cultural	Adolescent girls, Girls, Women	Y	1
Gender-Based Violence Tools Manual in Conflict-Affected Settings	Usta	N	Health, Pychological, Security and Justice, Social/Cultural	Women	Y	1
HIV Behavioral Surveillance Surveys	Harrison, UNHCR	Mixed	Economic, Health, Human Development,	Adults, Women, WRA	Y	2

			Security and Justice, Social/Cultural			
Human Rights and Sexual Violence Survey*	Amowitz	N	Economic, Health, Security and Justice, Social/Cultural	WRA	Y	2
Inclusive Disaster Risk Management Framework and Toolkit	Thapa	Y	Social/cultural	Adults	Y	1
Knowledge, Attitudes and Practices Survey: GBV and Child Protection Issues*	ALNAP, Queen Zein Al-Sharaf Institute for Development (ZENID)	Mixed	Economic, Health, Human Development, Security and Justice, Social/Cultural	Adolescents, Adults, Children	Y	1
Knowledge, Attitudes, and Practices Survey	ALNAP, Care International	Mixed	Economic, Health, Human Development, Leadership, Security and Justice, Social/Cultural	Adults, Women, WRA	Y	2
LSHTM Violence and Health Among Women Asylum Seekers	Hossain, Shuman	Mixed	Health, Human Development, Psychological, Social/Cultural	Adults, Women	Y	2
Millennium Development Indicators of Education, Employment & Gender Equality*	UNHCR	Y	Economic, Human Development	Adults	Y	1
Neighbourhood Method*	Parcesepe	N	Health, Security and Justice, Social/Cultural	Women	Y	1
Personal Network Analysis Survey*	Faas	Y	Economic, Human Development, Psychological	Adults	Y	1
Sisters of Success Liberia Survey	IRC	N	Economic, Psychological, Social/Cultural	Adolescent girls, Adults	Y	1
Survey of War-Affected Youth (SWAY)*	Patel	Mixed	Social/Cultural	Young adults, Young women	Y	1

Women of Reproductive Age = WRA
