# Supplementary material for: Tools for measuring gender equality and women’s empowerment (GEWE) indicators in humanitarian settings
Source: Confl Health. 2021 May 17;15:39. doi: 10.1186/s13031-021-00373-6 (PMC8127307; doi:10.1186/s13031-021-00373-6)
Supplement: Supplementary file 5 — Additional file 5. Measurement of Sexual and Reproductive Health and Rights, Gender Equality, and Women’s Empowerment in Humanitarian Settings. This link leads to the interactive Tableau dashboard. https://public.tableau.com/profile/humairanakhuda#!/vizhome/SRHRGEWEScopingReviewStory_Final_Nov16/SRHRGEWEStory. [file 13031_2021_373_MOESM5_ESM.pdf]

Additional File 5: Link to our Tableau Dashboard

Interactive Tableau Dashboard: Measurement of Sexual and Reproductive Health and Rights, Gender Equality, and Women's Empowerment in Humanitarian Settings.

https://public.tableau.com/profile/humairanakhuda#!/vizhome/SRHRGEWEScopingReviewStory_Final_Nov16/SRHRGEWEStory
