# Supplementary material for: Tools for measuring gender equality and women’s empowerment (GEWE) indicators in humanitarian settings
Source: Confl Health. 2021 May 17;15:39. doi: 10.1186/s13031-021-00373-6 (PMC8127307; doi:10.1186/s13031-021-00373-6)
Supplement: Supplementary file 7 — Additional file 7. Studies by disaster type. This file contains a list of all publications by disaster type. [file 13031_2021_373_MOESM7_ESM.pdf]

Additional File 7: Studies by Disaster Type

Type of Disaster	Number of studies by Disaster Type	Title	First Author	Publication Year	Country
Drought	4	Social capital and disaster preparedness in Oromia, Ethiopia: An evaluation of the Women Empowered approach	Story	2018	Ethiopia
		Consolidated Gender Analysis for the Ethiopian Drought Response	Oxfam, CARE	2016	Ethiopia
		Diriswanaag livelihoods final evaluation- final report 29 9 2013	Wanyama	2013	Somalia
		Emergency cash-first response evaluation	Tirivayi	2016	Zimbabwe
Earthquake	8	Violence and abuse of internally displaced women survivors of the 2010 Haiti earthquake	Campbell	2016	Haiti
		Mortality, crime and access to basic needs before and after the Haiti earthquake: a random survey of Port-au-Prince households	Kolbe	2010	Haiti
		A psycho-educational HIV/STI prevention intervention for internally displaced women in Leogane, Haiti: results from a non-randomized cohort pilot study	Logie	2014	Haiti
		Sentinel events predicting later unwanted sex among girls: A national survey in Haiti, 2017	Sumner	2015	Haiti
		The experience of violence against children in domestic servitude in Haiti: Results from the Violence Against Children Survey, Haiti 2012	Gilbert	2018	Haiti
		Picking up the pieces: Women's health needs assessment, Fond Parisien Region, Haiti	Hudson	2010	Haiti
		Does it take a village? Fostering gender equity among early adolescents in Nepal	Lundgren	2018	Nepal
		Gender inclusiveness in disaster risk governance for sustainable recovery of 2015 Gorkha Earthquake, Nepal	Thapa	2019	Nepal
Flood	3	Pakistan Floods 2010: Rapid Gender Needs Assessment of Flood Affected Communities	UN	NR	Pakistan
		Restoring Livelihoods After Floods: Gender-sensitive response and community-owned recovery in Pakistan	Jeffrey	2012	Pakistan
		Consolidated Gender Analysis for Disaster Response in Pakistan	NR	2017	Pakistan
Tsunami	1	Gender and Changes in Tsunami-Affected Villages in Nanggroe Aceh Darussalam province	Minza	2005	Indonesia
Typhoon	3	Philippines-haiyan-care baseline study	NR	2016	Philippines

		Typhoon haiyan reconstruction assistance project midterm	NR	2017	Philippines
		TYPHOON HAIYAN RESPONSE PROGRAM Final Evaluation	NR	2017	Philippines
Volcanic	1	Gendered access to formal and informal resources in post-disaster development in the Ecuadorian Andes	Faas	2014	Ecuador
