# Supplementary material for: Tools for measuring gender equality and women’s empowerment (GEWE) indicators in humanitarian settings
Source: Confl Health. 2021 May 17;15:39. doi: 10.1186/s13031-021-00373-6 (PMC8127307; doi:10.1186/s13031-021-00373-6)
Supplement: Supplementary file 8 — Additional file 8. Indicator types and subtypes. This file contains the calculated frequency of each indicator type and subtype organized by domain. [file 13031_2021_373_MOESM8_ESM.pdf]

Additional file 8: Indicator Types and Subtypes

	Counted once per study	Publication Type (Counted subtype once per study) Peer-Review	Non Peer-Review	Counted once per country
**Economic**				
**Access to Finance**	**22**	**6**	**16**	**14**
Able to pay for education	1	0	1	1
Formal economic support	12	3	9	9
Income-generating activity (IGA) or business	7	0	7	6
Informal economic support	2	0	2	2
Lack of access to finance	4	1	3	4
Savings	5	3	2	5
**Child labour**	**7**	**2**	**5**	**8**
Child labour	7	2	5	8
Income from child labour	1	0	1	1
Unpaid child labour	1	1	0	1
**Economic decision-making in the household**	**19**	**4**	**15**	**15**
Lack of women's economic decision-making in the household	14	3	11	11
Women's economic decision-making in the household	15	1	14	13
Women's involvement in household financial decisions	15	2	13	13
Women's control over her own earnings	3	1	2	3
**Economic violence**	**12**	**6**	**6**	**10**
Experienced economic violence	12	6	6	10
**Employment**	**18**	**3**	**15**	**11**
Access to employment	9	1	8	6
Employment preferences	2	0	2	1
Equality in economic opportunities	3	1	2	2
Lack access to employment	6	1	5	3
Mean employment hours	3	2	1	2
Type of employment	5	0	5	5
**Enterprise management**	**3**	**0**	**3**	**1**
Enterprise experience and linkages	3	0	3	1
Women's economic decision-making	2	0	2	1
Leadership in economic activities	2	0	2	1
**Income**	**19**	**6**	**13**	**12**
Allocation of spending	2	1	1	2

Amount of income	7	3	4	6
Equitable contribution to household expenses	2	0	2	2
Self-perceived wealth	1	1	0	1
Source of income	8	1	7	6
Source of income-dowry	1	0	1	1
Women's income	4	1	3	3
**Transactional sex**	**23**	**19**	**4**	**9**
Age at transactional sex debut	2	2	0	2
Condom use during transactional sex	2	1	1	2
Forced transactional sex	2	2	0	2
Had transactional sex	15	12	3	5
Had transactional sex in the last 12 months	6	5	1	5
Location of transactional sex	2	2	0	1
Transactional sex partner	2	2	0	2
**Unpaid labour**	**18**	**3**	**15**	**12**
Child disciplining responsibilities	2	0	2	2
Childcare responsibilities	2	0	2	3
Forced labour	4	2	2	4
Men's childcare responsibilities	3	0	3	3
Men's domestic responsibilities	7	0	7	7
Mean hours of domestic work	3	1	2	2
Satisfaction with division of household labour	1	0	1	2
Shared domestic responsibilities	6	0	6	5
Unpaid labour	1	0	1	1
Women's childcare responsibilities	4	0	4	4
Women's domestic responsibilities	9	0	9	8
**Women's ownership of assets**	**11**	**3**	**8**	**9**
Household assets	4	0	4	4
Investments	3	0	3	2
Livelihood assets	2	0	2	2
Loss of assets	3	1	2	3
Mean durable assets	1	1	0	1
Ownership of livestock	5	1	4	5
Property ownership	5	0	5	4
**Health**				
**Abortion**	**6**	**5**	**1**	**5**
Lack of abortion decision-making	4	3	1	4
Safe abortion care unavailable	1	1	0	1
Self-induced abortion	1	1	0	1
Support for abortion	1	0	1	1
**Menstrual hygiene management**	**6**	**0**	**6**	**6**

Access to menstrual hygiene management	3	0	3	3
Disposal of menstrual hygiene products	1	0	1	1
Lack of access to menstrual hygiene management	6	0	6	6
**Barriers to accessing health services**	**17**	**9**	**8**	**12**
Barriers to health care: Any	12	5	7	10
Barriers to health care: Availability	2	1	1	2
Barriers to health care: Financial reasons	2	1	1	2
Barriers to health care: Lack decision-making ability	4	2	2	4
Barriers to health care: Not permitted by husband/partner	5	4	1	4
Barriers to health care: Restriction of movement	2	2	0	2
Barriers to health care: Stigma/shame/fear	3	2	1	2
Barriers to health care: Work	2	1	1	2
No barriers to health care	2	2	0	2
**Barriers to contraception use/FP**	**19**	**13**	**6**	**16**
Barriers to contraception use/FP: Clients	2	2	0	1
Barriers to contraception use/FP: Family	4	2	2	4
Barriers to contraception use/FP: Financial reasons	1	1	0	1
Barriers to contraception use/FP: Husband/partner	16	10	6	15
Barriers to contraception use/FP: Other	5	1	4	5
Barriers to contraception use/FP: Religion	9	5	4	9
**Communication about sex, HIV and contraception**	**11**	**11**	**0**	**6**
Communication about contraception	7	7	0	5
Communication about sex	1	1	0	1
HIV disclosure	2	2	0	1
Lack of communication about contraception	1	1	0	1
Sex negotiation	1	1	0	1
Sex work disclosure	1	1	0	1
**Decision-making about SRHR**	**20**	**10**	**10**	**11**
Joint SRHR decision-making	13	8	5	8
Lack of women's SRHR decision-making power	10	5	5	8
Perceptions of SRHR decision-making	7	3	4	6
Women's SRHR decision-making power	14	8	6	8
**Health perceptions**	**8**	**4**	**4**	**9**
Negative health perceptions	7	3	4	8
Positive health perceptions	5	2	3	6
**Partner support**	**2**	**0**	**2**	**3**
Accompanied by partner	1	0	1	2
Not accompanied by partner	2	0	2	3
**Reported GBV to medical personnel**	**16**	**7**	**9**	**15**
Reported GBV at hospital/health provider	7	2	5	7
Sought care for GBV from medical personnel	10	5	5	9

**Share of meals within the household**	**3**	**0**	**3**	**2**
Prioritization of food for family members	3	0	3	2
Share of meals in the household-female prioritization	3	0	3	2
**Survivorship**	**1**	**0**	**1**	**1**
Mortality disparities across genders	1	0	1	1
**Human Development**				
**Access to trainings/consultations**	**17**	**5**	**12**	**11**
Access to information	4	1	3	3
Access to social services/aid	1	1	0	1
Access to trainings	7	0	7	7
Lack of access to information	1	0	1	1
Lack of access to trainings	2	1	1	2
Participation in consultations	2	0	2	2
Perceived need for trainings	4	2	2	4
Received livelihood training	2	0	2	2
**Education & Literacy**	**20**	**9**	**11**	**14**
Access to education	12	4	8	11
Lack of access to education	6	1	5	6
Level of education	5	2	3	5
Literacy	5	2	3	5
Perceived need for education	3	1	2	3
Reason for not attending or dropping out of school	6	3	3	6
**Empowerment**	**17**	**9**	**8**	**15**
Change in HIV/STI knowledge	2	2	0	2
Change in SRHR communication	2	2	0	2
Change in condom and FP use/attitudes	3	3	0	3
Change in confidence/control	7	1	6	6
Change in employment/income	5	3	2	5
Change in gender equitable attitudes	1	1	0	1
Change in informational support	2	2	0	2
Change in sexual risk behaviour	1	1	0	1
Improved business/enterprise	2	0	2	1
Improved living situation	2	1	1	2
Increased social support score	1	1	0	1
Knowledge/skill acquisition	4	1	3	3
Participation in community activities	1	0	1	1
**Access to basic needs/essential infrastructure**	**17**	**3**	**14**	**14**
Access to WASH facilities	4	0	4	4
Access to drinking water	1	0	1	1
Access to other basic needs	5	0	5	5

Access to safe spaces	2	1	1	2
Access to social services/aid	4	1	3	4
Access to telecommunications	1	0	1	1
Awareness of services	3	0	3	5
Lack of access to WASH facilities	2	0	2	2
Lack of access to safe spaces	1	0	1	1
Lack of access to social services/aid	2	0	2	2
Lack of access to telecommunications*	1	0	1	1
Lack of awareness of services	2	0	2	2
Other basic needs unmet	2	1	1	2
**Leadership**				
**Community engagement**	**14**	**2**	**12**	**12**
Community engagement	10	2	8	10
Confidence in their participation	2	0	2	2
Exclusion of women's voices	1	0	1	1
Inclusion of women's voices	1	0	1	1
Lack of community engagement	7	0	7	6
Positive attitudes towards women's engagement	2	0	2	2
**Women's leadership**	**6**	**0**	**6**	**5**
Lack of women's representation in leadership	2	0	2	2
Positive attitudes towards women's leadership	2	0	2	2
Women's representation in leadership	3	0	3	2
**Psychological**				
**Self-esteem**	**10**	**4**	**6**	**9**
Feelings/outlook about future	2	0	2	1
Lack of self-esteem	3	1	2	4
Self-esteem	9	4	5	9
**Social network**	**19**	**11**	**8**	**12**
Community leader	1	0	1	1
Lack of social support	2	1	1	2
Negative family relations	9	4	5	6
Positive family relations	6	2	4	6
Social support	8	4	4	7
Supportive adult	4	3	1	4
Women's friendship	3	2	1	3
**Security and Justice**				
**Abduction**	**20**	**15**	**5**	**7**
Experienced abduction	18	13	5	6
Experienced detainment	5	3	2	5
Pregnancy during captivity	2	1	1	2
**Access to the justice system**	**13**	**3**	**10**	**10**

Access to GBV protection services	5	0	5	5
Access to the justice system	5	1	4	5
Availability of GBV-related services	1	0	1	1
Awareness of GBV protection services	3	0	3	3
Ever incarcerated	1	1	0	1
Lack of access to justice system	3	1	2	3
Legal violence	1	0	1	1
Trust community justice	1	1	0	1
Trust legal system	2	1	1	2
Used community justice system	1	1	0	1
**Laws**	**3**	**0**	**3**	**2**
Awareness of laws that protect women	3	0	3	2
Lack of awareness of protective laws/awareness of discriminatory laws	1	0	1	1
**Location of violence**	**14**	**10**	**4**	**12**
Location: Camp	3	2	1	3
Location: Collecting water	1	1	0	1
Location: Field/Forest	4	3	1	2
Location: Home	12	8	4	11
Location: In public	5	3	2	4
Location: Other	11	9	2	8
Location: Perpetrator's home	2	2	0	2
Location: School	4	3	1	4
Location: Travelling	5	4	1	4
Location: Work	1	0	1	1
**Perceptions of safety**	**18**	**4**	**14**	**15**
Perceived risk/knowledge of human trafficking	2	0	2	2
Safe perceptions	10	3	7	8
Unsafe perceptions	12	1	11	10
Unsafe perceptions of others	1	0	1	1
**Perpetrators of violence**	**49**	**35**	**14**	**20**
Admitted to perpetrating GBV	4	0	4	4
Consequences of violence	2	2	0	2
Number of perpetrators	9	7	2	4
Perpetrator: 10+ years older	2	2	0	1
Perpetrator: Armed men/rebel soldiers/members of militias	17	13	4	9
Perpetrator: Civilians	8	3	5	6
Perpetrator: Ex-partner	3	2	1	3
Perpetrator: Family member	16	12	4	12
Perpetrator: Friend/neighbour/acquaintance	14	11	3	11
Perpetrator: Humanitarian/UN worker	4	0	4	4

Perpetrator: Husband/partner	14	10	4	10
Perpetrator: In-laws	3	1	2	3
Perpetrator: Known	7	6	1	6
Perpetrator: Military/police/soldier	20	12	8	12
Perpetrator: Official	2	1	1	2
Perpetrator: Other	18	11	7	11
Perpetrator: Stranger	7	5	2	5
Perpetrator: Teacher	3	3	0	3
Perpetrator: Unknown	8	6	2	7
**Reported GBV**	**16**	**5**	**11**	**11**
Did not/would not report	4	1	3	4
Reported/would report violence to chief/local leader	4	1	3	3
Reported/would report violence to organization	6	2	4	6
Reported/would report violence to police/authorities	15	5	10	11
Reported/would report violence to religious leader	4	1	3	4
Reported/would report violence to the courts	2	1	1	2
**Sociocultural**				
**Age at first marriage**	**25**	**12**	**13**	**14**
Age at first marriage	13	6	7	10
Child marriage perceptions	7	3	4	6
Child marriage/early marriage	12	5	7	7
**Attitudes towards sex and sexuality**	**16**	**6**	**10**	**14**
Consensual sex	2	0	2	2
FP attitudes	2	2	0	2
Negative FP attitudes	2	2	0	2
Negative contraception attitudes	5	0	5	5
Positive FP attitudes	2	2	0	2
Positive contraception attitudes	4	3	1	3
Premarital/sexuality beliefs	5	1	4	5
Responsibility to prevent pregnancy	5	0	5	5
**Early pregnancy**	**6**	**3**	**3**	**8**
Age at first delivery or pregnancy	4	3	1	4
Early pregnancy	1	0	1	3
Perceptions of early pregnancy	1	0	1	1
**Women's autonomy**	**29**	**14**	**15**	**18**
Experience of autonomy	15	10	5	12
Freedom of movement	8	2	6	6
Lack of women's autonomy	9	5	4	9
Lack of freedom of movement	9	0	9	7

Lack of freedom of movement-mobility only if accompanied	3	0	3	3
Reasons for lack of freedom of movement	1	0	1	1
**Female genital cutting/mutilation & other traditional practices**	**11**	**5**	**6**	**9**
Experienced FGM/circumcision	6	4	2	4
Experienced harmful traditional practices	3	1	2	3
Perceptions of honour killing	2	0	2	3
Perceptions of traditional practices	1	0	1	1
Reason for circumcision	1	0	1	1
**Forced marriage**	**16**	**10**	**6**	**11**
Consented to marriage	3	2	1	3
Experienced forced marriage	11	7	4	9
Perceptions of forced marriage	5	3	2	6
**GBV attitudes**	**33**	**18**	**15**	**19**
GBV attitudes: Negative	22	13	9	15
GBV attitudes: Positive	9	5	4	7
Mean IPV attitudes	3	3	0	3
Perceived prevalence of GBV	6	1	5	6
**Gender equitable attitudes**	**25**	**13**	**12**	**16**
Attitudes towards gender equitable policies/laws	5	2	3	5
Attitudes towards gender equitable policies/laws-GBV	2	0	2	3
Attitudes towards gender equitable policies/laws-women's prioritization	1	0	1	2
Attitudes towards gender equitable policies/laws-men's prioritization	1	0	1	1
Gender beliefs in disaster management	2	1	1	2
Gender inequitable attitudes	4	1	3	5
Gender inequitable attitudes-autonomy	3	2	1	3
Gender inequitable attitudes-domestic roles	8	2	6	8
Gender inequitable attitudes-leadership	4	1	3	4
Gender inequitable attitudes-sexual autonomy	8	5	3	7
Gender inequitable attitudes-work/education	6	3	3	6
Mean gender scores	9	5	4	6
Perceptions of gender equality	2	0	2	3
Positive gender equitable attitudes	6	4	2	7
Positive gender equitable attitudes-autonomy	5	4	1	6
Positive gender equitable attitudes-domestic roles	5	1	4	6
Positive gender equitable attitudes-leadership	3	2	1	4
Positive gender equitable attitudes-work/education	10	5	5	9
**Gender-based violence (GBV)**	**113**	**87**	**26**	**34**

Age at rape	4	2	2	3
Attempted assault/violence	3	2	1	3
Domestic violence	22	16	6	15
Emotional/psychological violence	25	20	5	15
Ever experienced sexual violence	20	14	6	11
Experienced unwanted sexual touching	12	9	3	10
Experienced violence	36	30	6	17
Forced sexual debut	5	5	0	3
Forced to undress	4	2	2	4
Frequency of physical violence	4	1	3	4
Frequency of sexual violence	12	7	5	8
Frequency of violence	8	4	4	7
Human trafficking/torture	1	0	1	1
Intimate partner violence (IPV)	31	23	8	16
Intimate partner violence (IPV): Physical	22	17	5	15
Intimate partner violence (IPV): Psychological/emotional	20	13	7	15
Intimate partner violence (IPV): Sexual	27	18	9	17
Intimate partner violence (IPV): Type of physical IPV	11	8	3	10
Knowledge and understanding of GBV	2	0	2	2
Physical violence	41	33	8	19
Pregnancy as a result of violence	12	9	3	8
Psychological impacts of sexual violence	5	5	0	2
Sexual harassment	9	2	7	8
Sexual violence	55	41	14	26
Sexual violence during childhood	1	1	0	1
Sexual violence-injured during assault	3	2	1	3
Social/community violence	4	2	2	3
Torture	4	4	0	4
Violence resulting in death	1	1	0	1
Violence/abuse during childhood	5	2	3	5
Violence/abuse during pregnancy	5	4	1	3
Witnessed violence	9	5	4	6
**Household decision-making power**	**13**	**3**	**10**	**9**
Lack of women's decision-making power	9	2	7	7
Women's household decision-making power	10	1	9	9
Women's involvement in household decision-making	7	1	6	4
**Marriage norms**	**19**	**6**	**13**	**14**
Bride price payment & marriage expenses	3	0	3	3
Decision-making authority for marriage	10	2	8	9
Disempowering divorce norms	3	2	1	4

Disempowering marriage norms	11	5	6	9
Empowering divorce norms	3	1	2	3
Empowering marriage norms	2	1	1	2
Polygamy	4	0	4	4
**Reported GBV to confidant**	**20**	**8**	**12**	**15**
Confided in community leader	4	1	3	4
Confided in family	11	4	7	10
Confided in friends	7	3	4	7
Confided in husband/partner	5	2	3	5
Confided in teacher	2	2	0	2
Confided/would confide in family or friends	2	0	2	2
Confided/would confide in someone	8	4	4	9
Did not confide in anyone	7	4	3	5
Lack of support post-GBV	1	1	0	1
Post-GBV support	3	1	2	3
**Rights and awareness of LGBTQ+**	**4**	**0**	**4**	**3**
Acceptance of individuals who identify as LGBTQ+	2	0	2	2
Awareness of individuals who identify as LGBTQ+ community members	1	0	1	1
Perceived as a Western import	1	0	1	1
Stigma towards individuals who identify as LGBTQ+ community members	4	0	4	3
**Sexual vulnerabilities**	**19**	**13**	**6**	**9**
Age at first sexual encounter	5	3	2	5
Age of husband/partner	2	1	1	2
Early sexual debut	8	5	3	7
Engaged in high risk sex	2	1	1	2
Forced sexual debut	2	1	1	1
Had pimp or sugar daddy	1	1	0	1
Had sex with an older person	5	5	0	2
Had sex with uniformed personnel	3	3	0	1
Low sexual control	3	1	2	3
Sexual assault was witnessed	4	3	1	1
**Stigma**	**12**	**7**	**5**	**6**
Discriminatory practices	5	2	3	5
Experienced stigma after sexual violence	3	3	0	1
Experienced stigma towards child born from sexual violence	1	1	0	1
HIV stigma	2	1	1	2
Rejected by family/friends after sexual assault	4	4	0	2
Rejected by husband after sexual assault	3	2	1	2
