# Supplementary material for: Tools for measuring gender equality and women’s empowerment (GEWE) indicators in humanitarian settings
Source: Confl Health. 2021 May 17;15:39. doi: 10.1186/s13031-021-00373-6 (PMC8127307; doi:10.1186/s13031-021-00373-6)
Supplement: Supplementary file 6 — Additional file 6. Included studies. This file contains all of the included studies retrieved in both the peer-reviewed and non-peer-reviewed literature searches. [file 13031_2021_373_MOESM6_ESM.pdf]

Additional file 6: Included Studies

Study ID	First Author	Title	Publication Year
1.	Kohli	A Congolese community-based health program for survivors of sexual violence	2012
2.	Ivanova	A cross-sectional mixed-methods study of sexual and reproductive health knowledge, experiences and access to services among refugee adolescent girls in the Nakivale refugee settlement, Uganda	2019
3.	Hynes	A determination of the prevalence of gender-based violence among conflict-affected populations in East Timor	2004
4.	Scott	A mixed-methods assessment of sexual and gender-based violence in eastern Democratic Republic of Congo to inform national and international strategy implementation	2013
5.	Abu Hamad	A promise of tomorrow: the effects of UNHCR and UNICEF cash assistance on Syrian refugees in Jordan	2017
6.	Logie	A psycho-educational HIV/STI prevention intervention for internally displaced women in Leogane, Haiti: results from a non-randomized cohort pilot study	2014
7.	O'Callaghan	A randomized controlled trial of trauma-focused cognitive behavioral therapy for sexually exploited, war-affected Congolese girls	2013
8.	Tanner	A safe place to shine: creating opportunities and raising voices of adolescent girls in humanitarian settings	2017
9.	Plan International	Adolescent Girls in Crisis: Experiences of risk and resilience across three humanitarian settings	2018
10.	Qamar	Adolescent Girls Assessment: Needs, Aspirations, Safety and Access	2015
11.	Khawaja	Agreement between husband and wife reports of domestic violence: evidence from poor refugee communities in Lebanon	2004
12.	Mootz	Armed conflict, alcohol misuse, decision-making, and intimate partner violence among women in Northeastern Uganda: a population level study	2018
13.	Khawaja	Attitudes of men and women towards wife beating: findings from Palestinian refugee camps in Jordan	2007
14.	Odwe	Attitudes towards help-seeking for sexual and gender-based violence in humanitarian settings: the case of Rwamwanja refugee settlement scheme in Uganda	2018
15.	Farzaneh	Baseline study: documenting knowledge, attitudes and behaviours of Somali refugees and the status of family planning services in UNHCR's Ali Addeh site, Djibouti	2011

16.	McCoy	Baseline study: documenting knowledge, attitudes and behaviours of Somali refugees and the status of family planning services in UNHCR’s operation in Nairobi, Kenya	2011
17.	Kim	Basic health, women's health, and mental health among internally displaced persons in Nyala Province, South Darfur, Sudan	2007
18.	Stark	Building caregivers' emotional, parental and social support skills to prevent violence against adolescent girls: findings from a cluster randomised controlled trial in Democratic Republic of Congo	2018
19.	Spittal	Cango Lyec (Healing the Elephant): Gender Differences in HIV Infection in Post-conflict Northern Uganda	2018
20.	Falb	Caregiver parenting and gender attitudes: Associations with violence against adolescent girls in South Kivu, Democratic Republic of Congo	2017
21.	Kelly	Characterizing Sexual Violence in the Democratic Republic of the Congo. Profiles of Violence, Community Responses, and Implications for the Protection of Women	2009
22.	Usta	Child sexual abuse in Lebanon during war and peace	2010
23.	Khawaja	Coerced sexual intercourse within marriage: a clinic-based study of pregnant Palestinian refugees in Lebanon	2008
24.	Patel	Comparison of HIV-related vulnerabilities between former child soldiers and children never abducted by the LRA in northern Uganda	2013
25.	McIlvaine	Confidence, Capacity Building and Cash: Achieving Sustained Impact for Ultra-poor Women	2015
26.	Avdibegovic	Consequences of domestic violence on women's mental health in Bosnia and Herzegovina	2006
27.	Oxfam in Pakistan	Consolidated Gender Analysis for Disaster Response in Pakistan	2017
28.	CARE Ethiopia	Consolidated Gender Analysis for the Ethiopian Drought Response	2016
29.	Hababeh	Contraceptive use by Palestine refugee mothers of young children attending UNRWA clinics: a cross-sectional follow-up study	2018
30.	Okanlawon	Contraceptive use: knowledge, perceptions and attitudes of refugee youths in Oru Refugee Camp, Nigeria	2010
31.	Al-Adili	Deaths among Young, Single Women in 2000-2001 in the West Bank, Palestinian Occupied Territories	2008
32.	Duroch	Description and consequences of sexual violence in Ituri province, Democratic Republic of Congo	2011
33.	Wanyama	Diriswanaag Final Evaluation	2013
34.	Stark	Disclosure bias for group versus individual reporting of violence amongst conflict-affected adolescent girls in DRC and Ethiopia	2017

35.	Lundgren	Does it take a village? Fostering gender equity among early adolescents in Nepal	2018
36.	Assaf	Domestic Violence Against Single, Never-Married Women in the Occupied Palestinian Territory	2013
37.	Hammoury	Domestic violence against women during pregnancy: the case of Palestinian refugees attending an antenatal clinic in Lebanon	2009
38.	Tappis	Domestic violence among Iraqi refugees in Syria	2012
39.	Khawaja	Domestic violence in refugee camps in Jordan	2004
40.	Bass	Economic, social and mental health impacts of an economic intervention for female sexual violence survivors in Eastern Democratic Republic of Congo	2016
41.	Undie	Effectiveness of a community-based SGBV prevention model in emergency settings in Uganda: Testing the 'zero tolerance village alliance' intervention	2016
42.	Tirivayi	Humanitarian Assistance through Mobile Cash Transfers: Emergency Cash-First Response to food security in drought-affected communities in Southern Zimbabwe through a mobile cash transfer project	2016
43.	Fleming	Engaging men and boys in advancing women's agency: Where we stand and new directions	2015
44.	Jabbar	Evaluating a vocational training programme for women refugees at the Zaatari camp in Jordan: women empowerment: a journey and not an output	2015
45.	Callands	Experiences and acceptance of intimate partner violence: associations with sexually transmitted infection symptoms and ability to negotiate sexual safety among young Liberian women	2013
46.	de Jong	Exposure to violence and PTSD symptoms among Somali women	2011
47.	Ssebunya	Factors associated with prior engagement in high-risk sexual behaviours among adolescents (10-19 years) in a pastoralist post-conflict community, Karamoja sub-region, North eastern Uganda	2019
48.	Gibbs	Factors associated with recent intimate partner violence experience amongst currently married women in Afghanistan and health impacts of IPV: a cross sectional study	2018
49.	Nattabi	Family planning among people living with HIV in post-conflict Northern Uganda: A mixed methods study	2011
50.	Kisindja	Family planning knowledge and use among women in camps for internally displaced people in the Democratic Republic of the Congo	2017
51.	Dossa	Fistula and Other Adverse Reproductive Health Outcomes among Women Victims of Conflict-Related Sexual Violence: A population-based Cross-sectional study	2014

52.	Seff	Forced Sex and Early Marriage: Understanding the Linkages and Norms in a Humanitarian Setting	2019
53.	Kelly	From political to personal violence: Links between conflict and non-partner physical violence in post-conflict Liberia	2019
54.	Wolfgang	From the Ground Up: Gender and conflict analysis in Yemen	2016
55.	Minza	Gender and Changes in Tsunami-Affected Villages in Nanggroe Aceh Darussalam province	2005
56.	International Rescue Committee	Gender Based Violence Among Displaced Communities in Sittwe Township, Rakhine state: A Knowledge, Attitudes and Practices Study	2016
57.	Wasilkowska	Gender Impact Analysis: Unconditional Cash Transfers in South Central Somalia	2012
58.	Thapa	Gender inclusiveness in disaster risk governance for sustainable recovery of 2015 Gorkha Earthquake, Nepal	2019
59.	Gupta	Gender norms and economic empowerment intervention to reduce intimate partner violence against women in rural Côte d'Ivoire: a randomized controlled pilot study	2013
60.	Slegh	Gender Relations, Sexual and Gender-Based Violence and the Effects of Conflict on Women and Men in North Kivu, Eastern Democratic Republic of the Congo: Results from the International Men and Gender Equality Survey (IMAGES)	2014
61.	UN Women	Gender-Based Violence and Child Protection Among Syrian Refugees in Jordan, with a Focus on Early Marriage	2013
62.	Faas	Gendered access to formal and informal resources in postdisaster development in the Ecuadorian andes	2014
63.	Metheny	Help Seeking Behavior among Women Who Report Intimate Partner Violence in Afghanistan: an Analysis of the 2015 Afghanistan Demographic and Health Survey	2019
64.	Goldenberg	High burden of previously undiagnosed HIV infections and gaps in HIV care cascade for conflict-affected female sex workers in northern Uganda	2019
65.	Family Health International	HIV and AIDS Behavioural Surveillance Survey (BSS): Refugee Camps and Hosting Communities in Kawambwa and Mporokoso, Zambia	2006
66.	Harrison	HIV behavioural surveillance among refugees and surrounding host communities in Uganda, 2006	2009
67.	Bermudez	HIV Risk Among Displaced Adolescent Girls in Ethiopia: the Role of Gender Attitudes and Self-Esteem	2019
68.	Amowitz	Human Rights Abuses and Concerns About Women's Health and Human Rights in Southern Iraq	2004

69.	CARE Afghanistan	CARE International in Afghanistan Humanitarian Program (HP): Emergency shelter, NFI, Hygiene, SRHR and Livelihood Support for Disaster-Affected Populations in Afghanistan - 2018-2020, Baseline/KAP Survey Report	2019
70.	McGinn	Improving refugees' reproductive health through literacy in Guinea	2006
71.	Erickson	Incarceration and exposure to internally displaced persons camps associated with reproductive rights abuses among sex workers in northern Uganda	2017
72.	Scott	Influences of sex, age and education on attitudes towards gender inequitable norms and practices in South Sudan	2014
73.	Okello	Baseline evaluation of integrated emergency response program for South Sudanese refugees and affected host community members in Rhino and Imvepi settlements, Arua District	2018
74.	Mansour	International Men and Gender Equality (IMAGES): Lebanon Summary	2017
75.	Erickson	Interpersonal and structural contexts of intimate partner violence among female sex workers in conflict-affected northern Uganda	2018
76.	Bhardwaj	Interpersonal violence and suicidality among former child soldiers and war-exposed civilian children in Nepal	2018
77.	Kidman	Intimate partner violence, modern contraceptive use and conflict in the Democratic Republic of the Congo	2015
78.	Cankaya	Investigation of social support perceptions and hopelessness levels of refugee women in Turkey	2018
79.	De Boodt	Final Evaluation of Kirumara II Project: Summary	2012
80.	Tanaka	Knowledge, attitude, and practice (KAP) of HIV prevention and HIV infection risks among Congolese refugees in Tanzania	2008
81.	Khalidi	Knowledge, Attitudes and Practices of Domestic Violence Among Refugee Communities in Lebanon: A Report of Baseline & Follow-up Surveys of A Sample of Association Najdeh Beneficiaries, 2001-2003	2004
82.	Wirtz	Lifetime prevalence, correlates and health consequences of gender-based violence victimisation and perpetration among men and women in Somalia	2018
83.	Patel	Lost in transition: HIV prevalence and correlates of infection among young people living in post-emergency phase transit camps in Gulu District, Northern Uganda	2014
84.	Parcesepe	Measuring Physical Violence and Rape Against Somali Women Using the Neighborhood Method	2016

85.	Stark	Measuring violence against women amidst war and displacement in northern Uganda using the "neighbourhood method"	2010
86.	Hossain	Men's and women's experiences of violence and traumatic events in rural Cote d'Ivoire before, during and after a period of armed conflict	2014
87.	Khan	Midterm Evaluation: Epidemic Control and Reinforcement of Health Services (ECRHS) Project in Sierra Leone	2017
88.	Groenewold	Millennium Development Indicators of employment and gender equality of Afghan refugees in Pakistan: Country report	2006
89.	Kolbe	Mortality, crime and access to basic needs before and after the Haiti earthquake: a random survey of Port-au-Prince households	2010
90.	Klasen	Multiple trauma and mental health in former Ugandan child soldiers	2010
91.	UN Women	Navigating through Shattered Paths: NGO Service Providers and Women Survivors of Gender-Based Violence, An Assessment of GBV Services in Gaza	2017
92.	Murphy	No safe place: A lifetime of violence for conflict-affected women and girls in South Sudan	2017
93.	Bartels	Now the world is without me: An investigation of sexual violence in Eastern Democratic Republic of Congo	2010
94.	Hirschfeld	Nowhere to turn: failure to protect, support and assure justice for Darfuri women	2009
95.	Ouma	Obstacles to family planning use among rural women of reproductive age in Atiak Health Centre IV, Amuru District, Northern Uganda	2015
96.	Loko Roka	One size fits all? Standardised provision of care for survivors of sexual violence in conflict and post-conflict areas in the Democratic Republic of Congo	2014
97.	United Nations Development Fund for Women	Pakistan Floods 2010: Rapid Gender Needs Assessment of Flood Affected Communities	2010
98.	Silove	Pathways to perinatal depressive symptoms after mass conflict in Timor-Leste: a modelling analysis using cross-sectional data	2015
99.	Bartels	Patterns of sexual violence in Eastern Democratic Republic of Congo: Reports from survivors presenting to Panzi Hospital in 2006	2010
100.	Shuman	Perceptions and Experiences of Intimate Partner Violence in Abidjan, Cote d'Ivoire	2016
101.	Orach	Perceptions, attitudes and use of family planning services in post conflict Gulu district, Northern Uganda	2015
102.	CARE Philippines	Baseline Study: Typhoon Haiyan Reconstruction Assistance in the Philippines	2017
103.	Al-Shdayfat	Physical Abuse Among Syrian Refugee Women In Jordan	2017

104.	Hudson	Picking up the pieces: Women's health needs assessment, Fond Parisien Region, Haiti	2010
105.	Muldoon	Policing the epidemic: High burden of workplace violence among female sex workers in conflict-affected northern Uganda	2017
106.	CARE Burundi	POWER Africa: Burundi Rolling Baseline Report 2015	2015
107.	Kennedy	Preliminary Impacts of an HIV-Prevention Program Targeting Out-of-School Youth in Postconflict Liberia	2018
108.	Stark	Prevalence and associated risk factors of violence against conflict-affected female adolescents: a multi-country, cross-sectional study	2017
109.	Saile	Prevalence and predictors of partner violence against women in the aftermath of war: a survey among couples in northern Uganda	2013
110.	Women's Wellness Center	Prevalence of gender-based violence: preliminary findings from a field assessment in nine villages in the Peja region, Kosovo	2006
111.	Al-Modallal	Prevalence of intimate partner violence among women visiting health care centers in Palestine refugee camps in Jordan	2015
112.	Delkhosh	Prevalence of intimate partner violence and reproductive health outcomes among Afghan refugee women in Iran	2019
113.	Khawaja	Prevalence of wife beating in Jordanian refugee camps: reports by men and women	2005
114.	Stark	Preventing violence against refugee adolescent girls: findings from a cluster randomised controlled trial in Ethiopia	2018
115.	Kinyanda	Psychiatric disorders and psychosocial correlates of high hiv risk sexual behaviour in war-affected Eastern Uganda	2012
116.	Loncar	Psychological consequences of rape on women in 1991-1995 War in Croatia and Bosnia and Herzegovina	2006
117.	Al-Modallal	Psychological partner violence and women's vulnerability to depression, stress, and anxiety	2012
118.	Vu	Psychometric properties and reliability of the Assessment Screen to Identify Survivors Toolkit for Gender Based Violence (ASIST-GBV): results from humanitarian settings in Ethiopia and Colombia	2016
119.	Miske Witt and Associates	The Power to Lead Alliance (PTLA): Empowering Girls to Learn and Lead, Final Evaluation Report for CARE USA	2011
120.	Glass	Randomised controlled trial of a livestock productive asset transfer programme to improve economic and health outcomes and reduce intimate partner violence in a postconflict setting	2017

121.	Falb	Recent abuse from in-laws and associations with adverse experiences during the crisis among rural Ivorian women: extended families as part of the ecological model	2013
122.	Benner	Reproductive health and quality of life of young Burmese refugees in Thailand	2010
123.	Pierce	Reproductive health care utilization among refugees in Jordan: Provisional support and domestic violence	2019
124.	Kottegoda	Reproductive health concerns in six conflict-affected areas of Sri Lanka	2008
125.	Howard	Reproductive health services for refugees by refugees in Guinea I: family planning	2008
126.	Balsara	Reproductive tract disorders among Afghan refugee women attending health clinics in Haripur, Pakistan	2010
127.	Scott	Respondent-driven sampling to assess mental health outcomes, stigma and acceptance among women raising children born from sexual violence-related pregnancies in eastern Democratic Republic of Congo	2015
128.	Tabbara	Restoring Dignity and Building Resilience: Monitoring Report of UN Women's Programming in Za'atari Refugee Camp, June-October 2015	2016
129.	Jeffrey	Restoring Livelihoods After Floods: Gender-sensitive response and community-owned recovery in Pakistan	2012
130.	Okigbo	Risk factors for transactional sex among young females in post-conflict Liberia	2014
131.	Kohli	Risk for family rejection and associated mental health outcomes among conflict-affected adult women living in rural eastern Democratic Republic of the Congo	2014
132.	Toma	Rohingya Refugee Response Gender Analysis: Recognizing and responding to gender inequalities	2018
133.	Hammoury	Screening for domestic violence during pregnancy in an antenatal clinic in Lebanon	2007
134.	CARE International	SDEARP Final Evaluation Report	2012
135.	Sumner	Sentinel events predicting later unwanted sex among girls: A national survey in Haiti, 2012	2015
136.	Ojengbede	Sexual and gender-based violence in camps for internally displaced people and host communities in northeast Nigeria: a mixed methods study	2019
137.	Parmar	Sexual violence among host and refugee population in Djohong District, Eastern Cameroon	2012
138.	Merten	Sexuality education is associated with increased disclosure and treatment seeking after violence experiences of young women 15-24: Results from a multi-country-survey including Eastern DRC, Rwanda, and Burundi	2015
139.	Duff	Social and structural factors increase inconsistent condom use by sex workers' one-time and regular clients in Northern Uganda	2018

140.	Story	Social capital and disaster preparedness in Oromia, Ethiopia: An evaluation of the "Women Empowered" approach	2018
141.	Oxfam	South Sudan Gender Analysis: A snapshot situation analysis of the differential impact of the humanitarian crisis on women, girls, men and boys in South Sudan	2017
142.	Albutt	Stigmatisation and rejection of survivors of sexual violence in eastern Democratic Republic of the Congo	2017
143.	Falb	Symptoms associated with pregnancy complications along the Thai-Burma border: the role of conflict violence and intimate partner violence	2014
144.	Dikmen	The attitudes of refugee women in Turkey towards family planning	2018
145.	Read-Hamilton	The Communities Care programme: changing social norms to end violence against women and girls in conflict-affected communities	2016
146.	Goessmann	The contribution of mental health and gender attitudes to intimate partner violence in the context of war and displacement: Evidence from a multi-informant couple survey in Iraq	2019
147.	Murphy	The effects of conflict and displacement on violence against adolescent girls in South Sudan: the case of adolescent girls in the Protection of Civilian sites in Juba	2019
148.	Gilbert	The experience of violence against children in domestic servitude in Haiti: Results from the Violence Against Children Survey, Haiti 2012	2018
149.	Murray	The impact of Cognitive Processing Therapy on stigma among survivors of sexual violence in eastern Democratic Republic of Congo: Results from a cluster randomized controlled trial	2018
150.	Mels	The psychological impact of forced displacement and related risk factors on Eastern Congolese adolescents affected by war	2010
151.	Landis	The school participation effect: investigating violence and formal education among girls in the Democratic Republic of the Congo	2018
152.	Annan	The state of female youth in northern Uganda: findings from the survey of war-affected youth (SWAY) phase II	2008
153.	Ibrahim	Trauma and perceived social rejection among Yazidi women and girls who survived enslavement and genocide	2018
154.	Managing Innovative Development Solutions – Consulting Services Inc.	Mid-Term Evaluation: Typhoon Haiyan Reconstruction Assistance Project, CARE Philippines	2017

155.	Center of Resources for Innovation and Development	Typhoon Haiyan Response Program: Summative Evaluation Response	2017
156.	Mansour	Understanding Masculinities: Results from the International Men and Gender Equality Survey (IMAGES) in Lebanon	2017
157.	El Feki	Understanding Masculinities: Results from the International Men and Gender Equality Survey (IMAGES) - Middle East and North Africa: Egypt, Lebanon, Morocco, and Palestine	2017
158.	Bell	Understanding the Effects of Mental Health on Reproductive Health Service Use: A Mixed Methods Approach	2016
159.	Gebrecherkos	Unmet need for modern contraception and associated factors among reproductive age group women in Eritrean refugee camps, Tigray, north Ethiopia: a cross-sectional study	2018
160.	Seyife	Utilization of modern contraceptives and predictors among women in Shimelba refugee camp, Northern Ethiopia	2019
161.	Sipsma	Violence against Congolese refugee women in Rwanda and mental health: a cross-sectional study using latent class analysis	2015
162.	Falb	Violence against refugee women along the Thai-Burma border	2013
163.	Campbell	Violence and abuse of internally displaced women survivors of the 2010 Haiti earthquake	2016
164.	Patel	War and HIV: sex and gender differences in risk behaviour among young men and women in post-conflict Gulu District, Northern Uganda	2014
165.	Catani	War Trauma, Child Labor, and Family Violence: Life Adversities and PTSD in a Sample of School Children in Kabul	2009
166.	Fulu	Why Do Some Men Use Violence Against Women and How Can We Prevent It? Quantitative Findings from the United Nations Multi-country Study on Men and Violence in Asia and the Pacific	2013
167.	Usta	Women, war, and violence: surviving the experience	2008
168.	Green	Women's entrepreneurship and intimate partner violence: A cluster randomized trial of microenterprise assistance and partner participation in post-conflict Uganda	2015
169.	Tomczyk	Women's reproductive health knowledge, attitudes and practices in relation to the Zika virus outbreak in northeast Brazil	2007
170.	Hossain	Working with men to prevent intimate partner violence in a conflict-affected setting: a pilot cluster randomized controlled trial in rural Côte d'Ivoire	2014
